# Supplementary material for: Evaluating the long-term consequences of air pollution in early life: geographical correlations between coal consumption in 1951/1952 and current mortality in England and Wales
Source: BMJ Open. 2018 Apr 27;8(4):e018231. doi: 10.1136/bmjopen-2017-018231 (PMC5922482; doi:10.1136/bmjopen-2017-018231)
Supplement: Supplementary file 1 [file bmjopen-2017-018231supp001.pdf]

Appendix

Cause of death	ICD 9 code	ICD 10 code	Number of deaths
**Cardiovascular disease**			
Ischaemic heart disease	410-414	I20-I25	710,979
Rheumatic heart disease	393-398	I05-I09	9,639
Cerebrovascular disease	430-438	I60-I69	264,853
Hypertension	401-405	I10-I15	84,208
**Respiratory disease**			
Chronic bronchitis and emphysema	490-492,496	J40-J44	105,690
Asthma	493	J45-J46	10,486
Pneumonia	480-486	J12-J18	107,848
Tuberculosis	010-018,137	A15-A19,B90	3,784
**Cancers**			
Lip, oral cavity and pharynx	140-149	C00-C14	22,992
Oesophagus	150	C15	65,854
Stomach	151	C16	49,634
Colon	153	C18	85,176
Rectosigmoid junction, rectum & anus	154	C19-C21	49,649
Liver, gall bladder and bile ducts	155,156	C22 – C24	31,240
Pancreas	157	C25	66,273
Larynx	161	C32	8,909
Trachea, bronchus & lung	162	C33,c34	335,217
Malignant melanoma	172	C43	20,045
Breast	174-175	C50	128,494
Cervix	180	C53	12,947
Uterus	179,182	C54-C55	14,315
Ovary	183	C56	47,895
Prostate	185	C61	54,521
Kidney	189	C64	33,232
Bladder	188	C67	32,353
Brain, Eye & CNS	191-192	C69-C72	43,811
Hodgkin’s disease	201	C81	3,096
Non-Hodgkin’s lymphoma	200,202	C82-C85	40,783
Multiple myeloma	203	C90	21,510
Leukaemia	204-208	C91-C95	33,526
