# Supplementary material for: Evaluating the long-term consequences of air pollution in early life: geographical correlations between coal consumption in 1951/1952 and current mortality in England and Wales
Source: BMJ Open. 2018 Apr 27;8(4):e018231. doi: 10.1136/bmjopen-2017-018231 (PMC5922482; doi:10.1136/bmjopen-2017-018231)
Supplement: Supplementary file 2 [file bmjopen-2017-018231supp002.pdf]

Appendix 2

The Office of National Statistics provided latitude and longitude measurements for each of the 342 areas used in our analyses, and from these we derived inter-area distances in miles. We compared various simple models for the autocorrelation function, and found that the function which fitted the data best is given by

$$\rho(d) = \exp(-\lambda d^2)$$

Where d is the distance areas and λ is a parameter. The maximum likelihood estimate λ_{max} was -0.0772 with 95% profile likelihood based confidence limits of -0.0986 and -0.0647. This autocorrelation function is graphed in the Figure below. The autocorrelation is close to zero above distances of 10 miles. 107 of the 58,311 inter-area distances (0.2%) are below 5 miles and 586 (1.0%) are below 10 miles.

As described in the methods section, the coal consumption data (x) for each area ($x_1, x_2, \ldots\ldots x_{342}$) used in Table 2 were transformed using a Fisher-Yates normal transformation. Tabulating the mean values of the product $x_i x_j$ in bands derived according to the value of the inter-area distance d_{ij} confirms that the correlation falls rapidly in this way.

Calculations that incorporate the autocorrelation term λ_{max} into regression analyses suggest that the variance of regression coefficients will increase by about 14.8%, and hence that standard errors will increase by about 7.1%. Confidence interval widths will rise accordingly.

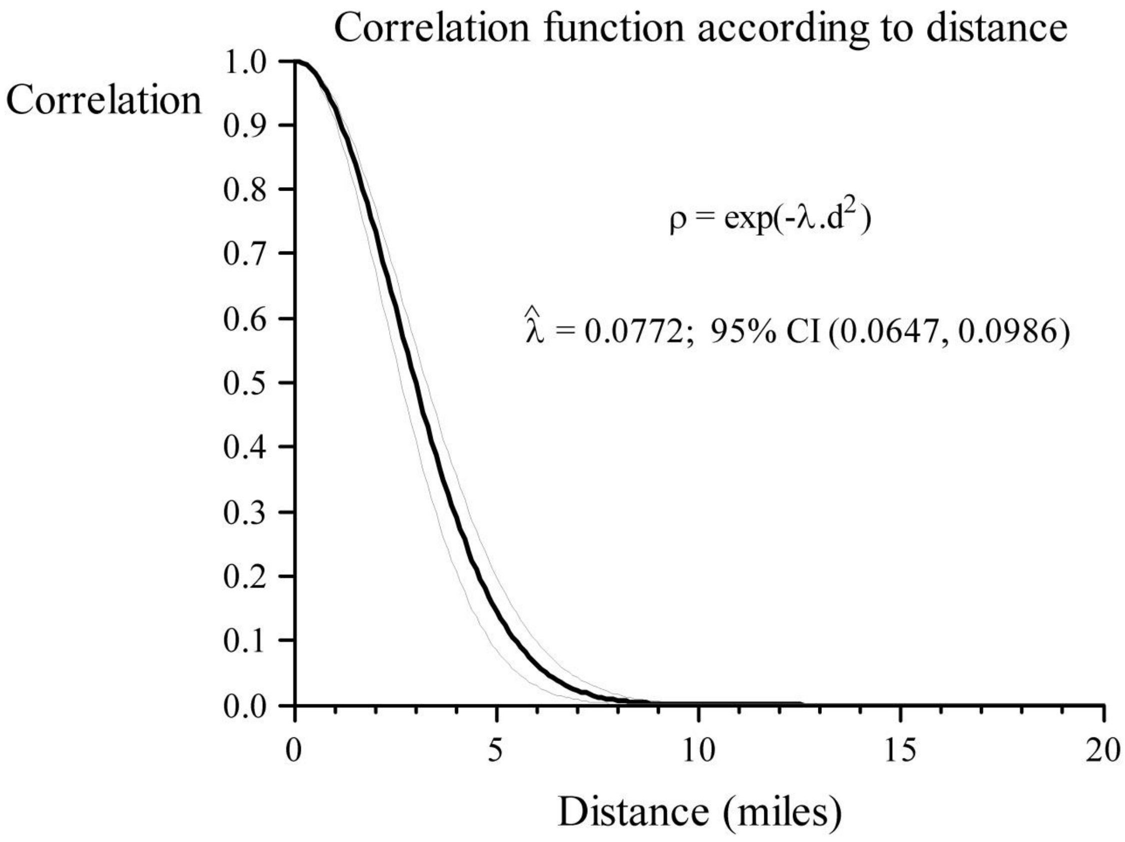
Correlation function according to distance
Correlation
1.0
0.9
0.8
0.7
0.6
0.5
0.4
0.3
0.2
0.1
0.0
ρ = exp(-λ.d²)
λ̂ = 0.0772; 95% CI (0.0647, 0.0986)
0
5
10
15
20
Distance (miles)
