# Supplementary material for: Evaluating the long-term consequences of air pollution in early life: geographical correlations between coal consumption in 1951/1952 and current mortality in England and Wales
Source: BMJ Open. 2018 Apr 27;8(4):e018231. doi: 10.1136/bmjopen-2017-018231 (PMC5922482; doi:10.1136/bmjopen-2017-018231)
Supplement: Author's manuscript [file bmjopen-2017-018231.draft_revisions.pdf]

BMJ Open is committed to open peer review. As part of this commitment we make the peer review history of every article we publish publicly available.

When an article is published we post the peer reviewers' comments and the authors' responses online. We also post the versions of the paper that were used during peer review. These are the versions that the peer review comments apply to.

The versions of the paper that follow are the versions that were submitted during the peer review process. They are not the versions of record or the final published versions. They should not be cited or distributed as the published version of this manuscript.

BMJ Open is an open access journal and the full, final, typeset and author-corrected version of record of the manuscript is available on our site with no access controls, subscription charges or pay-per-view fees (http://bmjopen.bmj.com).

If you have any questions on BMJ Open's open peer review process please email info.bmjopen@bmj.com

BMJ Open

Six decades after the Clean Air Act: evaluating the health legacy of Coal.

Journal:	*BMJ Open*
Manuscript ID	bmjopen-2017-018231
Article Type:	Research
Date Submitted by the Author:	19-Jun-2017
Complete List of Authors:	Phillips, David; Medical Research Council<br>Osmond, Clive; Medical Research Council, Epidemiology Resource Centre<br>Southall, Humphrey; University of Portsmouth, Dept of Geography<br>Aucott, Paula; University of Portsmouth, Dept of Geography<br>Jones, Alexander; University of Oxford, Dept of Paediatrics<br>Holgate, Stephen; Southampton General Hospital, Medicine and Therapeutics
Keywords:	air pollution, Mortality, Developmental origins of health and diseasee

SCHOLARONE™
Manuscripts

For peer review only

Six decades after the Clean Air Act: evaluating the health legacy of Coal.

David IW Phillips, professor[1];Clive Osmond, professor of medical statistics[1]; Humphrey Southall, professor in historical geography[2]; Paula Aucott, research fellow[2]; Alexander Jones, paediatric cardiologist[3] ;Stephen T Holgate, professor of immunopharmacology.[4]

From

[1]The Medical Research Council's Lifecourse Epidemiology Unit, University of Southampton, Southampton, SO16 6YD. UK. ; [2]Department of Geography, University of Portsmouth, Portsmouth, PO1 2UP, UK. ; [3]Department of Paediatrics, University of Oxford, Oxford, OX3 9DU, UK. [4]Department of Medicine, University of Southampton, Southampton, SO16 6YD, UK.

Correspondence: DIW Phillips, MRC Lifecourse Epidemiology Unit, Southampton General Hospital, Southampton, UK.

Email: diwp@mrc.soton.ac.uk

Tel +44 (0) 23 80777624

FAX + 44 (0) 23 80704021

Short title: Long term effects of coal consumption

For peer review only

ABSTRACT

Objective

The short-term effects of air pollution are well-documented, but less is known as to whether early life pollutant exposure has long-term adverse effects.

Design

Geographical study

Setting

Local authority areas in England and Wales

Exposures

Routinely collected data on the use of coal and related solid fuels in 1951-2 were used as an index of air pollution from domestic and industrial sources in different geographical areas of England and Wales.

Main outcome measures

We evaluated the relationship between these data and both all-cause and disease-specific mortality in the same geographical areas in 1993-2012.

Results

Domestic fuel, largely coal, had the most powerful effects on mortality probably because much industrial pollution was successfully vented through chimneys. There were strong relationships between domestic fuel use and all-cause mortality (relative risk per SD increase in fuel use 1.124, 95% CI = 1.123-1.126), and with respiratory (1.238, 1.234-1.242), cardiovascular (1.138, 1.136-1.140), and cancer mortality (1.073, 1.071-1.075). Following adjustment for social class, education, crowding, unemployment and current pollution levels, the effect of domestic consumption persisted.

Conclusion

Coal was the major cause of pollution in the UK until the Clean Air Act of 1956 led to a rapid decline in consumption. Although based on geographical correlations, our data raise the possibility that coal-based pollution, experienced over 60 years ago in young children, affects human health now, by increasing mortality from a wide variety of diseases.

For peer review only - http://bmjopen.bmj.com/site/about/guidelines.xhtml

Strengths and limitations of this study

•The first to evaluate the very long-term (>60 year) consequences of air pollution experienced in early life.

•Use of national mortality data with virtually complete ascertainment and a large number of deaths (> 3.5 million).

•Coal consumption data provides an integral of air pollution over a 12 month period.

•The analysis also allows for current pollution levels

•The study depends on the use of geographical correlations together with census-derived measures of socio-economic status; individual-level data are not available.

For peer review only

For peer review only - http://bmjopen.bmj.com/site/about/guidelines.xhtml

INTRODUCTION

Air pollution has detrimental effects on health at all ages but little is known about the long-term impact of early life exposure to pollutants.[1] This is despite accumulating evidence that the young are especially vulnerable. Developing tissues and organs are particularly susceptible due to their high cell proliferation rates in prenatal life. Postnatally, infants and young children also have increased vulnerability due to immature detoxification systems, high infection rates and patterns of behaviour that increase their pollutant exposure.[1] Pregnant mothers exposed to air pollution are more likely to give birth prematurely and have offspring with intrauterine growth retardation and poor growth in infancy.[2 3] Such outcomes are linked with long-term adverse health effects, including increased risks of respiratory and cardiovascular disease.[4]

In common with many other countries, the UK had high levels of air pollution in the past.[5] Most of this was due to coal use in industry and as a domestic fuel, which gave rise to the "great stinking fogs" that previously characterised London and other major cities.[5] The London smog of 1952 killed an estimated 4,000 people[6] and led to the first UK Clean Air Act being passed in 1956. Following the 1952 smog, a national survey of smoke and SO_2 concentrations was set up during the 1960s to monitor the progress of pollution control following the Act. From around 1960, there was a progressive reduction in black smoke and SO_2 emissions, with nitrogen oxides, ozone and microparticulates from transport becoming the dominant forms of air pollution.[7]

Little is known about the long-term health effects of the combustion of the vast quantities of coal burnt which still exceeded 200 million tons per year in England and Wales at the middle of the 20th century.[8] Studies published in the 1950s and 1960s suggested that there were strong cross-sectional correlations between the amounts of coal used in domestic fires and childhood respiratory disease [9] or other major causes of mortality in adults[10 11] but it is not known if these have persisted. The present study is based on a dataset compiled by the Ministry of Fuel and Power that recorded the quantities of different types of solid fuel burnt annually between May 1951 and May 1952 in 1,145 areas of England and Wales. The extent to which exposure to the resulting pollutants may have influenced present day health was assessed by relating these data to mortality rates in the same areas 50-60 years later.

For peer review only - http://bmjopen.bmj.com/site/about/guidelines.xhtml

METHODS

Shortages of coal following the Second World War led to the continuation of a system of rationing and distribution that had been initiated in 1939. This system was controlled by local fuel overseers (LFOs) who were appointed for each local government district (LGD) or group of districts. The Ministry of Fuel and Power published estimates of the quantities of different types of solid fuel burnt annually in each of the 1,145 LFO areas between May 1951 and May 1952 in England and Wales – data were only published for these years.[12] Domestic supplies included coal, the amounts used in each LFO area were based on the number of registered premises, and other solid fuels including coke, briquettes, ovoids and similar patent fuels where usage was based on merchant's disposals. The annual household coal allowance was higher in the North (2,540 kg) than the South (1,727 kg). Miners' concessionary coal was also included. Industrial fuels were coal and coke and there were separate listings of the quantities of fuel used in electricity generation and for carbonization or briquetting plants. The amounts of smokeless fuel used were recorded separately.

The Office for National Statistics (ONS) provided extracts from all death certificates in England and Wales for each of the current LGDs during 1993 to 2012. This is the period covered by the ninth and tenth revisions of the International Classification of Disease (ICD). The ICD codes used to define causes of death are given in Appendix 1. Mortality rates were calculated using population estimates provided by the ONS for each LGD. The main technical problem was to relate the historical 1,145 LFO areas to the current 342 LGDs, following successive local government reorganisations in 1974, the 1990s, and 2009, together with numerous individual boundary changes. Detailed digital boundaries for the LGDs of England and Wales used in the 1951 census have already been constructed as part of the Great Britain Historical Geographical Information System (GIS).[13 14] As the LFO areas either exactly corresponded to LGDs or were defined as aggregates of them, constructing digital boundary data for LFO areas was straightforward. However, modern LGDs are not necessarily simple aggregates of the LFO areas, so the first stage of the redistricting procedure was to re-allocate the fuel consumption data to the 12,504 Civil Parishes of 1951. Digital boundaries already existed for the parishes, and the re-allocation was based on what proportion each parish's total population, as listed by the 1951 census, was of the overall population of the containing LFO area. The fuel data were then re-allocated to the modern areas by digitally overlaying the two sets of boundaries: whatever proportion of a 1951 parish's area fell within a modern district, that proportion of the parish's fuel consumption was assigned to the district.

Historically, air pollution was worse in predominantly industrial towns with a high population density; these towns also tended to have large populations of factory workers who left school early and worked

For peer review only - http://bmjopen.bmj.com/site/about/guidelines.xhtml

in low status occupations.[10] These are important potential confounders in a study of air pollution and disease, as poverty and overcrowding are known to be associated with many of the major causes of mortality. We used a series of variables extracted from the published reports of the 1951 census: male unemployment; indices of social class, which were based on a weighted average of the proportions of each of five social classes within each area; overcrowding, assessed as the proportion of households with more than one person per room; density, the number of people per acre; and educational achievement, the proportion of employed men in each area who left school before the age of 15 years. We also allowed for the impact of current levels of air pollution which were obtained from published data reporting the modelled annual average concentrations of fine particulate matter ($PM_{2.5}$) in each of the 342 LGDs.[15]

Statistical methods

We studied deaths in people aged between 35 and 74 years, as this was the approximate age range of the generation who were infants or young children in 1951-52, and excluded older age groups where the cause of death was less likely to have been ascertained accurately.[16] We calculated the number of deaths expected for each five-year age and sex group within the LGDs by multiplying the number of people in each group by the national age- and sex- specific death rates in 1993-2012. We expressed the number of observed deaths in each area as a percentage of the expected deaths for that group, i.e. standardised mortality ratios (SMRs). We used Poisson regression to model SMRs expressing the effect of fuels and other variables on mortality in terms of the hazard ratio (or relative risk) per SD change in the explanatory variable. This weights the analyses appropriately to allow for variation in population size across LGDs. A Fisher-Yates transform was used to standardise pollution and socioeconomic variables. This yielded variables with zero means, unit standard deviations (SDs) and symmetrical distributions, allowing direct comparison of the strength of associations with these variables.

Patient involvement

No patients were involved in setting the research question or the outcome measures, nor were they involved in developing plans for design or implementation of the study. No patients were asked to advise on interpretation or writing up of results. The analysis was based on geographical death rates and no individuals were identifiable.

For peer review only

For peer review only - http://bmjopen.bmj.com/site/about/guidelines.xhtml

RESULTS

There were a total of 3,535,136 deaths in England and Wales during the 20-year period from 1993 to 2012 in the 35 to 74 yr. age-group. Mortality rates (SMR) in the 342 LGDs ranged from 64.7 in East Dorset to 153.1 in Manchester. Table 1 shows the different uses of solid fuel during 1951/2 in England and Wales. Of the over 200 million tons distributed, 37.6% was used in industrial processes, 16.6% in electricity generation and 27.5% in carbonization or briquetting processes. Only 18.3% was used domestically. Smokeless fuel accounted for approximately 32.1% of industrial and 14.3% of domestic consumption. Figure 1 is a map of England and Wales showing the quintiles of domestic fuel consumed per acre. This was generally low in rural areas but was greatest in London and in the former industrial areas of the UK (South Wales, the Midlands, Lancashire, the West Riding of Yorkshire and Tyneside). The mean current $PM_{2.5}$ concentrations in the 342 areas was 9.48 (SD 1.67) μg/m^3 and ranged from 5.5 in Gwynedd (Wales) to 14.9 μg/m^3 in the City of Westminster, London.

Table 2 shows the relationship between the various sources of fuel burnt in 1951/2 (excluding smokeless fuels) and subsequent all-cause mortality or mortality due to respiratory, cardiovascular disease and cancer in the 342 areas. The influence of socioeconomic indicators (social class, education, crowding, density and unemployment) are also shown together with the levels of current pollution ($PM_{2.5}$). In univariate analyses, all variables were significantly associated with both all-cause and cause-specific mortality rates although the effects of domestic and industrial consumption tended to be most strongly related to the mortality outcomes. In multivariate analyses, however, domestic consumption was strongly and significantly associated with both all-cause and cause-specific mortality rates, an effect which was markedly stronger than any other variable included in the regression analysis. There was a very high correlation (r=0.97) between domestic consumption and population density, resulting in unacceptable collinearity in regression analyses that contained both variables. This occurred because both had the same denominator, the area of the LGD. To allow the influence of both of these factors to be modelled statistically, we created two further variables: the sum of and the difference between domestic use and density. These variables were uncorrelated (r=0.0). These were entered into the regression models in Table 2, which shows both the unadjusted coefficients and the coefficients adjusted for all other variables (Table 2 lower panel). The positive regression coefficients for the sum variable show that the joint effects of domestic use and density on all four causes of mortality were powerful. However, associations with the difference variable suggest that the influence of domestic use was stronger than that of density.

For peer review only - http://bmjopen.bmj.com/site/about/guidelines.xhtml

Smokeless fuels represented a small proportion of the total fuels used and contributed little to the mortality risks. The effects of the potential confounders were tested singly and in combination. There was no evidence of non-linear relationships. Figure 2 shows the associations between quartiles of domestic fuel consumption and all-cause mortality or the major mortality categories. These associations were progressive and remained strong and statistically significant for each cause group after adjustment for social class, education, crowding, unemployment and current $PM_{2.5}$. They were present in both genders and were similar during the ICD 9 and ICD 10 periods.

Figure 3 shows the strength of the geographical correlations between domestic consumption and current mortality according to successive birth cohorts. For all-cause and respiratory mortality, the strongest associations were observed among those born in 1952/3 suggesting that exposure at around the time of birth had the greatest effect on mortality. For cardiovascular and cancer mortality, the peak associations were more blunted suggesting that exposure over a wider age range was associated with subsequent mortality.

Tables 3 & 4 show the relationship between domestic fuel consumption and specific causes of mortality. As before, the data are shown before and after adjustment for the four socioeconomic indicators and current $PM_{2.5}$ levels. The respiratory conditions responsible for most deaths, chronic obstructive pulmonary disease and pneumonia, were strongly and consistently associated while associations with asthma mortality were somewhat weaker. However, the largest relative risks were with tuberculosis. While all four major causes of cardiovascular mortality were significantly associated with domestic consumption, the strongest relationship was with rheumatic heart disease.

The data on associations of domestic fuel use with mortality from specific cancers were striking (Table 4). There were strong relationships with epithelial cancers; lip, oral cavity and pharynx (adjusted hazard ratio, 1.283); laryngeal cancers (1.323) and lung (1.219). The upper gastrointestinal cancers; oesophagus (1.131), stomach (1.166) and liver (1.174), were more strongly associated with domestic pollution than the lower gastrointestinal cancers. There were weak associations with bladder and kidney cancers. In contrast, the major reproductive and haematological cancers showed no increased risk, with the exception of cervical cancer (1.229). Melanoma and brain cancer were associated with a reduced relative risk.

For peer review only - http://bmjopen.bmj.com/site/about/guidelines.xhtml

DISCUSSION

Areas of the UK which had high domestic consumption of coal and related non-smokeless solid fuels in 1951/52 currently have raised mortality from a wide variety of different causes including cardiovascular or respiratory disease and certain cancers. The correlations were strong, statistically significant and independent of the available variables that might be considered likely major confounders such as social class, level of education, overcrowding, unemployment and density. They were also independent of current levels of air pollution as indicated by current concentrations of microparticulate pollution ($PM_{2.5}$).

Very few comparable data exist in the literature, particularly with such a long follow-up period. However, our findings accord with a body of evidence that early life exposure to air pollution has detrimental long-term health effects.[11 17] In particular, two recent studies demonstrated important findings. In the first, children exposed to the great London Smog of 1952 were found to have greater risk of asthma in adulthood, compared to unexposed children.[18] In the second, which was based on the ONS longitudinal study, air pollution concentrations at the site of residence, assessed every 10 years from 1971, were associated with increased total, respiratory and cardiovascular mortality.[19] Our conclusions also accord with historical studies of coal use and mortality in Britain which estimate that each SD increase in coal use raised mortality by 5-15% in infants and 5% in adults.[20]

Our study depended on coal consumption as an index of domestic air pollution as it is not unreasonable to assume that the quantity of coal burnt relates to the amount of pollution emitted. The consumption measures in our study were shown to correlate strongly with subsequent measurements of pollution in the UK,[9] and this approach is also validated by a number of studies carried out over the last 60 years showing correlations with diverse health outcomes including reduced early growth,[2 3] chronic bronchitis and lung cancer.[9-11] In the post-war years, economic necessity drove the use of low-grade, bituminous domestic coal while better-quality "hard" coals were usually exported. A typical, inefficient domestic grate burning low-grade coal would have produced lots of smoke, rich in a wide variety of potentially toxic compounds, including heavy metals, sulphur and complex mixtures of aliphatic and aromatic hydrocarbons.[21] Confirmation of this can be found in the lung tissue from autopsies of people exposed to the 1952 London smog which was shown to contain both ultrafine carbonaceous and metal particulate matter.[22] Children would have been exposed both in the home and in their local environment although it is not clear from our data which would have been the most important source. However, our study does suggest that it was domestic exposure that had the greatest long-term adverse health effects, with industrial pollution or pollution from power stations or other sources causing relatively smaller effects (Table 2). The probable explanation for this is that much industrial pollution was generally vented

For peer review only - http://bmjopen.bmj.com/site/about/guidelines.xhtml

through tall chimneys, decreasing ground-level pollution in approximate proportion to the inverse square of chimney height.[23]

Current levels of pollution in the 342 local areas were based on ambient levels of fine particulate matter ($PM_{2.5}$); the results of large cohort studies suggest that these levels are most closely associated with long-term health effects on pollution.[24-26]. Although the effects on current mortality (Table 2) are consistent with published estimates[15] in the inclusive models with early coal consumption, there was no separate effect observed on mortality.

Our data do not allow determination of the age at which children were most vulnerable to the effects of pollution although the cohort-based analysis (Figure 3) suggests that exposures at around the time of birth or infancy are likely to be most important especially for all-cause and respiratory mortality. Although the consumption data come from a short time-period between 1951 and 1952, they are likely to reflect smoke emission over many years. UK coal production was fairly constant during the late 1940s, declined a little during the 1950s and only fell dramatically after 1960.[8] We can also only speculate on the mechanism by which air pollution arising from coal consumption, predominantly in a domestic setting, might increase the risk of mortality. There may have been direct mutagenic effects especially in relation to the increased cancer risk. Another possibility is that the pollutants triggered mechanisms that selected alternative developmental pathways in the young, perhaps through epigenetic modification of gene expression.[27]

Elevated relative risks were observed for respiratory diseases, especially COPD, asthma and pneumonia, which is consistent with past evidence that black smoke exposure is a risk factor for these conditions.[10 11 17] The strongest association we found, however, was with tuberculosis, which is less supported by existing literature. A recent systematic review and meta-analysis concluded that the evidence for such an association was inconclusive but it was reliant on case-control and cross-sectional studies.[28] The findings in the present study raise the possibility that early exposure to air pollution could be a critical factor in determining susceptibility to tuberculosis. Cardiovascular disease was also found to be strongly associated with domestic fuel consumption which accords with earlier UK findings [11 17] the Harvard Six Cities study [25] and a multicentre study of European Cohorts.[29] The strength of the association was similar with ischaemic heart disease and stroke. The association with rheumatic heart disease was strong and confirms a previous study based on a subset of large Country Boroughs in England and Wales.[30] An additional finding was a strong association with hypertension, which is supported by a meta-analysis that showed both short-term and long-term exposure to some air pollutants increases the risk of hypertension.[31] The data on cancers (Table 4) show wide variation in the level of risk associated. They suggest that the strongest associations are observed with the epithelial cancers of the respiratory system (lip, oral cavity and pharynx; larynx; trachea and lung) and the upper gastrointestinal system (stomach

For peer review only - http://bmjopen.bmj.com/site/about/guidelines.xhtml

and liver). The major reproductive and haematological cancers were not positively associated with domestic pollution with the exception of cervical cancer, for which there is evidence that cigarette smoking is an important co-factor.[32] Malignant melanoma had a lower relative risk in the areas with high consumption possibly because of reduced sunlight exposure, while the reason for reduced relative risk observed with brain cancer is not clear, but may be due to residual confounding.

There are a number of potential limitations to our study. An inevitable consequence of evaluating early effects on major causes of late life mortality is the long latency between the measurements of coal consumption and the mortality data. It could be argued that internal migration might have weakened the associations that we observed as selective migration has been proposed as an explanation of geographical variations in mortality which is supported by some studies[33] but not others.[34 35] Migration is complex as while migrants are generally healthier, those moving short distances tend to have higher mortality than those moving long distances.[36 37] Although we do not have migration data, during the time period of this study most migration occurred over short distances with only a small minority migrating significant distances.[38] A more recent study of migration based on the 2001 census, while documenting the complex age and social class influences on migration, suggests that the declining industrial areas of South Wales, Yorkshire, Greater Manchester and Lancashire, and the North-East of England, which were the areas that had high coal consumption, had very much below average in-migration and out-migration for all age groups.[39] In addition, our analysis allows for the factors which tend to drive migration such as education and socioeconomic status.[40] Finally, it is difficult to see how selective migration could explain the specific links between coal consumption and some conditions and not others (Table 4). Any undetected bias in our observational study would be most likely to be due to confounding from an unknown source. However, a sensitivity analyses suggested that such a variable would have to correlate very strongly (r >0.5) with both domestic air pollution and respiratory disease, for example, to confound the relationships we have observed. We have examined the effects of the major confounding variables that were available in the 1951 census and that are likely to be related to both residence in an area of high pollution and to the various outcomes that we have studied. The 1951 census contained several measures of household amenities, which were also examined in regression analyses. We found that these did not explain any further variance in our analyses beyond that due to the confounding variables that we used in the final analyses. Adjusting the risk estimates for social class, density, unemployment and education reduced their magnitude marginally but they but still remained strongly significant (Table 2 and Figure 2). However, it could be argued that some of these variables are so closely related to the measures of pollution that the regression based adjustment has resulted in conservative risk estimates. Unsurprisingly, population density was highly correlated with domestic fuel use because fuel was rationed on a per household basis. In our analyses, we were able to show that pollution had a more marked effect than population density but the close relationship between these

For peer review only - http://bmjopen.bmj.com/site/about/guidelines.xhtml

variables prevented us from using this in the adjusted regression models. Areal indices of density may be less important than overcrowding in determining the health outcomes associated with high population density.[41] Although we did not have data on the prevalence of tobacco smoking, we do not think that this is likely to have seriously confounded our results as firstly, smoking is associated with low socioeconomic status[42] which was included in our analyses and secondly standardisation of the data for lung cancer rates, as a proxy for smoking, did not explain the correlations we observed.

Conclusions

Although this is an ecological study based on geographical correlations, the results raise the possibility that domestic air pollution, experienced over 60 years ago in young children, affects human health now, by increasing mortality from a wide variety of diseases. The effect of early life exposure was much stronger than that of current pollution assessed by concentrations of microparticulates. Our findings have important implications for the many countries that still depend on large amounts of coal for their domestic markets and where inefficient coal fires and their associated pollution are common.

Acknowledgments: The authors are grateful to Laura Todd and her colleagues at the UK Office for National Statistics for their help in providing the mortality data.

For peer review only

For peer review only - http://bmjopen.bmj.com/site/about/guidelines.xhtml

REFERENCES

1. WHO. Effects of Air Pollution on Children's Health and Development. European Centre for Environment and Health. Bonn: WHO, 2005:1-184.
2. Bobak M, Richards M, Wadsworth M. Air pollution and birth weight in Britain in 1946. *Epidemiology* 2001;12(3):358-59.
3. Bobak M, Richards M, Wadsworth M. Relation between children's height and outdoor air pollution from coal-burning sources in the British 1946 birth cohort. *IntArchOccupEnvironHealth* 2004;77(6):383-86.]
4. Barker DJP. The Wellcome Foundation Lecture, 1994. The fetal origins of adult disease. *Proc Roy Soc Lond* 1995;262:37-43.
5. Brimblecombe P. The Big Smoke: Methuen 1987:1-204.
6. Logan WP. Mortality in the London fog incident, 1952. *Lancet* 1953;1(6755):336-8.
7. Ayres JG. Trends in air quality in the UK. *Allergy* 1997;52(38 Suppl):7-13.
8. Department for Business Energy & Industrial Strategy. Historical coal data: coal production, availability and consumption 1853 to 2015. https://www.gov.uk/government/statistical-data-sets/historical-coal-data-coal-production-availability-and-consumption-1853-to-2015.
9. Douglas JWB, Waller RE. Air Pollution and respiratory infection in children. *Br J Prev Soc Med* 1966;20:1-8.
10. Daly C. Air pollution and causes of death. *Br J Prev Soc Med* 1959;13(1):14-27.
11. Gardner MJ, Crawford MD, Morris JN. Patterns of mortality in middle and early old age in the county boroughs of England and Wales. *Br J Prev Soc Med* 1969;23(3):133-40.
12. Ministry of Fuel and Power. Statistical Digest, 1954. London 1955.
13. Gregory IN, Southall HR. Putting the Past in Its Place: the Great Britain Historical GIS In: Carver S, ed. Innovations in GIS 5: Taylor & Francis 1998:210-21.
14. Southall H. Rebuilding the Great Britain Historical GIS, Part 2 A Geo-Spatial Ontology of Administrative Units. *Hist Method* 2012;45(3):119-34.
15. Gowers AM, Miller BG, Stedman JR. Estimating local mortality burdens associated with particulate air pollution. In: England PH, ed. London: Public Health England, 2014:1-40.
16. Paterson DA, Dorovitch MI, Farquhar DL, et al. Prospective study of necropsy audit of geriatric inpatient deaths. *J Clin Pathol* 1992;45(7):575-8.
17. Elliott P, Shaddick G, Wakefield JC, et al. Long-term associations of outdoor air pollution with mortality in Great Britain. *Thorax* 2007;62(12):1088-94. doi: 10.1136/thx.2006.076851
18. Bharadwaj P, Graff Zivin J, Mullins JT, et al. Early Life Exposure to the Great Smog of 1952 and the Development of Asthma. *American journal of respiratory and critical care medicine* 2016 doi: 10.1164/rccm.201603-0451OC
19. Hansell A, Ghosh RE, Blangiardo M, et al. Historic air pollution exposure and long-term mortality risks in England and Wales: prospective longitudinal cohort study. *Thorax* 2016;71(4):330-38.
20. Beach B, Walker Hanlon W. Coal smoke and mortality in an early industrial economy, 2016:1-75. http://www.econ.ucla.edu/whanlon/papers/beach_hanlon_draft.pdf
21. IARC. Indoor emissions from household combustion of coal. Lyon, France: WHO, 2012:515-38.
22. Hunt A, Abraham JL, Judson B, et al. Toxicologic and epidemiologic clues from the characterization of the 1952 London smog fine particulate matter in archival autopsy lung tissues. *Environmental health perspectives* 2003;111(9):1209-14.
23. Williams ML. Atmospheric dispersal of pollutants and the modelling of air pollution. In: RM H, ed. Pollution, causes, effects and control. 5th ed. London: Royal Society of Chemistry 2015:225-43.
24. Dockery DW, Pope CA, 3rd, Xu X, et al. An association between air pollution and mortality in six U.S. cities. *N Engl J Med* 1993;329(24):1753-9.
25. Laden F, Schwartz J, Speizer FE, et al. Reduction in fine particulate air pollution and mortality: Extended follow-up of the Harvard Six Cities study. *American journal of respiratory and critical care medicine* 2006;173(6):667-72.
26. Pope CA, 3rd, Burnett RT, Thun MJ, et al. Lung cancer, cardiopulmonary mortality, and long-term exposure to fine particulate air pollution. *JAMA* 2002;287(9):1132-41.

For peer review only - http://bmjopen.bmj.com/site/about/guidelines.xhtml

27. Godfrey KM, Costello PM, Lillycrop KA. The developmental environment, epigenetic biomarkers and long-term health. *Journal of developmental origins of health and disease* 2015;6(5):399-406.
28. Lin HH, Suk CW, Lo HL, et al. Indoor air pollution from solid fuel and tuberculosis: a systematic review and meta-analysis. *The international journal of tuberculosis and lung disease : the official journal of the International Union against Tuberculosis and Lung Disease* 2014;18(5):613-21.
29. Wolf K, Stafoggia M, Cesaroni G, et al. Long-term Exposure to Particulate Matter Constituents and the Incidence of Coronary Events in 11 European Cohorts. *Epidemiology* 2015;26(4):565-74.
30. Phillips DIW, Osmond C, Williams ML, et al. Air pollution in early life and adult mortality from chronic rheumatic heart disease. *International Journal of Epidemiology* 2016: Oct 23. pii: dyw249.
31. Cai Y, Zhang B, Ke W, et al. Associations of Short-Term and Long-Term Exposure to Ambient Air Pollutants With Hypertension: A Systematic Review and Meta-Analysis. *Hypertension* 2016;68(1):62-70.
32. Trimble CL, Genkinger JM, Burke AE, et al. Active and passive cigarette smoking and the risk of cervical neoplasia. *Obstet Gynecol* 2005;105(1):174-81.
33. Norman P, Boyle P, Rees P. Selective migration, health and deprivation: a longitudinal analysis. *Soc Sci Med* 2005;60(12):2755-71.
34. Strachan DP, Leon DA, Dodgeon B. Mortality from cardiovascular disease among interregional migrants in England and Wales. *BMJ* 1995;310(6977):423-7.
35. Osmond C, Barker DJ, Slattery JM. Risk of death from cardiovascular disease and chronic bronchitis determined by place of birth in England and Wales. *JEpidemiolCommunity Health* 1990;44(2):139-41.
36. Fox AJ, Goldblatt PO. Socioeconomic differences in mortality. *Population Trends* 1982;27:8-13.
37. Boyle P. Population geography: migration and inequalities in mortality and morbidity. *Progress in Human Geography* 2004;28(6):767-76.
38. Pooley CG, Turnbull J. Migration trends in British rural areas from the 18th to the 20th centuries. *IntJPopulGeogr* 1996;2(3):215-37. doi: 10.1002/(SICI)1099-1220(199609)2:3<215::AID-IJPG35>3.0.CO;2-N [doi]
39. Dennett AR. Understanding Internal Migration in Britain at the Start of the 21st Century. PhD Thesis, University of Leeds, 2010.
40. Champion T. Migration and British Cities in the 1990s. *National Institute Economic Review* 1999;170:60-76.
41. Evans GW, Kantrowitz E. Socioeconomic status and health: The potential role of environmental risk exposure. *Annu Rev Publ Health* 2002;23:303-31.
42. Kleinschmidt I, Hills M, Elliott P. Smoking behaviour can be predicted by neighbourhood deprivation measures. *J Epidemiol Community Health* 1995;49 Suppl 2:S72-7.

For peer review only

For peer review only - http://bmjopen.bmj.com/site/about/guidelines.xhtml

Table 1: Fuel consumption in England and Wales 1951/1952.

	Total annual consumption (tons x 1000)	Annual consumption per acre in the 342 LGDs (tons)	
		Median	Quartiles
Total Domestic	36,783	1.00	0.29,4.38
(Smokeless)	5,247	0.17	0.05,0.63
Total industrial	75,597	1.17	0.23,5.58
(Smokeless)	24,294	0.47	0.12,2.07
Carbonisation & briquetting	55,122	0.63	0.10,5.36
Electricity generation	33,304	0.07	0.00,1.85
Total	200,806	4.21	0.82,21.38

For peer review only - http://bmjopen.bmj.com/site/about/guidelines.xhtml

Table 2: The effect of fuel consumed in 1951/52, socioeconomic indicators derived from the 1951 census, and current microparticulate exposure ($PM_{2.5}$) on current causes of mortality in the 342 LGDs in England and Wales. ([1]ICD9: 460 to 519, ICD10: J00 to J99; [2]ICD9: 390 to 459 ICD10: I00 to I99. ICD9: [3]140-208, ICD10: C00 to C97)

Predictor	Hazard ratio per SD change			
	All Cause	Respiratory mortality[1]	Cardiovascular mortality[2]	Cancer mortality[3]
**Univariate analyses**				
Fuel consumed 1951/52				
Domestic (ex. smokeless)	1.124(1.123 to 1.126)	1.238(1.234 to 1.242)	1.138(1.136 to 1.140)	1.073(1.071 to 1.075)
Industrial (ex. smokeless)	1.129(1.128 to 1.131)	1.244(1.239 to 1.248)	1.152(1.150 to 1.154)	1.080(1.078 to 1.082)
Carbonization	1.120(1.119 to 1.122)	1.225(1.221 to 1.230)	1.135(1.133 to 1.137)	1.073(1.071 to 1.074)
Electricity generation	1.096(1.095 to 1.097)	1.181(1.177 to 1.185)	1.110(1.107 to 1.112)	1.054(1.052 to 1.056)
1951 census				
Social Class	1.117(1.115 to 1.118)	1.190(1.186 to 1.194)	1.144(1.142 to 1.146)	1.087(1.085 to 1.089)
Education	1.093(1.092 to 1.094)	1.154(1.150 to 1.158)	1.125(1.123 to 1.127)	1.064(1.063 to 1.066)
Crowding	1.106(1.105 to 1.108)	1.179(1.175 to 1.183)	1.126(1.124 to 1.128)	1.075(1.073 to 1.077)
Density	1.105(1.104 to 1.106)	1.206(1.202 to 1.211)	1.113(1.111 to 1.115)	1.059(1.057 to 1.061)
Unemployment	1.074(1.073 to 1.076)	1.113(1.109 to 1.117)	1.077(1.075 to 1.079)	1.055(1.053 to 1.057)
Current $PM_{2.5}$	1.027(1.026 to 1.028)	1.076(1.073 to 1.080)	1.028(1.027 to 1.030)	1.005(1.003 to 1.006)
**Multivariate analysis**				
Fuel consumed 1951/52				
Domestic (ex. smokeless)	1.127(1.123 to 1.131)	1.218(1.205 to 1.231)	1.123(1.117 to 1.130)	1.077(1.072 to 1.083)
Industrial (ex. smokeless)	1.001(0.998 to 1.003)	1.020(1.012 to 1.028)	1.012(1.008 to 1.016)	1.005(1.001 to 1.009)
Carbonization	1.020(1.018 to 1.022)	1.033(1.027 to 1.039)	1.020(1.016 to 1.023)	1.012(1.009 to 1.015)
Electricity generation	1.002(1.000 to 1.003)	1.000(0.995 to 1.005)	1.005(1.002 to 1.008)	0.995(0.992 to 0.997)
1951 census				
Social Class	1.066(1.064 to 1.068)	1.100 (1.093 to 1.107)	1.063(1.059 to 1.067)	1.057(1.054 to 1.061)
Education	1.015(1.014 to 1.017)	1.021(1.015 to 1.026)	1.039(1.036 to 1.042)	1.004(1.001 to 1.006)
Crowding	1.000(0.999 to 1.002)	0.994(0.990 to 0.999)	1.005(1.003 to 1.008)	1.003(1.001 to 1.005)
Unemployment	1.003(1.001 to 1.005)	0.999(0.993 to 1.004)	1.003(1.000 to 1.006)	1.004 (1.002 to1.007)
Current $PM_{2.5}$	0.946(0.944 to 0.947)	0.931(0.926 to 0.936)	0.940(0.937 to 0.942)	0.956(0.953 to 0.959)

For peer review only - http://bmjopen.bmj.com/site/about/guidelines.xhtml

Combined effects analysis of domestic consumption and density				
Unadjusted				
Sum (domestic + density)	1.112(1.111 to 1.113)	1.219(1.215 to 1.223)	1.122(1.120 to 1.124)	1.064(1.062 to 1.066)
Difference (domestic – density)	1.074(1.073 to 1.076)	1.117(1.113 to 1.121)	1.095(1.093 to 1.097)	1.052(1.051 to 1.054)
Adjusted*				
Sum (domestic + density)	1.114(1.110 to 1.118)	1.198(1.185 to 1.211)	1.106(1.100 to 1.113)	1.070(1.065 to 1.076)
Difference (domestic – density)	1.023(1.021 to 1.024)	1.038(1.033 to 1.042)	1.027(1.025 to 1.030)	1.012(1.010 to 1.014)

*Adjusted for social class, education, crowding, unemployment, industrial usage, fuel used in carbonization & electricity generation and current $PM_{2.5}$.

For peer review only - http://bmjopen.bmj.com/site/about/guidelines.xhtml

Table 3: Association between domestic fuel consumption (ex. smokeless fuel) and major causes of non-cancer mortality among men and women aged 35-74 in the 342 areas.

	Hazard ratio per SD increase in domestic fuel consumption			
	Unadjusted (95%CI)		Adjusted* (95% CI)	
Respiratory				
Chronic obstructive pulmonary disease	1.253	1.245 to 1.260	1.299	1.285 to 1.314
Asthma	1.199	1.176 to 1.223	1.226	1.183 to 1.270
Pneumonia	1.262	1.254 to 1.269	1.273	1.259 to 1.287
Tuberculosis	1.734	1.676 to 1.793	1.599	1.500 to 1.706
Cardiovascular				
Ischaemic heart disease	1.141	1.138 to 1.143	1.173	1.168 to 1.178
Rheumatic heart disease	1.209	1.184 to 1.234	1.208	1.164 to 1.254
Stroke	1.137	1.133 to1.141	1.173	1.165 to 1.182
Hypertension	1.204	1.196 to 1.212	1.171	1.156 to 1.185

*Adjusted for social class, education, overcrowding, unemployment and current $PM_{2.5}$.

For peer review only - http://bmjopen.bmj.com/site/about/guidelines.xhtml

Table 4: Association between domestic fuel consumption (ex. smokeless fuel) and cancer mortality in the 342 areas.

Site	Hazard ratio per SD increase in domestic fuel consumption			
	Unadjusted (95%CI)		Adjusted* (95% CI)	
Lip, oral cavity and pharynx	1.220	1.204 to 1.236	1.283	1.252 to 1.314
Oesophagus	1.038	1.030 to 1.046	1.131	1.116 to 1.147
Stomach	1.171	1.161 to 1.182	1.166	1.147 to 1.185
Colon	0.996	0.989 to 1.002	1.041	1.028 to 1.053
Rectosigmoid junction, rectum and anus	1.073	1.063 to 1.082	1.085	1.068 to 1.103
Liver, gall bladder & bile ducts	1.193	1.179 to 1.206	1.174	1.151 to 1.199
Pancreas	1.022	1.014 to 1.030	1.028	1.014 to 1.042
Larynx	1.347	1.318 to 1.376	1.323	1.272 to 1.376
Trachea, bronchus and lung	1.195	1.190 to 1.199	1.219	1.211 to 1.226
Malignant melanoma	0.885	0.873 to 0.898	0.927	0.904 to 0.950
Breast	0.990	0.984 to 0.995	0.986	0.976 to 0.996
cervix	1.155	1.135 to 1.175	1.229	1.191 to 1.269
Uterus	1.032	1.015 to 1.049	0.999	0.970 to 1.029
Ovary	0.968	0.959 to 0.976	0.992	0.976 to 1.008
Prostate	0.989	0.981 to 0.998	0.995	0.980 to 1.010
Kidney	1.000	0.989 to1.011	1.058	1.037 to1.078
Bladder	1.056	1.044 to 1.068	1.066	1.045 to 1.087
Brain , eye & CNS	0.952	0.943 to 0.961	0.974	0.957 to 0.990
Hodgkin's disease	1.030	0.994 to 1.067	1.007	0.945 to 1.074
Non-Hodgkin's lymphoma	1.024	1.014 to 1.034	1.011	0.993 to 1.029
Multiple myeloma	0.999	0.986 to 1.013	0.983	0.959 to 1.007
Leukaemia	1.008	0.998 to 1.019	1.009	0.990 to 1.029

*Adjusted for social class, education, overcrowding, unemployment and current $PM_{2.5}$.

For peer review only

Appendix 1

Cause of death	ICD 9 code	ICD 10 code	Number of deaths
**Cardiovascular disease**			
Ischaemic heart disease	410-414	I20-I25	710,979
Rheumatic heart disease	393-398	I05-I09	9,639
Cerebrovascular disease	430-438	I60-I69	264,853
Hypertension	401-405	I10-I15	84,208
**Respiratory disease**			
Chronic bronchitis and emphysema	490-492,496	J40-J44	105,690
Asthma	493	J45-J46	10,486
Pneumonia	480-486	J12-J18	107,848
Tuberculosis	010-018,137	A15-A19,B90	3,784
**Cancers**			
Lip, oral cavity and pharynx	140-149	C00-C14	22,992
Oesophagus	150	C15	65,854
Stomach	151	C16	49,634
Colon	153	C18	85,176
Rectosigmoid junction, rectum & anus	154	C19-C21	49,649
Liver, gall bladder and bile ducts	155,156	C22 – C24	31,240
Pancreas	157	C25	66,273
Larynx	161	C32	8,909
Trachea, bronchus & lung	162	C33,c34	335,217
Malignant melanoma	172	C43	20,045
Breast	174-175	C50	128,494
Cervix	180	C53	12,947
Uterus	179,182	C54-C55	14,315
Ovary	183	C56	47,895
Prostate	185	C61	54,521
Kidney	189	C64	33,232
Bladder	188	C67	32,353
Brain, Eye & CNS	191-192	C69-C72	43,811
Hodgkin's disease	201	C81	3,096
Non-Hodgkin's lymphoma	200,202	C82-C85	40,783
Multiple myeloma	203	C90	21,510
Leukaemia	204-208	C91-C95	33,526

For peer review only

Figure 1: Domestic fuel consumption in England and Wales, 1951-52.

Figure 2: Association between quartiles of domestic fuel consumption (ex smokeless) and both all-cause and the major causes of mortality in men and women aged 35-74 in the 342 LGDs. (●unadjusted, ○ adjusted for social class, education, crowding, unemployment and current $PM_{2.5}$; errors are 95% CI)

Figure 3: Hazard ratio of all-cause and the major causes of mortality per SD change in domestic consumption (ex. smokeless) according to birth year. Adjusted for social class, education, crowding, unemployment and current $PM_{2.5.}$

For peer review only

Figure 1

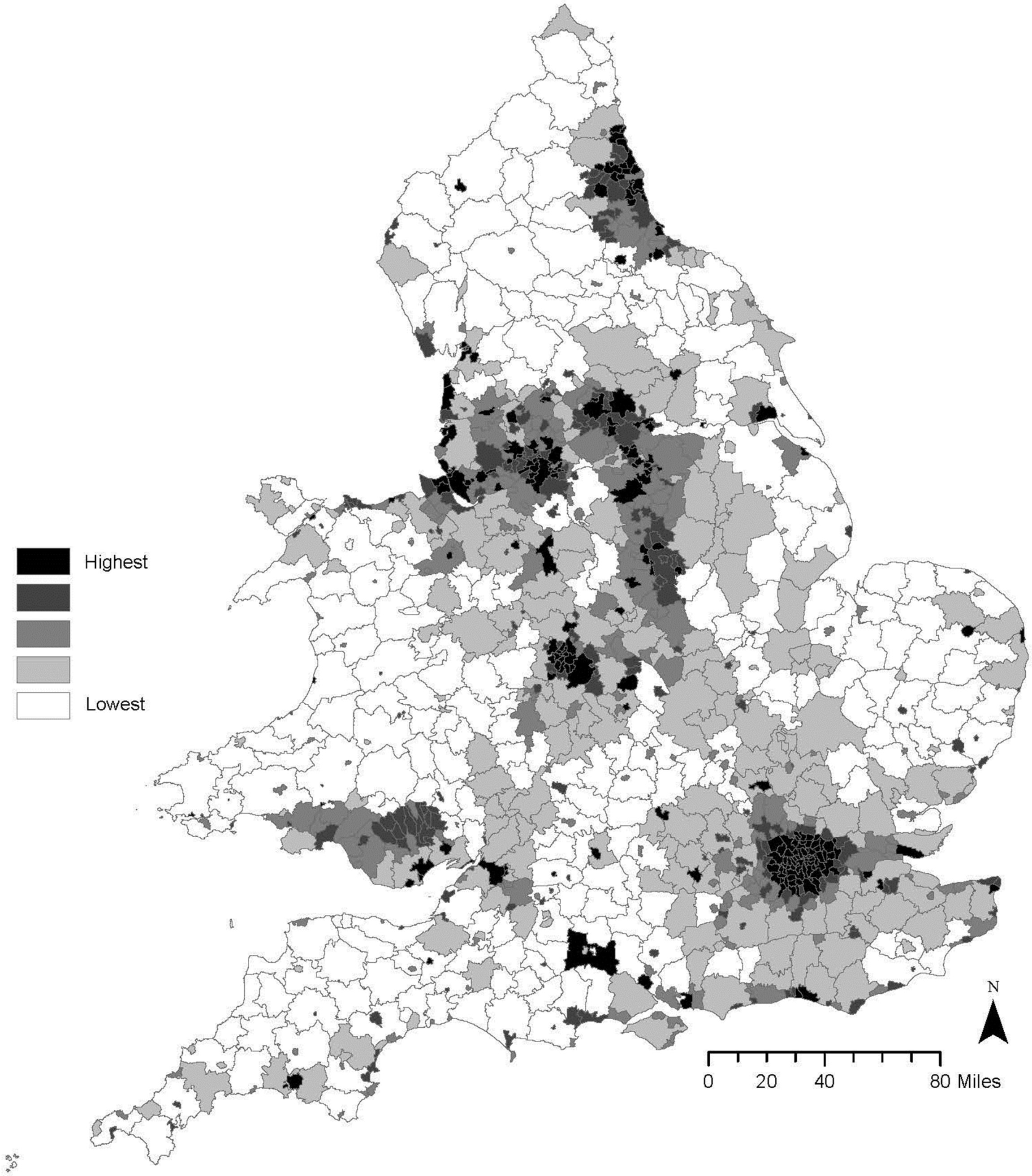

For peer review only - http://bmjopen.bmj.com/site/about/guidelines.xhtml

Figure 2

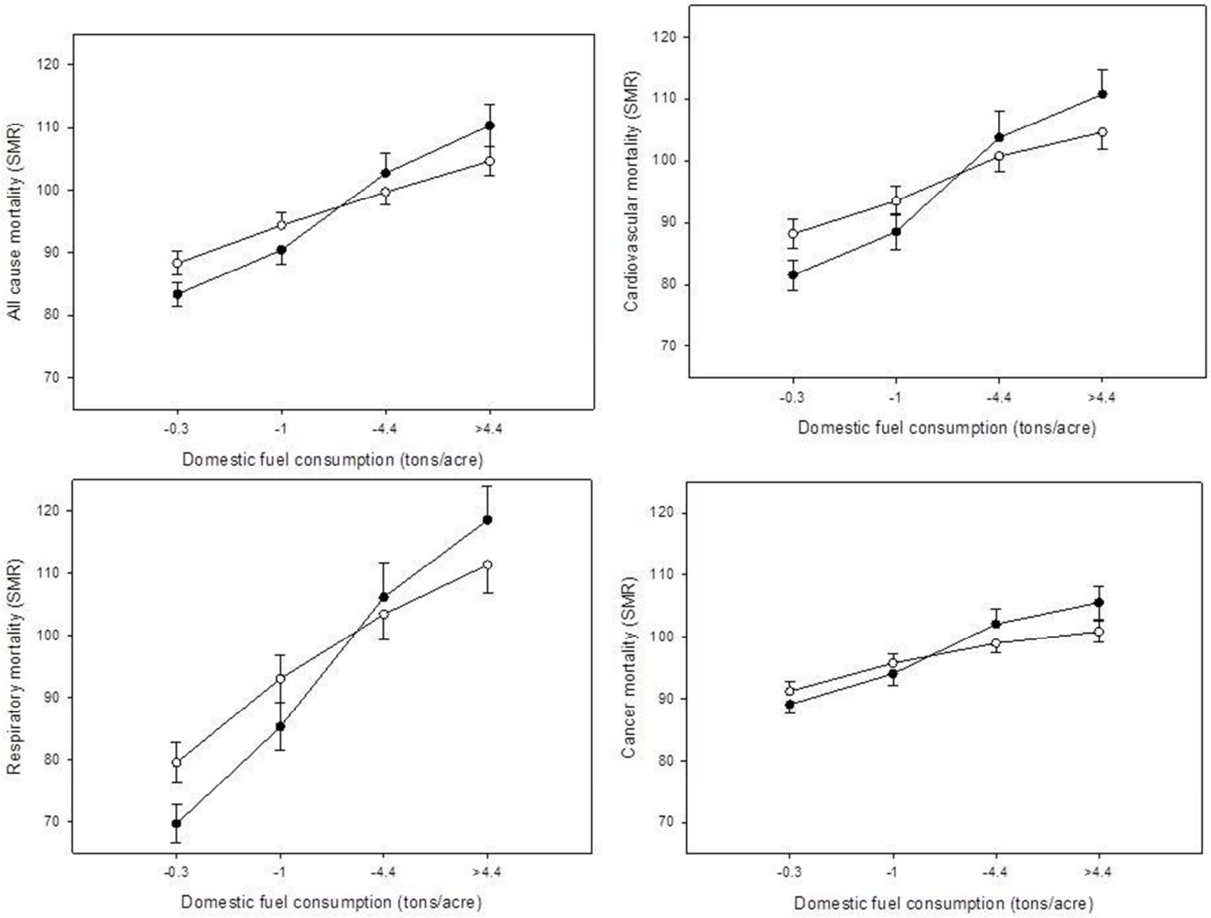

For peer review only - http://bmjopen.bmj.com/site/about/guidelines.xhtml

Figure 3

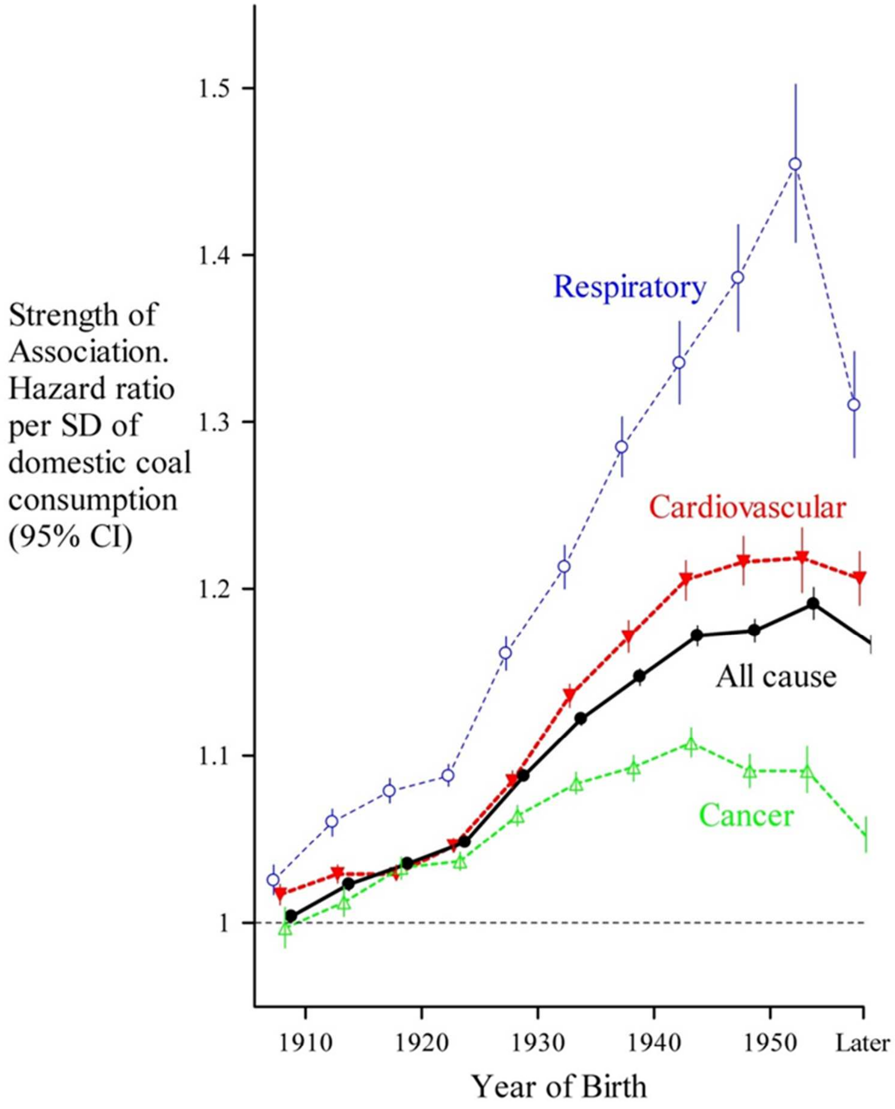

For peer review only - http://bmjopen.bmj.com/site/about/guidelines.xhtml

Footnotes

Contributors: DIWP discovered the dataset on coal consumption in 1951/2 and together with CO conceived the study. HS and PA advised on the use of the geographical data and carried out the redistricting analysis. DIWP, CO and AJ were responsible for the statistical analysis. The manuscript was written by DIWP, CO, AJ, HS and SH. All authors participated in a critical revision of the text and approved the final manuscript. DIWP is responsible for the overall content as the corresponding author.

Funding: The work was supported by the Medical Research Council.

Competing interests: None declared.

Ethics approval: As the study was based on routinely collected mortality data no ethics approval was necessary.

Data sharing: Under the MRC's "Policy and Guidance on Sharing of Research Data from Population and Patient Studies" the dataset is available upon request to diwp@mrc.soton.ac.uk.

For peer review only

STROBE 2007 (v4) checklist of items to be included in reports of observational studies in epidemiology*
Checklist for cohort, case-control, and cross-sectional studies (combined)

Section/Topic	Item #	Recommendation	Reported on page #
**Title and abstract**	1	(*a*) Indicate the study's design with a commonly used term in the title or the abstract	2
		(*b*) Provide in the abstract an informative and balanced summary of what was done and what was found	2
**Introduction**			
Background/rationale	2	Explain the scientific background and rationale for the investigation being reported	4
Objectives	3	State specific objectives, including any pre-specified hypotheses	4
**Methods**			
Study design	4	Present key elements of study design early in the paper	5-6
Setting	5	Describe the setting, locations, and relevant dates, including periods of recruitment, exposure, follow-up, and data collection	5-6
Participants	6	(*a*) *Cohort study*—Give the eligibility criteria, and the sources and methods of selection of participants. Describe methods of follow-up<br>*Case-control study*—Give the eligibility criteria, and the sources and methods of case ascertainment and control selection. Give the rationale for the choice of cases and controls<br>*Cross-sectional study*—Give the eligibility criteria, and the sources and methods of selection of participants	5-6
		(*b*) *Cohort study*—For matched studies, give matching criteria and number of exposed and unexposed<br>*Case-control study*—For matched studies, give matching criteria and the number of controls per case	N/a
Variables	7	Clearly define all outcomes, exposures, predictors, potential confounders, and effect modifiers. Give diagnostic criteria, if applicable	5-6, 20 (appendix)
Data sources/ measurement	8*	For each variable of interest, give sources of data and details of methods of assessment (measurement). Describe comparability of assessment methods if there is more than one group	*5-6*
Bias	9	Describe any efforts to address potential sources of bias	6
Study size	10	Explain how the study size was arrived at	N/A
Quantitative variables	11	Explain how quantitative variables were handled in the analyses. If applicable, describe which groupings were chosen and why	6
Statistical methods	12	(*a*) Describe all statistical methods, including those used to control for confounding	6
		(*b*) Describe any methods used to examine subgroups and interactions	N/A
		(*c*) Explain how missing data were addressed	N/A

For peer review only

		(*d*) *Cohort study*—If applicable, explain how loss to follow-up was addressed<br>*Case-control study*—If applicable, explain how matching of cases and controls was addressed<br>*Cross-sectional study*—If applicable, describe analytical methods taking account of sampling strategy	N/A
		(*e*) Describe any sensitivity analyses	11
**Results**			
Participants	13*	(a) Report numbers of individuals at each stage of study—eg numbers potentially eligible, examined for eligibility, confirmed eligible, included in the study, completing follow-up, and analysed	7
		(b) Give reasons for non-participation at each stage	N/A
		(c) Consider use of a flow diagram	N/A
Descriptive data	14*	(a) Give characteristics of study participants (eg demographic, clinical, social) and information on exposures and potential confounders	15 (Table 1)
		(b) Indicate number of participants with missing data for each variable of interest	N/A
		(c) *Cohort study*—Summarise follow-up time (eg, average and total amount)	5
Outcome data	15*	*Cohort study*—Report numbers of outcome events or summary measures over time	7
		Case-control study—Report numbers in each exposure category, or summary measures of exposure	N/A
		Cross-sectional study—Report numbers of outcome events or summary measures	N/A
Main results	16	(*a*) Give unadjusted estimates and, if applicable, confounder-adjusted estimates and their precision (eg, 95% confidence interval). Make clear which confounders were adjusted for and why they were included	16,17,18,19 (Tables 2-4)
		(*b*) Report category boundaries when continuous variables were categorized	N/A
		(*c*) If relevant, consider translating estimates of relative risk into absolute risk for a meaningful time period	N/A
Other analyses	17	Report other analyses done—eg analyses of subgroups and interactions, and sensitivity analyses	N/A
**Discussion**			
Key results	18	Summarise key results with reference to study objectives	9
Limitations	19	Discuss limitations of the study, taking into account sources of potential bias or imprecision. Discuss both direction and magnitude of any potential bias	11,12
Interpretation	20	Give a cautious overall interpretation of results considering objectives, limitations, multiplicity of analyses, results from similar studies, and other relevant evidence	12
Generalisability	21	Discuss the generalisability (external validity) of the study results	12
**Other information**			
Funding	22	Give the source of funding and the role of the funders for the present study and, if applicable, for the original study on which the present article is based	25

*Give information separately for cases and controls in case-control studies and, if applicable, for exposed and unexposed groups in cohort and cross-sectional studies.

For peer review only

Note: An Explanation and Elaboration article discusses each checklist item and gives methodological background and published examples of transparent reporting. The STROBE checklist is best used in conjunction with this article (freely available on the Web sites of PLoS Medicine at http://www.plosmedicine.org/, Annals of Internal Medicine at http://www.annals.org/, and Epidemiology at http://www.epidem.com/). Information on the STROBE Initiative is available at www.strobe-statement.org.

For peer review only

BMJ Open

Evaluating the early life effects of air pollution: geographical correlations between coal consumption in 1951/2 and current mortality in England and Wales.

Journal:	*BMJ Open*
Manuscript ID	bmjopen-2017-018231.R1
Article Type:	Research
Date Submitted by the Author:	31-Oct-2017
Complete List of Authors:	Phillips, David; Medical Research Council<br>Osmond, Clive; Medical Research Council, Epidemiology Resource Centre<br>Southall, Humphrey; University of Portsmouth, Dept of Geography<br>Aucott, Paula; University of Portsmouth, Dept of Geography<br>Jones, Alexander; University of Oxford, Dept of Paediatrics<br>Holgate, Stephen; Southampton General Hospital, Medicine and Therapeutics
**Primary Subject Heading**:	Epidemiology
Secondary Subject Heading:	Occupational and environmental medicine, Public health
Keywords:	air pollution, Mortality, Developmental origins of health and diseasee

SCHOLARONE™ Manuscripts

For peer review only

Evaluating the early life effects of air pollution: geographical correlations between coal consumption in 1951/2 and current mortality in England and Wales.

David IW Phillips, professor[1];Clive Osmond, professor of medical statistics[1]; Humphrey Southall, professor in historical geography[2]; Paula Aucott, senior research associate; Alexander Jones, paediatric cardiologist[3] ;Stephen T Holgate, professor of immunopharmacology.[4]

From

[1]The Medical Research Council's Lifecourse Epidemiology Unit, University of Southampton, Southampton, SO16 6YD. UK. ; [2]Department of Geography, University of Portsmouth, Portsmouth, PO1 2UP, UK. ; [3]Department of Paediatrics, University of Oxford, Oxford, OX3 9DU, UK. [4]Department of Medicine, University of Southampton, Southampton, SO16 6YD, UK.

Correspondence: DIW Phillips, MRC Lifecourse Epidemiology Unit, Southampton General Hospital, Southampton, UK.

Email: diwp@mrc.soton.ac.uk

Tel +44 (0) 23 80777624

FAX + 44 (0) 23 80704021

Short title: Long term effects of coal consumption

For peer review only - http://bmjopen.bmj.com/site/about/guidelines.xhtml

ABSTRACT

Objective

The short-term effects of air pollution are well-documented, but less is known about whether early life pollutant exposure has long-term adverse effects.

Design

Geographical study

Setting

Local authority areas in England and Wales

Exposures

Routinely collected data on the use of coal and related solid fuels in 1951-2 were used as an index of air pollution from domestic and industrial sources in different geographical areas of England and Wales.

Main outcome measures

We evaluated the relationship between these data and both all-cause and disease-specific mortality in the geographically equivalent 342 current local authority areas in 1993-2012.

Results

Of all the sources of solid fuel, domestic usage had the most powerful effect on mortality. There were strong relationships between domestic fuel use and all-cause mortality (relative risk per SD increase in fuel use 1.124, 95% CI = 1.123-1.126), and with respiratory (1.238, 1.234-1.242), cardiovascular (1.138, 1.136-1.140), and cancer mortality (1.073, 1.071-1.075). These effects persisted after adjustment for socioeconomic indicators in 1951, current socioeconomic indicators and current pollution levels.

Conclusion

Coal was the major cause of pollution in the UK until the Clean Air Act of 1956 led to a rapid decline in consumption. Although based on geographical correlations, these data raise the possibility that coal-based pollution, experienced over 60 years ago in early life, affects human health now by increasing mortality from a wide variety of diseases.

For peer review only - http://bmjopen.bmj.com/site/about/guidelines.xhtml

Strengths and limitations of this study

•The first to evaluate the very long-term (60 year) mortality resulting from air pollution experienced in early life.

•Use of national mortality data with virtually complete ascertainment and a large number of deaths (> 3.5 million).

•Coal consumption data provide an integral of air pollution over a 12 month period.

•The analysis also allows for current pollution levels

•The study depends on the use of geographical correlations together with census-derived measures of socio-economic status; individual-level data are not available.

For peer review only

For peer review only - http://bmjopen.bmj.com/site/about/guidelines.xhtml

INTRODUCTION

Air pollution has detrimental effects on health at all ages[1] but little is known about the long-term impact of early life exposure to air pollution, which continues to be an important worldwide problem as open fires and the consequent exposure of mothers and young children to pollutants is widespread in many low- and middle-income countries.[2-4] There is accumulating evidence that the young are especially vulnerable. Developing tissues and organs are particularly susceptible due to their high cell proliferation rates in prenatal life. Postnatally, infants and young children also have increased vulnerability due to immature detoxification systems, high infection rates and patterns of behaviour that increase their pollutant exposure.[2] Pregnant mothers exposed to air pollution are more likely to give birth prematurely and have offspring with intrauterine growth retardation and poor growth in infancy.[5 6] Such outcomes are linked with long-term adverse health effects, including increased risks of respiratory and cardiovascular disease.[7]

In common with many other countries, the UK had high levels of air pollution in the past.[8] Most of this was due to coal use in industry and as a domestic fuel, which gave rise to the "great stinking fogs" that previously characterised London and other major cities.[8] The London smog of 1952 killed an estimated 4,000 people[9] and led to the first UK Clean Air Act being passed in 1956. Following the 1952 smog, a national survey of smoke and SO_2 concentrations was set up during the 1960s to monitor the progress of pollution control following the Act. From around 1960, there was a progressive reduction in black smoke and SO_2 emissions, with nitrogen oxides, ozone and microparticulates from transport becoming the dominant forms of air pollution.[10]

Little is known about the long-term health effects of burning such vast quantities of coal, which still exceeded 200 million tons per year in England and Wales at the middle of the 20th century.[11] Studies published in the 1950s and 1960s suggested that there were strong cross-sectional correlations of the amounts of coal used in domestic fires with childhood respiratory disease [12] and with many major causes of adult mortality[13 14] but it is not known if there were lasting effects. The present study is based on a dataset compiled by the Ministry of Fuel and Power that recorded the quantities of different types of solid fuel burnt annually between May 1951 and May 1952 in 1,145 areas of England and Wales. The extent to which exposure to the resulting pollutants may have influenced present day health was assessed by relating these data to mortality rates in the same areas 50-60 years later.

For peer review only - http://bmjopen.bmj.com/site/about/guidelines.xhtml

METHODS

Shortages of coal following the Second World War led to the continuation of a system of rationing and distribution that had been initiated in 1939. This system was controlled by local fuel overseers (LFOs) who were appointed for each local government district (LGD) or group of districts. The Ministry of Fuel and Power published estimates of the quantities of different types of solid fuel burnt annually in each of the 1,145 LFO areas between May 1951 and May 1952 in England and Wales – data were only published for these years.[15] Domestic supplies included coal, the amounts used in each LFO area based on the number of registered premises, and other solid fuels including coke, briquettes, ovoids and similar patent fuels where usage was based on merchant's disposals. The annual household coal allowance was higher in the North (2,540 kg) than the South (1,727 kg). Miners' concessionary coal was also included. Industrial fuels were coal and coke and there were separate listings of the quantities of fuel used in electricity generation and for carbonization or briquetting plants. The amounts of smokeless fuel used were recorded separately.

The Office for National Statistics (ONS) provided extracts from all death certificates in England and Wales for each of the current LGDs during 1993 to 2012. This is the period covered by the ninth and tenth revisions of the International Classification of Disease (ICD). The ICD codes used to define causes of death are given in Appendix 1. Mortality rates were calculated using population estimates provided by the ONS for each LGD. The main technical problem was to relate the historical 1,145 LFO areas to the current 342 LGDs, following successive local government reorganisations in 1974, the 1990s, and 2009, together with numerous individual boundary changes. Detailed digital boundaries for the LGDs of England and Wales used in the 1951 census have already been constructed as part of the Great Britain Historical Geographical Information System (GIS).[16 17] As the LFO areas either exactly corresponded to LGDs or were defined as aggregates of them, constructing digital boundary data for LFO areas was straightforward. However, modern LGDs are not necessarily simple aggregates of the LFO areas, so the first stage of the redistricting procedure was to re-allocate the fuel consumption data to the 12,504 Civil Parishes of 1951. Digital boundaries already existed for the parishes, and the re-allocation was based on what proportion each parish's total population, as listed by the 1951 census, was of the overall population of the containing LFO area. The fuel data were then re-allocated to the modern areas by digitally overlaying the two sets of boundaries: whatever proportion of a 1951 parish's area fell within a modern district, that proportion of the parish's fuel consumption was assigned to the district.

For peer review only - http://bmjopen.bmj.com/site/about/guidelines.xhtml

Historically, air pollution was worse in predominantly industrial towns with a high population density; these towns also tended to have large populations of factory workers who left school early and worked in low status occupations.[13] These are important potential confounders in a study of air pollution and disease, as poverty and overcrowding are known to be associated with many of the major causes of mortality. To control for these we used a series of variables extracted from the published reports of the 1951 census: male unemployment; indices of social class; overcrowding, assessed as the proportion of households with more than one person per room; density, the number of people per acre; and educational achievement, indicated by the proportion of employed men in each area who left school before the age of 15 years. Social class was based on the Registrar General's occupation-based system that categorises people into five ordinal groups (I) Professional occupations, (II) Managerial and Technical occupations, (III) Skilled occupations, (IV) Partly-skilled occupations and (V) Unskilled occupations which were combined into a weighted score.[18] Parallel data were obtained from the 2001 census: unemployment rate, social grade, proportion of households with >1 person/room, persons/hectare, and proportion with no qualifications. We also allowed for the impact of current levels of air pollution, which were obtained from published data reporting the modelled annual average concentrations of fine particulate matter ($PM_{2.5}$) in each of the 342 LGDs.[19]

Statistical methods

We studied deaths in people aged between 35 and 74 years, as this was the approximate age range of the generation who were in their first decade of life in 1951-52, and excluded older age groups where the cause of death was less likely to have been ascertained accurately.[20] We calculated the number of deaths expected for each five-year age and sex group within the LGDs by multiplying the number of people in each group by the national age- and sex-specific death rates in 1993-2012. We expressed the number of observed deaths in each area as a percentage of the expected deaths for that group, i.e. as standardised mortality ratios (SMRs). We used Poisson regression to model SMRs, expressing the effect of fuels and other variables on mortality in terms of the hazard ratio (or relative risk) per SD change in the explanatory variable. This weights the analyses appropriately to allow for variation in population size across LGDs. A Fisher-Yates transform was used to standardise pollution and socioeconomic variables. This yielded variables with zero means, unit standard deviations (SDs) and symmetrical distributions, allowing direct comparison of the strength of associations with these variables.

For peer review only - http://bmjopen.bmj.com/site/about/guidelines.xhtml

RESULTS

There were a total of 3,535,136 deaths in England and Wales during the 20-year period from 1993 to 2012 in the 35- to 74-yr age-group. Mortality rates (SMR) in the 342 LGDs ranged from 64.7 in East Dorset to 153.1 in Manchester. Table 1 shows the different uses of solid fuel during 1951/2 in England and Wales. Of the over 200 million tons distributed, 37.6% was used in industrial processes, 16.6% in electricity generation and 27.5% in carbonization or briquetting processes. Only 18.3% was used domestically. Smokeless fuel accounted for approximately 32.1% of industrial and 14.3% of domestic consumption. Figure 1 is a map of England and Wales showing the quintiles of domestic fuel consumed per acre. This was generally low in rural areas but was greatest in London and in the former industrial areas of the UK (South Wales, the Midlands, Lancashire, the West Riding of Yorkshire and Tyneside). The mean current $PM_{2.5}$ concentrations in the 342 areas was 9.48 (SD 1.67) $\mu g/m^3$ and ranged from 5.5 in Gwynedd (Wales) to 14.9 $\mu g/m^3$ in the City of Westminster, London.

Table 2 shows the relationship between the various sources of fuel burnt in 1951/2 (excluding smokeless fuels) and subsequent all-cause mortality or mortality due to respiratory, cardiovascular disease and cancer in the 342 areas. The influence of socioeconomic indicators (social class, education, crowding, density and unemployment) are also shown. In univariate analyses, all variables were significantly associated with both all-cause and cause-specific mortality rates although the effects of domestic and industrial consumption tended to be most strongly related to the mortality outcomes. Multivariate analyses were carried out to evaluate the relative effects of fuel usage and the major confounding variables derived from the 1951 census. This analysis shows that domestic consumption was strongly and significantly associated with both all-cause and cause-specific mortality rates, an effect which was markedly stronger than the other sources of fuel. There was a very high correlation (r=0.97) between domestic consumption and population density, resulting in unacceptable collinearity in regression analyses that contained both variables. This occurred because both had the same denominator, the area of the LGD. To allow the influence of both of these factors to be modelled statistically, we created two further variables: the sum of and the difference between domestic use and density. These variables were uncorrelated (r=0.0). These were entered into the regression models in Table 2, which shows both the unadjusted coefficients and the coefficients adjusted for all other variables (Table 2 lower panel). The positive regression coefficients for the sum variable show that the joint effects of domestic use and density on all four causes of mortality were powerful. However, associations with the difference variable suggest that the influence of domestic use was stronger than that of density.

For peer review only - http://bmjopen.bmj.com/site/about/guidelines.xhtml

For peer review only

Table 3 shows the relationship between current socioeconomic indicators or microparticulate air pollution and all-cause or cause-specific mortality. The lower section of table 3 shows that the effect of domestic coal usage is attenuated by successive adjustments for socioeconomic indicators from the 1951 census, for indicators from the 2001 census together with particulate exposure and for both of these sets of factors. However, in all cases the association with coal usage remained strong and statistically significant.

Smokeless fuels represented a small proportion of the total fuels used and contributed little to the mortality risks. The effects of the potential confounders were tested singly and in combination. There was no evidence of non-linear relationships. Figure 2 shows the associations between quartiles of domestic fuel consumption and all-cause mortality or the major mortality categories. These associations were progressive and remained strong and statistically significant for each cause group after adjustment for social class, education, crowding, unemployment in 1951, and for current socioeconomic indicators and $PM_{2.5}$ concentrations. They were present in both genders and were similar during the ICD 9 and ICD 10 periods.

Figure 3 shows the strength of the geographical correlations between domestic consumption and current mortality according to successive birth cohorts. For all-cause and respiratory mortality, the strongest associations were observed among those born in 1952/3 suggesting that exposure around the time of birth had the greatest effect on mortality. For cardiovascular and cancer mortality, the peak associations were more blunted, suggesting that exposure over a wider age-range was associated with subsequent mortality.

Tables 4 & 5 show the relationship between domestic fuel consumption and specific causes of mortality. As before, the data are shown before and after adjustment for past and current socioeconomic indicators and current $PM_{2.5}$ levels. The respiratory conditions responsible for most deaths, chronic obstructive pulmonary disease and pneumonia, were strongly and consistently associated with domestic fuel consumption while associations with asthma mortality were somewhat weaker. However, the largest relative risks were with tuberculosis. While all four major causes of cardiovascular mortality were significantly associated with domestic consumption, the strongest relationship was with rheumatic heart disease.

The data on associations of domestic fuel use with mortality from specific cancers were striking (Table 5). There were strong relationships with epithelial cancers; lip, oral cavity and pharynx (adjusted hazard ratio, 1.207); laryngeal cancers (1.263) and lung (1.126). The upper gastrointestinal cancers; oesophagus (1.088), stomach (1.117) and liver (1.053), were more strongly associated with domestic pollution than the lower gastrointestinal cancers. In contrast, the major urinary, reproductive and haematological

For peer review only - http://bmjopen.bmj.com/site/about/guidelines.xhtml

cancers showed no increase in risk, with the exception of cervical cancer (1.131). Some cancers, for example melanoma and brain, were associated with a somewhat reduced relative risk.

For peer review only

For peer review only - http://bmjopen.bmj.com/site/about/guidelines.xhtml

DISCUSSION

Areas of the UK that had high domestic consumption of coal and related non-smokeless solid fuels in 1951/52 now have raised mortality from a wide variety of causes, including cardiovascular and respiratory diseases and certain cancers. The correlations are strong, statistically significant and independent of all available variables that might be considered major confounders, such as social class, level of education, overcrowding, unemployment and density assessed at birth and currently. They were also independent of current levels of air pollution as indicated by concentrations of microparticulate pollution ($PM_{2.5}$).

Very few comparable data exist in the literature, particularly with such a long follow-up period. However, our findings accord with a body of evidence that early life exposure to air pollution has detrimental long-term health effects.[14 21] In particular, two recent studies demonstrated important findings. In the first, children exposed to the great London Smog of 1952 were found to have greater risk of asthma in adulthood, compared to unexposed children.[22] In the second, which was based on the ONS longitudinal study, air pollution concentrations at the site of residence, assessed every 10 years from 1971, were associated with increased total, respiratory and cardiovascular mortality.[23] Our conclusions also accord with historical studies of coal use and mortality in Britain, which estimated that each SD increase in coal use raised mortality by 5-15% in infants and 5% in adults.[24]

Our study necessarily depended on coal consumption as an index of domestic air pollution. We contend that is very reasonable to assume that the quantity of coal burnt relates to the amount of pollution emitted. The consumption measures in our study were shown to correlate with subsequent measurements of pollution in the UK,[12] and this approach is also validated by a number of studies carried out over the last 60 years, showing correlations with diverse health outcomes including reduced early growth,[5] chronic bronchitis and lung cancer.[12-14] In the post-war years, economic necessity drove the use of low-grade, bituminous domestic coal while better-quality "hard" coals were exported. A typical, inefficient domestic grate burning low-grade coal would have produced lots of smoke, rich in a wide variety of potentially toxic compounds, including heavy metals, sulphur and complex mixtures of aliphatic and aromatic hydrocarbons.[25] Confirmation of this can be found in the lung tissue from autopsies of people exposed to the 1952 London smog, which was shown to contain both ultrafine carbonaceous and metal particulate matter.[26] Children would have been exposed both in the home and in their local environment although it is not clear from our data which of these would have been the most important source. However, our study suggests that it was domestic fuel consumption that had the greatest long-term adverse health effects, with industrial pollution or pollution from power stations or other sources causing relatively smaller effects (Table 2). The probable explanation for this is that most industrial pollution

For peer review only - http://bmjopen.bmj.com/site/about/guidelines.xhtml

was vented through tall chimneys, decreasing ground-level pollution in approximate proportion to the inverse square of chimney height.[27]

Current levels of pollution in the 342 local areas were based on ambient levels of fine particulate matter ($PM_{2.5}$) because large cohort studies have shown that this measure correlates best with long-term health effects of pollution.[28-30] The effects of $PM_{2.5}$ on current mortality (Table 2) in the present study are consistent with previously published estimates.[19]

Our data do not allow determination of the age at which children were most vulnerable to the effects of pollution although the cohort-based analysis (Figure 3) suggests that exposures around the time of birth or infancy are likely to have been most important especially for all-cause and respiratory mortality. Although the consumption data come from a short time-period between 1951 and 1952, they can be considered a reasonable reflection of smoke emission over many years. UK coal production was fairly constant during the late 1940s, declined a little during the 1950s and only fell dramatically after 1960.[11] We can also only speculate on the mechanism by which air pollution arising from coal consumption, predominantly in a domestic setting, might increase the risk of mortality. There may have been direct mutagenic effects, especially in relation to the increased cancer risk. Another possibility is that the pollutants triggered mechanisms that selected alternative developmental pathways in the young, perhaps through epigenetic modification of gene expression.[31]

Elevated relative risks were observed for respiratory diseases, especially COPD, asthma and pneumonia, (Table 4) which is consistent with past evidence that black smoke exposure is a risk factor for these conditions.[13 14 21] The strongest association we found, however, was with tuberculosis, which is less supported by existing literature. A recent systematic review and meta-analysis concluded that the evidence for such an association was inconclusive but it was reliant on case-control and cross-sectional studies.[32] The findings in the present study raise the possibility that early exposure to air pollution could be a critical factor in determining susceptibility to tuberculosis although other factors such as population density cannot be completely excluded. Cardiovascular disease was also found to be strongly associated with domestic fuel consumption, which accords with earlier UK findings,[14 21] the Harvard Six Cities study,[29] and a multicentre study of European Cohorts.[33] The strength of the association was similar with ischaemic heart disease and stroke. The association with rheumatic heart disease was strong and confirms the findings of a previous study based on a subset of large Country Boroughs in England and Wales, and further supports the suggestion that an increase in susceptibility to infectious diseases is linked with exposure to air pollution in infancy or early childhood.[34] An additional finding was a strong association with hypertension, which is supported by a meta-analysis that showed both short-term and long-term exposure to some air pollutants increases the risk of hypertension.[35] The data on cancers (Table 5) show wide variation in the level of associated risk. They show that the strongest associations

For peer review only - http://bmjopen.bmj.com/site/about/guidelines.xhtml

are observed with epithelial cancers of the respiratory system (lip, oral cavity and pharynx; larynx; trachea and lung) and the upper gastrointestinal system (stomach and liver). The major reproductive, urinary and haematological cancers were not positively associated with domestic pollution with the exception of cervical cancer, for which there is evidence that cigarette smoking is an important co-factor.[36] Malignant melanoma had a lower relative risk in the areas with high consumption possibly because of reduced sunlight exposure. Some cancers showed small reductions in relative risk, for example brain cancer, and although the reasons are not clear, this may be due to residual confounding, which is evident because of the very large numbers of deaths and high sensitivity of this analysis.

There are a number of potential limitations to our study. An inevitable issue with evaluating the association of early exposures with causes of late-life mortality is the long latency between, in this case, the measurements of coal consumption and the outcomes. It could be argued that internal migration might have weakened the associations that we observed as selective migration has been proposed as an explanation for geographical variations in mortality. This is supported by some studies[37] but not others.[38 39] Migration is complex as, while migrants are generally healthier, those moving short distances tend to have higher mortality than those moving long distances.[40 41] Although we do not have migration data, most migration during the time period of this study occurred over short distances, with only a small minority migrating significant distances.[42] A more recent study of migration based on the 2001 census, while documenting the complex age and social class influences on migration, suggested that the declining industrial areas of South Wales, Yorkshire, Greater Manchester and Lancashire, and the North-East of England, which were the areas that had high coal consumption, had in-migration and out-migration levels in all age-groups that were far below the National average.[43] In addition, our analysis allows for the factors that tend to drive migration such as education and socioeconomic status.[44] Finally, it is difficult to see how selective migration could explain the specific links between coal consumption and some conditions and not others (Table 5). Any undetected bias in our observational study would be most likely to be due to confounding from an unknown source. However, a sensitivity analyses suggested that such a variable would have to correlate very strongly ($r > 0.5$) with both domestic air pollution and respiratory disease, for example, to confound the relationships we have observed. There are no obvious candidates for such a confounder that were not addressed by our analysis. We have examined the effects of the major confounding variables that were available in the 1951 and 2001 census and that are likely to be related to both residence in an area of high pollution and to the various outcomes that we have studied. The 1951 census contained several additional measures of household amenities, which were also examined in regression analyses. We found that these did not explain any further variance in our analyses beyond that due to the confounding variables that we used in the final analyses. Adjusting the risk estimates associated with domestic consumption for social class, density, unemployment and education reduced their magnitude but they still remained strongly significant (Table

For peer review only - http://bmjopen.bmj.com/site/about/guidelines.xhtml

3 and Figure 2). However, it could be argued that some of these variables are so closely related to the measures of pollution that the regression-based adjustment has resulted in conservative risk estimates. Unsurprisingly, population density was highly correlated with domestic fuel use because fuel was rationed on a per household basis. In our analyses based on a published technique,[45] we were able to show that pollution had a more marked effect than population density but the close relationship between these variables prevented us from using this in the adjusted regression models and, as a result, we cannot confidently exclude the influence of early population density. However, we would also argue that, in this context, density may be a less important confounder than overcrowding. There is considerable observational evidence linking overcrowding with physical health, especially in adults[46] and in a review of the environmental factors most associated with health outcomes, Evans and Kantrowitz concluded that areal indices of density were less important than overcrowding in determining the health outcomes associated with high population density.[47] Although we did not have data on the prevalence of tobacco smoking, we do not think that this is likely to have seriously confounded our results as firstly, smoking is associated with low socioeconomic status,[48] which was included in our analyses and secondly, standardisation of the data for lung cancer rates, as a proxy for smoking, did not explain the correlations we observed.

Conclusions

Although this is an ecological study based on geographical correlations, the results raise the possibility that domestic air pollution, experienced over 60 years ago by young children, affects human health now, by increasing mortality from a wide variety of diseases. The effect of early life exposure was much stronger than that of current pollution assessed by concentrations of microparticulates. Importantly, the findings raise the possibility that the health effects of air pollution have been underestimated as most studies do not have data on early life exposure, where vulnerability may be greatest, and even the effects of the lower levels of pollution experienced by current Western populations appears to have been under-appreciated. Indeed, a recent mortality study based on the Medicare population suggests that a significant disease burden is associated with pollutant levels that are below current statutory standards.[49] The evidence that air pollution has been having a major detrimental impact on health for decades and continues to do so underlines the importance of routine environmental tracking and surveillance systems in detecting and avoiding the harmful health effects of environmental pollution. The data presented in this analysis also have implications for the long-term health of the populations of countries that still depend on large amounts of coal for their domestic markets. This includes newly industrialized countries such as India or China, where coal is a major energy source, and resource-poor countries, where indoor air pollution from cook stoves results in heavy exposure of women and young children to pollution.[3]

For peer review only - http://bmjopen.bmj.com/site/about/guidelines.xhtml

Acknowledgments: The authors are grateful to Laura Todd and her colleagues at the UK Office for National Statistics for their help in providing the mortality data. We also thank David Coggon for helpful comments on the manuscript.

REFERENCES

1. Landrigan PJ, Fuller R, Acosta NJR, et al. The Lancet Commission on pollution and health. *Lancet* 2017;http://dx.doi.org/10.1016/S0140-6736(17)32345-0 [published Online First: Oct 2017]
2. WHO. Effects of Air Pollution on Children's Health and Development. European Centre for Environment and Health. Bonn: WHO, 2005:1-184.
3. Mortimer K, Gordon SB, Jindal SK, et al. Household air pollution is a major avoidable risk factor for cardiorespiratory disease. *Chest* 2012;142(5):1308-15. doi: 1389935 [pii];10.1378/chest.12-1596 [doi]
4. UNICEF. Clear the air for children. New York: UNICEF, 2016:1-97.
5. Amegah AK, Quansah R, Jaakkola JJ. Household air pollution from solid fuel use and risk of adverse pregnancy outcomes: a systematic review and meta-analysis of the empirical evidence. *PloS one* 2014;9(12):e113920. doi: 10.1371/journal.pone.0113920
6. Bobak M, Richards M, Wadsworth M. Relation between children's height and outdoor air pollution from coal-burning sources in the British 1946 birth cohort. *IntArchOccupEnvironHealth* 2004;77(6):383-86. doi: 10.1007/s00420-004-0522-5 [doi]
7. Barker DJP. The Wellcome Foundation Lecture, 1994. The fetal origins of adult disease. *Proc Roy Soc Lond* 1995;262:37-43.
8. Brimblecombe P. The Big Smoke: Methuen 1987:1-204.
9. Logan WP. Mortality in the London fog incident, 1952. *Lancet* 1953;1(6755):336-8.
10. Ayres JG. Trends in air quality in the UK. *Allergy* 1997;52(38 Suppl):7-13.
11. Department for Business Energy & Industrial Strategy. Historical coal data: coal production, availability and consumption 1853 to 2015, 2015.
12. Douglas JWB, Waller RE. Air Pollution and respiratory infection in children. *Br J Prev Soc Med* 1966;20:1-8.
13. Daly C. Air pollution and causes of death. *Br J Prev Soc Med* 1959;13(1):14-27.
14. Gardner MJ, Crawford MD, Morris JN. Patterns of mortality in middle and early old age in the county boroughs of England and Wales. *Br J Prev Soc Med* 1969;23(3):133-40.
15. Ministry of Fuel and Power. Statistical Digest, 1954. London1955.
16. Gregory IN, Southall HR. Putting the Past in Its Place: the Great Britain Historical GIS In: Carver S, ed. Innovations in GIS 5: Taylor & Francis 1998:210-21.
17. Southall H. Rebuilding the Great Britain Historical GIS, Part 2 A Geo-Spatial Ontology of Administrative Units. *Hist Method* 2012;45(3):119-34. doi: 10.1080/01615440.2012.664101
18. Donkin A, Goldblatt P, Lynch K. Inequalities in life expectancy by social class, 1972-1999. *Health Statistics Quarlerly* 2002;15:5-15.
19. Gowers AM, Miller BG, Stedman JR. Estimating local mortality burdens associated with particulate air pollution. In: England PH, ed. London: Public Health England, 2014:1-40.
20. Paterson DA, Dorovitch MI, Farquhar DL, et al. Prospective study of necropsy audit of geriatric inpatient deaths. *J Clin Pathol* 1992;45(7):575-8.
21. Elliott P, Shaddick G, Wakefield JC, et al. Long-term associations of outdoor air pollution with mortality in Great Britain. *Thorax* 2007;62(12):1088-94. doi: 10.1136/thx.2006.076851
22. Bharadwaj P, Graff Zivin J, Mullins JT, et al. Early Life Exposure to the Great Smog of 1952 and the Development of Asthma. *American journal of respiratory and critical care medicine* 2016 doi: 10.1164/rccm.201603-0451OC

For peer review only - http://bmjopen.bmj.com/site/about/guidelines.xhtml

23. Hansell A, Ghosh RE, Blangiardo M, et al. Historic air pollution exposure and long-term mortality risks in England and Wales: prospective longitudinal cohort study. *Thorax* 2016;71(4):330-38. doi: 10.1136/thoraxjnl-2015-207111
24. Beach B, Walker Hanlon W. Coal smoke and mortality in an early industrial economy, 2016:1-75.
25. IARC. Indoor emissions from household combustion of coal. Lyon, France: WHO, 2012:515-38.
26. Hunt A, Abraham JL, Judson B, et al. Toxicologic and epidemiologic clues from the characterization of the 1952 London smog fine particulate matter in archival autopsy lung tissues. *Environmental health perspectives* 2003;111(9):1209-14.
27. Williams ML. Atmospheric dispersal of pollutants and the modelling of air pollution. In: RM H, ed. Pollution, causes, effects and control. 5th ed. London: Royal Society of Chemistry 2015:225-43.
28. Dockery DW, Pope CA, 3rd, Xu X, et al. An association between air pollution and mortality in six U.S. cities. *N Engl J Med* 1993;329(24):1753-9. doi: 10.1056/NEJM199312093292401
29. Laden F, Schwartz J, Speizer FE, et al. Reduction in fine particulate air pollution and mortality: Extended follow-up of the Harvard Six Cities study. *American journal of respiratory and critical care medicine* 2006;173(6):667-72. doi: 10.1164/rccm.200503-443OC
30. Pope CA, 3rd, Burnett RT, Thun MJ, et al. Lung cancer, cardiopulmonary mortality, and long-term exposure to fine particulate air pollution. *JAMA* 2002;287(9):1132-41.
31. Godfrey KM, Costello PM, Lillycrop KA. The developmental environment, epigenetic biomarkers and long-term health. *Journal of developmental origins of health and disease* 2015;6(5):399-406. doi: 10.1017/S204017441500121X
32. Lin HH, Suk CW, Lo HL, et al. Indoor air pollution from solid fuel and tuberculosis: a systematic review and meta-analysis. *The international journal of tuberculosis and lung disease : the official journal of the International Union against Tuberculosis and Lung Disease* 2014;18(5):613-21. doi: 10.5588/ijtld.13.0765
33. Wolf K, Stafoggia M, Cesaroni G, et al. Long-term Exposure to Particulate Matter Constituents and the Incidence of Coronary Events in 11 European Cohorts. *Epidemiology* 2015;26(4):565-74. doi: 10.1097/EDE.0000000000000300
34. Phillips DIW, Osmond C, Williams ML, et al. Air pollution in early life and adult mortality from chronic rheumatic heart disease. *Int J Epidemiol* 2017;46(4):1107-14. doi: 10.1093/ije/dyw249
35. Cai Y, Zhang B, Ke W, et al. Associations of Short-Term and Long-Term Exposure to Ambient Air Pollutants With Hypertension: A Systematic Review and Meta-Analysis. *Hypertension* 2016;68(1):62-70. doi: 10.1161/HYPERTENSIONAHA.116.07218
36. Trimble CL, Genkinger JM, Burke AE, et al. Active and passive cigarette smoking and the risk of cervical neoplasia. *Obstet Gynecol* 2005;105(1):174-81. doi: 10.1097/01.AOG.0000148268.43584.03
37. Norman P, Boyle P, Rees P. Selective migration, health and deprivation: a longitudinal analysis. *Soc Sci Med* 2005;60(12):2755-71. doi: 10.1016/j.socscimed.2004.11.008
38. Strachan DP, Leon DA, Dodgeon B. Mortality from cardiovascular disease among interregional migrants in England and Wales. *BMJ* 1995;310(6977):423-7.
39. Osmond C, Barker DJ, Slattery JM. Risk of death from cardiovascular disease and chronic bronchitis determined by place of birth in England and Wales. *JEpidemiolCommunity Health* 1990;44(2):139-41.
40. Fox AJ, Goldblatt PO. Socioeconomic differences in mortality. *Population Trends* 1982;27:8-13.
41. Boyle P. Population geography: migration and inequalities in mortality and morbidity. *Progress in Human Geography* 2004;28(6):767-76.
42. Pooley CG, Turnbull J. Migration trends in British rural areas from the 18th to the 20th centuries. *IntJPopulGeogr* 1996;2(3):215-37. doi: 10.1002/(SICI)1099-1220(199609)2:3<215::AID-IJPG35>3.0.CO;2-N [doi]
43. Dennett AR. Understanding Internal Migration in Britain at the Start of the 21st Century. University of Leeds, 2010.
44. Champion T. Migration and British Cities in the 1990s. *National Institute Economic Review* 1999;170:60-76.
45. King MD, Fountain C, Dakhlallah D, et al. Estimated autism risk and older reproductive age. *Am J Public Health* 2009;99(9):1673-9. doi: 10.2105/AJPH.2008.149021
46. Minister' OotDP. The Impact of Overcrowding on Health and Education: A review of the Evidence and Literature. London: , 2004:1-38.

For peer review only

For peer review only - http://bmjopen.bmj.com/site/about/guidelines.xhtml

47. Evans GW, Kantrowitz E. Socioeconomic status and health: The potential role of environmental risk exposure. *Annu Rev Publ Health* 2002;23:303-31. doi: DOI 10.1146/annurev.publhealth.23.112001.112349
48. Kleinschmidt I, Hills M, Elliott P. Smoking behaviour can be predicted by neighbourhood deprivation measures. *J Epidemiol Community Health* 1995;49 Suppl 2:S72-7.
49. Di Q, Wang Y, Zanobetti A, et al. Air Pollution and Mortality in the Medicare Population. *N Engl J Med* 2017;376(26):2513-22. doi: 10.1056/NEJMoa1702747

For peer review only

Table 1: Fuel consumption in England and Wales 1951/1952.

	Total annual consumption (tons x 1000)	Annual consumption per acre in the 342 LGDs (tons)	
		Median	Quartiles
Total Domestic	36,783	1.00	0.29,4.38
(Smokeless)	5,247	0.17	0.05,0.63
Total industrial	75,597	1.17	0.23,5.58
(Smokeless)	24,294	0.47	0.12,2.07
Carbonisation & briquetting	55,122	0.63	0.10,5.36
Electricity generation	33,304	0.07	0.00,1.85
Total	200,806	4.21	0.82,21.38

For peer review only - http://bmjopen.bmj.com/site/about/guidelines.xhtml

Table 2: The effect of fuel consumed in 1951/52 and socioeconomic indicators derived from the 1951 census on current causes of mortality in the 342 LGDs in England and Wales.

	Relative risk of mortality per SD change in fuel usage or socioeconomic indicator (95% CI)			
	**All Cause**	**Respiratory** (ICD9: 460 to 519, ICD10: J00 to J99)	**Cardiovascular** (ICD9: 390 to 459 ICD10: I00 to I99)	**Cancer** (ICD9: 140-208, ICD10: C00 to C97)
**Univariate analyses**				
Fuel consumed 1951/2				
Domestic (ex. smokeless)	1.124(1.123 to 1.126)	1.238(1.234 to 1.242)	1.138(1.136 to 1.140)	1.073(1.071 to 1.075)
Industrial (ex. smokeless)	1.129(1.128 to 1.131)	1.244(1.239 to 1.248)	1.152(1.150 to 1.154)	1.080(1.079 to 1.082)
Carbonization	1.120(1.119 to 1.122)	1.225(1.221 to 1.230)	1.135(1.133 to 1.137)	1.073(1.071 to 1.074)
Electricity generation	1.096(1.095 to 1.097)	1.181(1.177 to 1.185)	1.110(1.107 to 1.112)	1.054(1.052 to 1.056)
1951 census				
Social Class	1.117(1.115 to 1.118)	1.190(1.186 to 1.194)	1.144(1.142 to 1.146)	1.087(1.085 to 1.089)
Education	1.093(1.092 to 1.094)	1.154(1.150 to 1.158)	1.125(1.123 to 1.127)	1.064(1.062 to 1.066)
Crowding	1.106(1.105 to 1.108)	1.179(1.175 to 1.183)	1.126(1.124 to 1.128)	1.075(1.073 to 1.077)
Density	1.105(1.104 to 1.106)	1.206(1.202 to 1.211)	1.113(1.111 to 1.115)	1.059(1.057 to 1.061)
Unemployment	1.074(1.073 to 1.076)	1.113(1.109 to 1.117)	1.077(1.075 to 1.079)	1.055(1.053 to 1.057)
**Multivariate analysis**				
Fuel consumed 1951/2				
Domestic (ex. smokeless)	1.061(1.058 to 1.064)	1.127(1.118 to 1.137)	1.051(1.046 to 1.056)	1.027(1.023 to 1.031)
Industrial (ex. smokeless)	1.008(1.006 to 1.011)	1.031(1.023 to 1.039)	1.020(1.016 to 1.025)	1.011(1.007 to 1.015)
Carbonization	1.029(1.027 to 1.030)	1.044(1.038 to 1.050)	1.029(1.026 to 1.033)	1.019(1.016 to 1.022)
Electricity generation	1.002(1.000 to 1.003)	0.999(0.994 to 1.004)	1.005(1.002 to 1.008)	0.995(0.992 to 0.997)
1951 census				
Social Class	1.062(1.060 to 1.064)	1.094 (1.087 to 1.101)	1.058(1.054 to 1.062)	1.054(1.051 to 1.058)
Education	1.021(1.019 to 1.022)	1.027(1.021 to 1.032)	1.045(1.042 to 1.048)	1.007(1.005 to 1.010)
Crowding	1.005(1.004 to 1.007)	1.001(0.996 to 1.006)	1.012(1.009 to 1.014)	1.007(1.005 to 1.010)
Unemployment	1.032(1.031 to 1.034)	1.037(1.032 to 1.042)	1.036(1.034 to 1.039)	1.027(1.025 to 1.030)

For peer review only - http://bmjopen.bmj.com/site/about/guidelines.xhtml

For peer review only

Contd.

**Combined effects analysis of domestic usage and density**				
Unadjusted				
Sum	1.112(1.111 to 1.113)	1.219(1.215 to 1.223)	1.122(1.120 to 1.124)	1.064(1.062 to 1.066)
Difference (domestic – density)	1.074(1.073 to 1.076)	1.117(1.113 to 1.121)	1.095(1.093 to 1.097)	1.052(1.050 to 1.054)
Adjusted*				
Sum	1.054(1.051 to 1.056)	1.116(1.107 to 1.126)	1.042(1.037 to 1.047)	1.022(1.017 to 1.026)
Difference (domestic – density)	1.031(1.029 to 1.032)	1.048(1.044 to 1.053)	1.036(1.034 to 1.038)	1.018(1.016 to 1.020)

*Adjusted for social class, education, crowding, unemployment, industrial usage, and fuel used in carbonization & electricity generation.

For peer review only - http://bmjopen.bmj.com/site/about/guidelines.xhtml

Table 3: The effect of socioeconomic indicators derived from the 2001 census and current $PM_{2.5}$ concentrations on current causes of mortality in the 342 LGDs in England and Wales together with multivariate models evaluating the association between domestic coal usage and mortality following adjustment for the 1951 and 2001 socioeconomic indicators and current $PM_{2.5}$ exposure.

	Relative risk of mortality per SD change in socioeconomic indicator, particulate or fuel usage (95%CI)			
	**All Cause**	**Respiratory** (ICD9: 460 to 519, ICD10: J00 to J99)	**Cardiovascular** (ICD9: 390 to 459 ICD10: I00 to I99)	**Cancer** (ICD9: 140-208, ICD10: C00 to C97)
**Univariate analyses**				
2001 census				
Social Class	1.139(1.137 to1.140)	1.221(1.217 to 1.226)	1.170(1.168 to 1.173)	1.098(1.096 to 1.100)
Education	1.122(1.121 to 1.123)	1.195(1.191 to 1.199)	1.157(1.155 to 1.159)	1.087(1.085 to 1.089)
Crowding	1.101(1.099 to 1.102)	1.196(1.192 to 1.201)	1.117(1.115 to 1.119)	1.052(1.051 to 1.054)
Density	1.101(1.100 to 1.102)	1.196(1.192 to 1.200)	1.107(1.104 to 1.109)	1.059(1.057 to 1.061)
Unemployment	1.157(1.156 to 1.158)	1.270(1.266 to 1.275)	1.184(1.182 to 1.186)	1.099(1.097 to 1.101)
Current $PM_{2.5}$	1.027(1.026 to 1.028)	1.076(1.073 to 1.080)	1.028(1.027 to 1.030)	1.005(1.003 to 1.006)
**Multivariate analyses**				
Model 1 (effect of domestic usage allowing for 1951 socioeconomic indicators)	1.094(1.092 to 1.095)	1.195(1.190 to 1.200)	1.097 (1.095 to 1.100)	1.038 (1.033 to 1.043)
Model 2 (effect of domestic usage allowing for 2001 socioeconomic indicators and current $PM_{2.5}$)	1.081(1.078 to 1.084)	1.179(1.167 to 1.190)	1.088(1.082 to 1.093)	1.038(1.033 to 1.043)
Model 3 (effect of domestic usage allowing for 1951 and 2001 socioeconomic indicators and current $PM_{2.5}$)	1.084(1.080 to 1.087)	1.189(1.177 to 1.201)	1.088(1.083 to 1.094)	1.037 (1.032 to 1.042)

For peer review only

For peer review only - http://bmjopen.bmj.com/site/about/guidelines.xhtml

Table 4: Association between domestic fuel usage and major causes of non-cancer mortality among men and women aged 35-74 in the 342 areas.

	Relative risk per SD increase in domestic usage			
	Unadjusted (95%CI)		Adjusted* (95% CI)	
Respiratory				
Chronic obstructive pulmonary disease	1.253	1.245 to 1.260	1.142	1.122 to 1.163
Asthma	1.199	1.176 to 1.223	1.093	1.032 to 1.158
Pneumonia	1.262	1.254 to 1.269	1.227	1.205 to 1.250
Tuberculosis	1.734	1.676 to 1.793	1.298	1.167 to 1.444
Cardiovascular				
Ischaemic heart disease	1.141	1.138 to 1.143	1.102	1.095 to 1.110
Rheumatic heart disease	1.209	1.184 to 1.234	1.240	1.166 to 1.318
Stroke	1.137	1.133 to1.141	1.114	1.101 to 1.127
Hypertension	1.204	1.196 to 1.212	1.112	1.089 to 1.135

*Adjusted for 1951 and 2001 socioeconomic indicators and current $PM_{2.5}$ exposure.

For peer review only - http://bmjopen.bmj.com/site/about/guidelines.xhtml

Table 5: Association between domestic fuel usage and cancer mortality in the 342 areas.

Site	Relative risk per SD increase in domestic usage			
	Unadjusted (95%CI)		Adjusted* (95% CI)	
Lip, oral cavity and pharynx	1.220	1.204 to 1.236	1.207	1.161 to 1.255
Oesophagus	1.038	1.030 to 1.046	1.088	1.064 to 1.112
Stomach	1.171	1.161 to 1.182	1.117	1.088 to 1.147
Colon	0.996	0.989 to 1.002	1.027	1.007 to 1.048
Rectosigmoid junction, rectum and anus	1.073	1.063 to 1.082	1.039	1.012 to 1.066
Liver, gall bladder & bile ducts	1.193	1.179 to 1.206	1.053	1.019 to 1.089
Pancreas	1.022	1.014 to 1.030	0.977	0.956 to 0.999
Larynx	1.347	1.318 to 1.376	1.264	1.185 to 1.348
Trachea, bronchus and lung	1.195	1.190 to 1.199	1.126	1.115 to 1.137
Malignant melanoma	0.885	0.873 to 0.898	0.889	0.854 to 0.924
Breast	0.990	0.984 to 0.995	0.958	0.943 to 0.974
cervix	1.155	1.135 to 1.175	1.131	1.074 to 1.190
Uterus	1.032	1.015 to 1.049	0.930	0.886 to 0.975
Ovary	0.968	0.959 to 0.976	0.986	0.960 to 1.012
Prostate	0.989	0.981 to 0.998	0.962	0.939 to 0.986
Kidney	1.000	0.989 to1.010	1.003	0.973 to 1.035
Bladder	1.056	1.044 to 1.068	1.024	0.991 to 1.057
Brain , eye & CNS	0.952	0.943 to 0.961	0.962	0.936 to 0.988
Hodgkin's disease	1.030	0.994 to 1.067	0.919	0.829 to 1.018
Non-Hodgkin's lymphoma	1.024	1.014 to 1.034	0.952	0.925 to 0.979
Multiple myeloma	0.999	0.986 to 1.013	0.941	0.905 to 0.978
Leukaemia	1.008	0.998 to 1.019	0.961	0.931 to 0.991

*Adjusted for 1951 and 2001 socioeconomic indicators and current $PM_{2.5}$ exposure.

For peer review only - http://bmjopen.bmj.com/site/about/guidelines.xhtml

Figure 1: Domestic fuel consumption in England and Wales, 1951-52.

Figure 2: Association between quartiles of domestic fuel consumption (ex smokeless) and both all-cause and the major causes of mortality in men and women aged 35-74 in the 342 LGDs. (●unadjusted, ○ adjusted for socioeconomic indicators in 1951 and 2001 and current $PM_{2.5.}$; errors are 95% CI)

Figure 3: Hazard ratio of all-cause and the major causes of mortality per SD change in domestic consumption (ex. smokeless) according to birth year. Adjusted for socioeconomic indicators in 1951 and 2001 and current $PM_{2.5.}$

For peer review only

For peer review only - http://bmjopen.bmj.com/site/about/guidelines.xhtml

Footnotes

Contributors: DIWP discovered the dataset on coal consumption in 1951/2 and together with CO conceived the study. HS and PA advised on the use of the geographical data and carried out the redistricting analysis. DIWP, CO and AJ were responsible for the statistical analysis. The manuscript was written by DIWP, CO, AJ, HS and SH. All authors participated in a critical revision of the text and approved the final manuscript. DIWP is responsible for the overall content as the corresponding author.

Funding: The work was supported by the Medical Research Council.

Competing interests: None declared.

Ethics approval: As the study was based on routinely collected mortality data no ethics approval was necessary.

Data sharing: Under the MRC's "Policy and Guidance on Sharing of Research Data from Population and Patient Studies" the dataset is available upon request to diwp@mrc.soton.ac.uk.

For peer review only

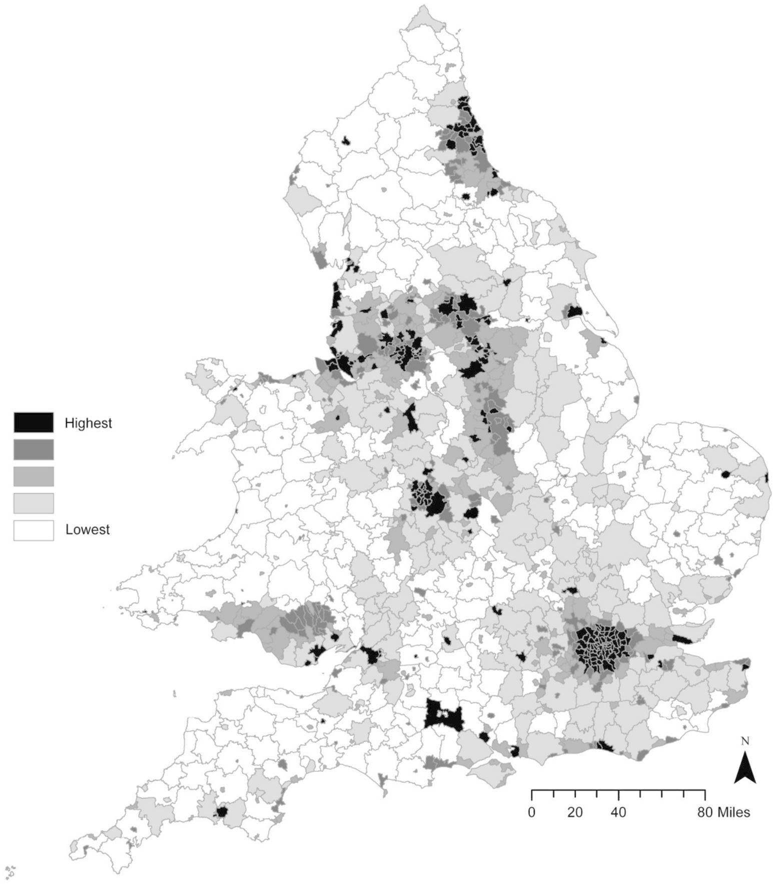

Figure 1: Domestic fuel consumption in England and Wales, 1951-52.

834x1178mm (51 x 51 DPI)

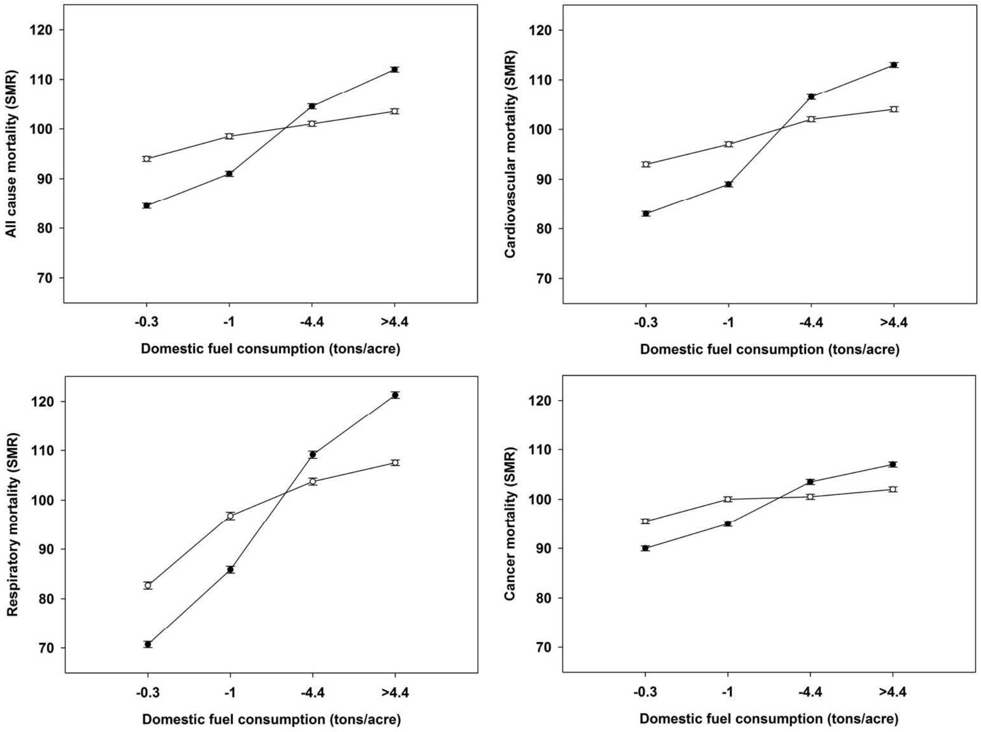

Figure 2: Association between quartiles of domestic fuel consumption (ex smokeless) and both all-cause and the major causes of mortality in men and women aged 35-74 in the 342 LGDs. (●unadjusted, ○ adjusted for socioeconomic indicators in 1951 and 2001 and current $PM_{2.5}$.; errors are 95% CI)

254x190mm (300 x 300 DPI)

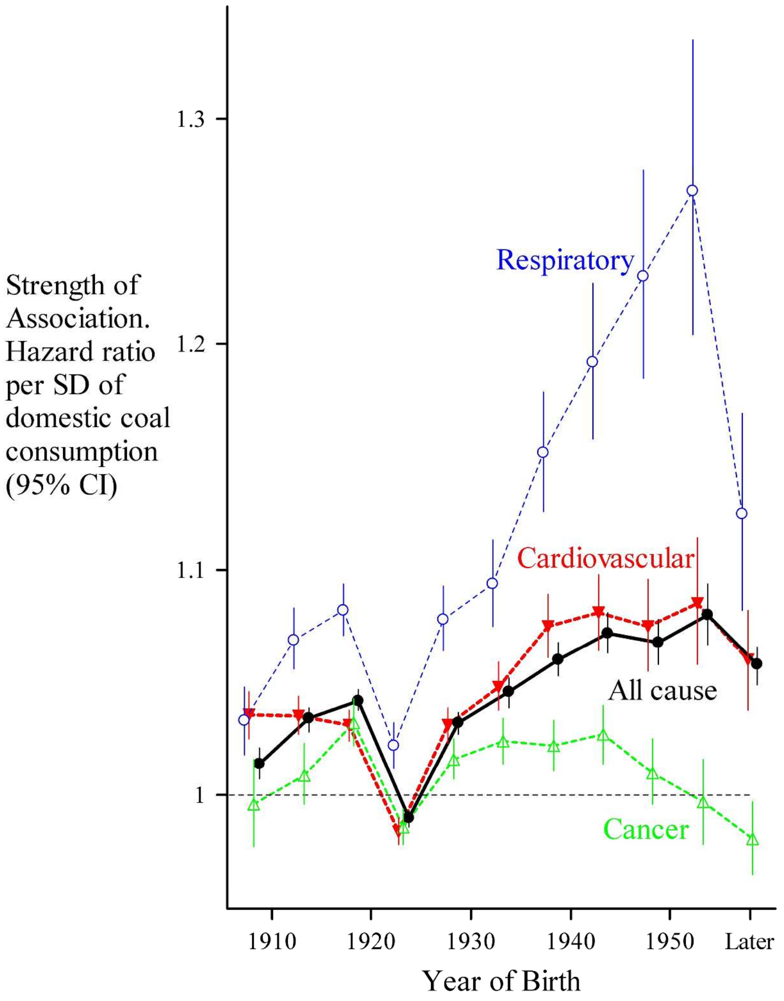

Figure 3: Hazard ratio of all-cause and the major causes of mortality per SD change in domestic consumption (ex. smokeless) according to birth year. Adjusted for socioeconomic indicators in 1951 and 2001 and current $PM_{2.5}$.

276x403mm (300 x 300 DPI)

Appendix

Cause of death	ICD 9 code	ICD 10 code	Number of deaths
**Cardiovascular disease**			
Ischaemic heart disease	410-414	I20-I25	710,979
Rheumatic heart disease	393-398	I05-I09	9,639
Cerebrovascular disease	430-438	I60-I69	264,853
Hypertension	401-405	I10-I15	84,208
**Respiratory disease**			
Chronic bronchitis and emphysema	490-492,496	J40-J44	105,690
Asthma	493	J45-J46	10,486
Pneumonia	480-486	J12-J18	107,848
Tuberculosis	010-018,137	A15-A19,B90	3,784
**Cancers**			
Lip, oral cavity and pharynx	140-149	C00-C14	22,992
Oesophagus	150	C15	65,854
Stomach	151	C16	49,634
Colon	153	C18	85,176
Rectosigmoid junction, rectum & anus	154	C19-C21	49,649
Liver, gall bladder and bile ducts	155,156	C22 – C24	31,240
Pancreas	157	C25	66,273
Larynx	161	C32	8,909
Trachea, bronchus & lung	162	C33,c34	335,217
Malignant melanoma	172	C43	20,045
Breast	174-175	C50	128,494
Cervix	180	C53	12,947
Uterus	179,182	C54-C55	14,315
Ovary	183	C56	47,895
Prostate	185	C61	54,521
Kidney	189	C64	33,232
Bladder	188	C67	32,353
Brain, Eye & CNS	191-192	C69-C72	43,811
Hodgkin's disease	201	C81	3,096
Non-Hodgkin's lymphoma	200,202	C82-C85	40,783
Multiple myeloma	203	C90	21,510
Leukaemia	204-208	C91-C95	33,526

For peer review only

STROBE 2007 (v4) checklist of items to be included in reports of observational studies in epidemiology*

Checklist for cohort, case-control, and cross-sectional studies (combined)

Section/Topic	Item #	Recommendation	Reported on page #
**Title and abstract**	1	(*a*) Indicate the study's design with a commonly used term in the title or the abstract	2
		(*b*) Provide in the abstract an informative and balanced summary of what was done and what was found	2
**Introduction**			
Background/rationale	2	Explain the scientific background and rationale for the investigation being reported	4
Objectives	3	State specific objectives, including any pre-specified hypotheses	4
**Methods**			
Study design	4	Present key elements of study design early in the paper	5-6
Setting	5	Describe the setting, locations, and relevant dates, including periods of recruitment, exposure, follow-up, and data collection	5-6
Participants	6	(*a*) *Cohort study*—Give the eligibility criteria, and the sources and methods of selection of participants. Describe methods of follow-up<br>*Case-control study*—Give the eligibility criteria, and the sources and methods of case ascertainment and control selection. Give the rationale for the choice of cases and controls<br>*Cross-sectional study*—Give the eligibility criteria, and the sources and methods of selection of participants	5-6
		(*b*) *Cohort study*—For matched studies, give matching criteria and number of exposed and unexposed<br>*Case-control study*—For matched studies, give matching criteria and the number of controls per case	N/a
Variables	7	Clearly define all outcomes, exposures, predictors, potential confounders, and effect modifiers. Give diagnostic criteria, if applicable	5-6, 20 (appendix)
Data sources/ measurement	8*	For each variable of interest, give sources of data and details of methods of assessment (measurement). Describe comparability of assessment methods if there is more than one group	*5-6*
Bias	9	Describe any efforts to address potential sources of bias	6
Study size	10	Explain how the study size was arrived at	N/A
Quantitative variables	11	Explain how quantitative variables were handled in the analyses. If applicable, describe which groupings were chosen and why	6
Statistical methods	12	(*a*) Describe all statistical methods, including those used to control for confounding	6
		(*b*) Describe any methods used to examine subgroups and interactions	N/A
		(*c*) Explain how missing data were addressed	N/A

For peer review only

		(*d*) *Cohort study*—If applicable, explain how loss to follow-up was addressed<br>*Case-control study*—If applicable, explain how matching of cases and controls was addressed<br>*Cross-sectional study*—If applicable, describe analytical methods taking account of sampling strategy	N/A
		(*e*) Describe any sensitivity analyses	11
**Results**			
Participants	13*	(a) Report numbers of individuals at each stage of study—eg numbers potentially eligible, examined for eligibility, confirmed eligible, included in the study, completing follow-up, and analysed	7
		(b) Give reasons for non-participation at each stage	N/A
		(c) Consider use of a flow diagram	N/A
Descriptive data	14*	(a) Give characteristics of study participants (eg demographic, clinical, social) and information on exposures and potential confounders	15 (Table 1)
		(b) Indicate number of participants with missing data for each variable of interest	N/A
		(c) *Cohort study*—Summarise follow-up time (eg, average and total amount)	5
Outcome data	15*	*Cohort study*—Report numbers of outcome events or summary measures over time	7
		Case-control study—Report numbers in each exposure category, or summary measures of exposure	N/A
		Cross-sectional study—Report numbers of outcome events or summary measures	N/A
Main results	16	(*a*) Give unadjusted estimates and, if applicable, confounder-adjusted estimates and their precision (eg, 95% confidence interval). Make clear which confounders were adjusted for and why they were included	16,17,18,19 (Tables 2-4)
		(*b*) Report category boundaries when continuous variables were categorized	N/A
		(*c*) If relevant, consider translating estimates of relative risk into absolute risk for a meaningful time period	N/A
Other analyses	17	Report other analyses done—eg analyses of subgroups and interactions, and sensitivity analyses	N/A
**Discussion**			
Key results	18	Summarise key results with reference to study objectives	9
Limitations	19	Discuss limitations of the study, taking into account sources of potential bias or imprecision. Discuss both direction and magnitude of any potential bias	11,12
Interpretation	20	Give a cautious overall interpretation of results considering objectives, limitations, multiplicity of analyses, results from similar studies, and other relevant evidence	12
Generalisability	21	Discuss the generalisability (external validity) of the study results	12
**Other information**			
Funding	22	Give the source of funding and the role of the funders for the present study and, if applicable, for the original study on which the present article is based	25

*Give information separately for cases and controls in case-control studies and, if applicable, for exposed and unexposed groups in cohort and cross-sectional studies.

For peer review only

Note: An Explanation and Elaboration article discusses each checklist item and gives methodological background and published examples of transparent reporting. The STROBE checklist is best used in conjunction with this article (freely available on the Web sites of PLoS Medicine at http://www.plosmedicine.org/, Annals of Internal Medicine at http://www.annals.org/, and Epidemiology at http://www.epidem.com/). Information on the STROBE Initiative is available at www.strobe-statement.org.

For peer review only

BMJ Open

Evaluating the long-term consequences of air pollution in early life: geographical correlations between coal consumption in 1951/2 and current mortality in England and Wales.

Journal:	*BMJ Open*
Manuscript ID	bmjopen-2017-018231.R2
Article Type:	Research
Date Submitted by the Author:	05-Dec-2017
Complete List of Authors:	Phillips, David; Medical Research Council<br>Osmond, Clive; Medical Research Council, Epidemiology Resource Centre<br>Southall, Humphrey; University of Portsmouth, Dept of Geography<br>Aucott, Paula; University of Portsmouth, Dept of Geography<br>Jones, Alexander; University of Oxford, Dept of Paediatrics<br>Holgate, Stephen; Southampton General Hospital, Medicine and Therapeutics
**Primary Subject Heading**:	Epidemiology
Secondary Subject Heading:	Occupational and environmental medicine, Public health
Keywords:	air pollution, Mortality, Developmental origins of health and diseasee

SCHOLARONE™ Manuscripts

For peer review only

Evaluating the long-term consequences of air pollution in early life: geographical correlations between coal consumption in 1951/2 and current mortality in England and Wales.

David IW Phillips, professor[1];Clive Osmond, professor of medical statistics[1]; Humphrey Southall, professor in historical geography[2]; Paula Aucott, senior research associate; Alexander Jones, paediatric cardiologist[3] ;Stephen T Holgate, professor of immunopharmacology.[4]

From

[1]The Medical Research Council's Lifecourse Epidemiology Unit, University of Southampton, Southampton, SO16 6YD. UK. ; [2]Department of Geography, University of Portsmouth, Portsmouth, PO1 2UP, UK. ; [3]Department of Paediatrics, University of Oxford, Oxford, OX3 9DU, UK. [4]Department of Medicine, University of Southampton, Southampton, SO16 6YD, UK.

Correspondence: DIW Phillips, MRC Lifecourse Epidemiology Unit, Southampton General Hospital, Southampton, UK.

Email: diwp@mrc.soton.ac.uk

Tel +44 (0) 23 80777624

FAX + 44 (0) 23 80704021

Short title: Long term effects of coal consumption

For peer review only - http://bmjopen.bmj.com/site/about/guidelines.xhtml

ABSTRACT

Objective

To evaluate the impact of early life air pollution on subsequent mortality.

Design

Geographical study

Setting

Local Government Districts within England and Wales

Exposure

Routinely collected geographical data on the use of coal and related solid fuels in 1951-2 were used as an index of air pollution.

Main outcome measures

We evaluated the relationship between these data and both all-cause and disease-specific mortality among men and women aged 35-74 in Local Government Districts between 1993 and 2012.

Results

Domestic (household) coal consumption had the most powerful effect on mortality. There were strong correlations between domestic coal use and all-cause mortality (relative risk per SD increase in fuel use 1.124, 95% CI = 1.123 to 1.126), and respiratory (1.238, 1.234 to 1.242), cardiovascular (1.138, 1.136 to 1.140), and cancer mortality (1.073, 1.071 to 1.075). These effects persisted after adjustment for socioeconomic indicators in 1951, current socioeconomic indicators and current pollution levels.

Conclusion

Coal was the major cause of pollution in the UK until the Clean Air Act of 1956 led to a rapid decline in consumption. These data suggest that coal-based pollution, experienced over 60 years ago in early life, affects human health now by increasing mortality from a wide variety of diseases.

For peer review only - http://bmjopen.bmj.com/site/about/guidelines.xhtml

Strengths and limitations of this study

•The first to evaluate the very long-term (60 year) mortality resulting from air pollution experienced in early life.

•Use of national mortality data with virtually complete ascertainment and a large number of deaths (> 3.5 million).

•Coal consumption data provide an integral of air pollution over a 12 month period.

•The analysis also allows for current pollution levels

•The study depends on the use of geographical correlations together with census-derived measures of socio-economic status; individual-level data are not available.

For peer review only

For peer review only - http://bmjopen.bmj.com/site/about/guidelines.xhtml

INTRODUCTION

Although air pollution has detrimental effects on health at all ages[1] there is accumulating evidence that the consequences of early-life exposure may be more important than that occurring at other stages in life. Developing tissues and organs are particularly susceptible due to their high cell proliferation rates in prenatal life and infancy. Postnatally, infants and young children have increased vulnerability due to immature detoxification systems, high infection rates and patterns of behaviour that increase their pollutant exposure.[2] Pregnant mothers exposed to air pollution are more likely to give birth prematurely and have offspring with intrauterine growth retardation and poor growth in infancy.[3 4] Such outcomes are linked with long-term adverse health effects, including increased risks of respiratory and cardiovascular disease.[5] An increasing body of evidence from both the medical and economic literature provide support for the adverse effects of early life pollutant exposure on a variety of health and human capital outcomes in later life. [2,6] This has important public health and policy implications not only in the developed world but also for resource poor countries where open fires and the consequent exposure of mothers and young children to pollutants is widespread.[2 7 8]

In common with many other countries, the UK had high levels of air pollution in the past.[9] Most of this was due to coal use in industry and as a domestic fuel, which gave rise to the "great stinking fogs" that previously characterised London and other major cities.[9] The London smog of 1952 killed an estimated 12,000 people[10] and led to the first UK Clean Air Act being passed in 1956. Following these events, a national survey of smoke and SO_2 concentrations was set up during the 1960s to monitor the progress of pollution control following the Act. From around 1960, there was a progressive reduction in black smoke and SO_2 emissions, with nitrogen oxides, ozone and microparticulates from transport becoming the dominant forms of air pollution.[11]

By the middle of the 20th century, more than 200 million tons of coal were being used per year in England and Wales[12] and yet little is known about the long-term health effects of the combustion of such vast quantities of solid fuel. Studies published in the 1950s and 1960s suggested that there were strong cross-sectional correlations of the amounts of coal used in domestic fires with both childhood respiratory disease [13] and many major causes of adult mortality[14 15] but it is not known if these effects have persisted. The present study is based on a dataset compiled by the Ministry of Fuel and Power that recorded the quantities of different types of solid fuel burnt annually between May 1951 and May 1952 in 1,145 areas of England and Wales. The extent to which exposure to the resulting pollutants may have influenced present day health was assessed by relating these data to mortality rates in the same areas 50-60 years later.

For peer review only - http://bmjopen.bmj.com/site/about/guidelines.xhtml

METHODS

Shortages of coal following the Second World War led to the continuation of a system of rationing and distribution that had been initiated in 1939. This system was controlled by local fuel overseers (LFOs) who were appointed for each local government district (LGD) or group of districts. The Ministry of Fuel and Power published estimates of the quantities of different types of solid fuel burnt annually in each of the 1,145 LFO areas between May 1951 and May 1952 in England and Wales – data were only published for these years.[16] The tables list the consumption of solid fuels classified by the main consumer groups in each LFO area. Domestic (household) supplies included coal, the amounts used in each LFO area based on the number of registered premises, and other solid fuels including coke, briquettes, ovoids and similar patent fuels where consumption was based on merchant's disposals. The annual household coal allowance was higher in the North (2,540 kg) than the South (1,727 kg). Miners' concessionary coal was also included in the estimates. Industrial fuels were predominantly coal and coke and there were separate listings of the quantities of solid fuel used in electricity generation and for carbonization or briquetting plants. The amounts of smokeless fuel (anthracite, dry steam coal, coke, Phurnacite, Coalite and Rexco) used in domestic and industrial premises were recorded separately.

The Office for National Statistics (ONS) provided extracts from all death certificates in England and Wales for each of the current LGDs during 1993 to 2012. This is the period covered by the ninth and tenth revisions of the International Classification of Disease (ICD). The ICD codes used to define causes of death are given in Appendix 1. Mortality rates were calculated using population estimates provided by the ONS for each LGD. The main technical problem was to relate the historical 1,145 LFO areas to the current 342 LGDs, following successive local government reorganisations in 1974, the 1990s, and 2009, together with numerous individual boundary changes. Detailed digital boundaries for the LGDs of England and Wales used in the 1951 census have already been constructed as part of the Great Britain Historical Geographical Information System (GIS).[17 18] As the LFO areas either exactly corresponded to LGDs or were defined as aggregates of them, constructing digital boundary data for LFO areas was straightforward. However, modern LGDs are not necessarily simple aggregates of the LFO areas, so the first stage of the redistricting procedure was to re-allocate the fuel consumption data to the 12,504 Civil Parishes of 1951. Digital boundaries already existed for the parishes, and the re-allocation was based on what proportion each parish's total population, as listed by the 1951 census, was of the overall population of the containing LFO area. The fuel data were then re-allocated to the modern areas by digitally overlaying the two sets of boundaries: whatever proportion of a 1951 parish's area fell within a modern district, that proportion of the parish's fuel consumption was assigned to the district. Air pollution from each source was estimated by calculating the consumption of solid fuel per acre.

For peer review only - http://bmjopen.bmj.com/site/about/guidelines.xhtml

Historically, air pollution was worse in predominantly industrial towns with a high population density; these towns also tended to have large populations of factory workers who left school early and worked in low status occupations.[14] These are important potential confounders in a study of air pollution and disease, as poverty and overcrowding are known to be associated with many of the major causes of mortality. To control for these we used a series of variables extracted from the published reports of the 1951 census: male unemployment; indices of social class; overcrowding, assessed as the proportion of households with more than one person per room; density, the number of people per acre; and educational achievement, indicated by the proportion of employed men in each area who left school before the age of 15 years. Social class was based on the Registrar General's occupation-based system that categorises people into five ordinal groups (I) Professional occupations, (II) Managerial and Technical occupations, (III) Skilled occupations, (IV) Partly-skilled occupations and (V) Unskilled occupations which were combined into a weighted score.[19] The census data were redistricted in the same way as for the fuel. Parallel data were obtained from the 2001 census: unemployment rate, social grade (combined into a weighted score), proportion of households with >1 person/room, persons/hectare, and proportion with no qualifications in each of the 342 LGDs. We also allowed for the impact of current levels of air pollution, which were obtained from published data reporting the modelled annual average concentrations of fine particulate matter ($PM_{2.5}$) in each of the 342 LGDs.[20]

Statistical methods

We studied deaths in people aged between 35 and 74 years, as this was the approximate age range of the generation who were in their first decade of life in 1951-52, and excluded older age groups where the cause of death was less likely to have been ascertained accurately.[21] We calculated the number of deaths expected for each five-year age and sex group within the LGDs by multiplying the number of people in each group by the national age- and sex-specific death rates in 1993-2012. We expressed the number of observed deaths in each area as a percentage of the expected deaths for that group, i.e. as standardised mortality ratios (SMRs). We used Poisson regression to model SMRs, expressing the effect of fuels and other variables on mortality in terms of the hazard ratio (or relative risk) per SD change in the explanatory variable. This weights the analyses appropriately to allow for variation in population size across LGDs. A Fisher-Yates transform was used to standardise pollution and socioeconomic variables. This yielded variables with zero means, unit standard deviations (SDs) and symmetrical distributions, allowing direct comparison of the strength of associations with these variables.

For peer review only - http://bmjopen.bmj.com/site/about/guidelines.xhtml

RESULTS

There were a total of 3,535,136 deaths in England and Wales during the 20-year period from 1993 to 2012 in the 35- to 74-yr age-group. Mortality rates (SMR) in the 342 LGDs ranged from 64.7 in East Dorset to 153.1 in Manchester. Table 1 shows the different uses of solid fuel during 1951/2 in England and Wales. Of the over 200 million tons distributed, 37.6% was used in industrial processes, 16.6% in electricity generation and 27.5% in carbonization or briquetting processes. Only 18.3% was used domestically. Smokeless fuel accounted for approximately 32.1% of industrial and 14.3% of domestic consumption. Figure 1 is a map of England and Wales showing the quintiles of domestic fuel consumed per acre. This was generally low in rural areas but was greatest in London and in the former industrial areas of the UK (South Wales, the Midlands, Lancashire, the West Riding of Yorkshire and Tyneside). The mean current $PM_{2.5}$ concentrations in the 342 areas was 9.48 (SD 1.67) $\mu g/m^3$ and ranged from 5.5 in Gwynedd (Wales) to 14.9 $\mu g/m^3$ in the City of Westminster, London.

Table 2 shows the relationship between the various sources of fuel burnt in 1951/2 (excluding smokeless fuels) and subsequent all-cause mortality or mortality due to respiratory, cardiovascular disease and cancer in the 342 areas. The influence of the 1951 census socioeconomic indicators (social class, education, crowding, density and unemployment) are also shown. In univariate analyses, all variables were significantly associated with both all-cause and cause-specific mortality rates although the effects of domestic and industrial consumption tended to be most strongly related to the mortality outcomes. Multivariate analyses were carried out to evaluate the relative effects of fuel usage and the major confounding variables derived from the 1951 census. This analysis shows that domestic consumption was strongly and significantly associated with both all-cause and cause-specific mortality rates, an effect which was markedly stronger than the other sources of fuel. Smokeless fuels represented less than 15% of the total fuels used (Table 1) and in separate regression analyses had no statistically significant independent effect on mortality rates. (Data not shown)

There was a very high correlation (r=0.97) between domestic consumption and population density, resulting in unacceptable collinearity in regression analyses that contained both variables. This occurred because both had the same denominator, the area of the LGD. To allow the influence of both of these factors to be modelled statistically, we created two further variables: the sum of and the difference between domestic use and density. These variables were uncorrelated (r=0.0). These were entered into the regression models in Table 2, which shows both the unadjusted coefficients and the coefficients adjusted for all other variables (Table 2 lower panel). The positive regression coefficients for the sum variable show that the joint effects of domestic use and density on all four causes of mortality were powerful. However, associations with the difference variable suggest that the influence of domestic use was stronger than that of density.

For peer review only - http://bmjopen.bmj.com/site/about/guidelines.xhtml

Table 3 shows the relationship between current socioeconomic indicators or microparticulate air pollution and all-cause or cause-specific mortality. The lower section of Table 3 shows that the effect of domestic coal usage is attenuated by successive adjustments for socioeconomic indicators from the 1951 census, for indicators from the 2001 census together with particulate exposure and for both of these sets of factors. However, in all cases the association with domestic coal consumption remained strong and statistically significant.

The effects of the potential confounders were tested singly and in combination. There was no evidence of non-linear relationships. Figure 2 shows the associations between quartiles of domestic fuel consumption and all-cause mortality or the major mortality categories. These associations were progressive and remained strong and statistically significant for each cause group after adjustment for social class, education, crowding, unemployment in 1951, and for current socioeconomic indicators and $PM_{2.5}$ concentrations. They were present in both genders and were similar during the ICD 9 and ICD 10 periods.

Figure 3 shows the strength of the geographical correlations between domestic consumption and current mortality according to successive birth cohorts. For all-cause and respiratory mortality, the strongest associations were observed among those born in 1952/3 suggesting that exposure around the time of birth had the greatest effect on mortality. For cardiovascular and cancer mortality, the peak associations were more blunted, suggesting that exposure over a wider age-range was associated with subsequent mortality.

Tables 4 & 5 show the relationship between domestic fuel consumption and specific causes of mortality. As before, the data are shown before and after adjustment for past and current socioeconomic indicators and current $PM_{2.5}$ levels. The respiratory conditions responsible for most deaths, chronic obstructive pulmonary disease and pneumonia, were strongly and consistently associated with domestic fuel consumption while associations with asthma mortality were somewhat weaker. However, the largest relative risks were with tuberculosis. While all four major causes of cardiovascular mortality were significantly associated with domestic consumption, the strongest relationship was with rheumatic heart disease.

The data on associations of domestic fuel use with mortality from specific cancers were striking (Table 5). There were strong relationships with epithelial cancers; lip, oral cavity and pharynx (adjusted hazard ratio, 1.207); laryngeal cancers (1.263) and lung (1.126). The upper gastrointestinal cancers; oesophagus (1.088), stomach (1.117) and liver (1.053), were more strongly associated with domestic pollution than the lower gastrointestinal cancers. In contrast, the major urinary, reproductive and haematological

For peer review only - http://bmjopen.bmj.com/site/about/guidelines.xhtml

cancers showed no increase in risk, with the exception of cervical cancer (1.131). Some cancers, for example melanoma and brain, were associated with a somewhat reduced relative risk.

DISCUSSION

Areas of the UK that had high domestic consumption of coal and related non-smokeless solid fuels in 1951/52 now have raised mortality from a wide variety of causes, including cardiovascular and respiratory diseases and certain cancers. The correlations are strong, statistically significant and independent of all available variables that might be considered major confounders, whether assessed in 1951 or currently, including social class, level of education, overcrowding, and unemployment.

Very few comparable data exist in the literature, particularly with such a long follow-up period. Our findings accord with an increasing body of evidence that early life exposure to air pollution has detrimental long-term health effects.[6 15 22] In particular, two recent studies demonstrated important findings. In the first, children exposed to the great London Smog of 1952 were found to have greater risk of asthma in adulthood, compared to unexposed children.[23] In the second, which was based on the ONS longitudinal study, air pollution concentrations at the site of residence, assessed every 10 years from 1971, were associated with increased total, respiratory and cardiovascular mortality.[24] Our conclusions also accord with historical studies of coal use and mortality in Britain, which estimated that each SD increase in coal use raised mortality by 5-15% in infants and 5% in adults.[25]

Our study necessarily depended on published crude estimates of coal consumption as an index of domestic air pollution. We contend that is very reasonable to assume that the quantity of coal burnt relates to the amount of pollution emitted although local factors such as climate and wind strength would also have a major influence. While there is no information about exposure to other pollutants these are likely to have been of minor importance compared with coal combustion. The consumption measures in our study were shown to correlate with subsequent measurements of pollution in the UK in the early 1960s,[13] when coal was still a major fuel and source of pollution. This approach is also supported by a number of studies carried out over the last 60 years, showing correlations with diverse health outcomes including reduced early growth,[3] chronic bronchitis and lung cancer.[13-15] While the consumption data come from a short time-period between 1951 and 1952, they are likely to reflect smoke emission over many years. UK coal production was fairly constant during the late 1940s, declined a little during the 1950s and only fell dramatically during the 1960s and subsequent decades.[12] In the post-war years, economic necessity drove the use of low-grade, bituminous domestic coal while better-quality "hard" coals were exported. A typical, inefficient domestic grate burning low-grade coal would have produced lots of smoke, rich in a wide variety of potentially toxic compounds, including heavy metals, sulphur and complex mixtures of aliphatic and aromatic hydrocarbons.[26] Confirmation of this can be found in the

For peer review only - http://bmjopen.bmj.com/site/about/guidelines.xhtml

lung tissue from autopsies of people exposed to the 1952 London smog, which was shown to contain both ultrafine carbonaceous and metal particulate matter.[27] Children would have been exposed both in the home and in their local environment although it is not clear from our data which of these would have been the most important source. However, our study suggests that it was domestic fuel consumption that had the greatest long-term adverse health effects, with industrial pollution or pollution from power stations or other sources causing relatively smaller effects (Table 2). The probable explanation for this is that most industrial and power station pollution was vented through tall chimneys, decreasing ground-level pollution in approximate proportion to the inverse square of chimney height.[28]

Current levels of pollution in the 342 local areas were based on ambient levels of fine particulate matter ($PM_{2.5}$) because large cohort studies have shown that this measure correlates best with long-term health effects of pollution.[29-31] The effects of $PM_{2.5}$ on current mortality (Table 3) in the present study are consistent with previously published estimates,[20] but importantly the mortality correlations with coal consumption persisted after adjustment for this measure of current pollution.

Our data do not allow determination of the age at which children were most vulnerable to the effects of pollution although the cohort-based analysis (Figure 3) suggests that exposures around the time of birth or infancy are likely to have been most important especially for all-cause and respiratory mortality. We can also only speculate on the mechanism by which air pollution arising from coal consumption, predominantly in a domestic setting, might increase the risk of mortality. There may have been direct mutagenic effects, especially in relation to the increased cancer risk. Another possibility is that the pollutants triggered mechanisms that selected alternative developmental pathways in the young, perhaps through epigenetic modification of gene expression.[32]

Elevated relative risks were observed for respiratory diseases, especially COPD, asthma and pneumonia, (Table 4) which is consistent with past evidence that black smoke exposure is a risk factor for these conditions.[14 15 22] The strongest association we found, however, was with tuberculosis, which is less supported by existing literature. A recent systematic review and meta-analysis concluded that the evidence for such an association was inconclusive but it was reliant on case-control and cross-sectional studies.[33] The findings in the present study raise the possibility that early exposure to air pollution could be a critical factor in determining susceptibility to tuberculosis although other factors such as population density cannot be completely excluded. Cardiovascular disease was also found to be strongly associated with domestic fuel consumption, which accords with earlier UK findings,[15 22] the Harvard Six Cities study,[30] and a multicentre study of European Cohorts.[34] The strength of the association was similar with ischaemic heart disease and stroke. The association with rheumatic heart disease was strong and confirms the findings of a previous study based on a subset of large Country Boroughs in England and Wales, and further supports the suggestion that an increase in susceptibility to infectious diseases is

For peer review only - http://bmjopen.bmj.com/site/about/guidelines.xhtml

linked with exposure to air pollution in infancy or early childhood.[35] An additional finding was a strong association with hypertension, which is supported by a meta-analysis that showed both short-term and long-term exposure to some air pollutants increases the risk of hypertension.[36] The data on cancers (Table 5) show wide variation in the level of associated risk. They show that the strongest associations are observed with epithelial cancers of the respiratory system (lip, oral cavity and pharynx; larynx; trachea and lung) and the upper gastrointestinal system (stomach and liver). The major reproductive, urinary and haematological cancers were not positively associated with domestic pollution with the exception of cervical cancer, for which there is evidence that cigarette smoking is an important co-factor.[37] Malignant melanoma had a lower relative risk in the areas with high consumption possibly because of reduced sunlight exposure. Some cancers showed small reductions in relative risk, for example brain cancer, and although the reasons are not clear, this may be due to residual confounding, which is evident because of the very large numbers of deaths and high sensitivity of this analysis.

There are a number of potential limitations to our study. An inevitable issue with evaluating the association of early exposures with causes of late-life mortality is the long latency between, in this case, the measurements of coal consumption and the outcomes. It could be argued that internal migration might have weakened the associations that we observed as selective migration has been proposed as an explanation for geographical variations in mortality. This is supported by some studies[38] but not others.[39 40] Migration is complex as, while migrants are generally healthier, those moving short distances tend to have higher mortality than those moving long distances.[41 42] Although we do not have migration data, most migration during the time period of this study occurred over short distances, with only a small minority migrating significant distances.[43] A more recent study of migration based on the 2001 census, while documenting the complex age and social class influences on migration, suggested that the declining industrial areas of South Wales, Yorkshire, Greater Manchester and Lancashire, and the North-East of England, which were the areas that had high coal consumption, had in-migration and out-migration levels in all age-groups that were far below the National average.[44] In addition, our analysis allows for the factors that tend to drive migration such as education and socioeconomic status.[45] Finally, it is difficult to see how selective migration could explain the specific links between coal consumption and some conditions and not others (Table 5). Any undetected bias in our observational study would be most likely to be due to confounding from an unknown source. However, a sensitivity analyses suggested that such a variable would have to correlate very strongly (r >0.5) with both domestic air pollution and respiratory disease, for example, to confound the relationships we have observed. There are no obvious candidates for such a confounder that were not addressed by our analysis, apart from lifetime exposure to pollution which is difficult to assess. We have examined the effects of the major confounding variables that were available in the 1951 and 2001 census and that are likely to be related to both residence in an area of high pollution and to the various outcomes that we have studied. The 1951

For peer review only - http://bmjopen.bmj.com/site/about/guidelines.xhtml

census contained several additional measures of household amenities, which were also examined in regression analyses. We found that these did not explain any further variance in our analyses beyond that due to the confounding variables that we used in the final analyses. Adjusting the risk estimates associated with domestic consumption for social class, density, unemployment and education reduced their magnitude but they still remained strongly significant (Table 3 and Figure 2). However, it could be argued that some of these variables are so closely related to the measures of pollution that the regression-based adjustment has resulted in conservative risk estimates. Unsurprisingly, population density was highly correlated with domestic fuel use because fuel was rationed on a per household basis. In our analyses based on a published technique,[46] we were able to show that pollution had a more marked effect than population density but the close relationship between these variables prevented us from using this in the adjusted regression models and, as a result, we cannot confidently exclude the influence of early population density. However, we would also argue that, in this context, density (population per unit area) may be a less important confounder than overcrowding (proportion of households with >1 person per room). There is considerable observational evidence linking overcrowding with physical health, especially in adults[47] and in a review of the environmental factors most associated with health outcomes, Evans and Kantrowitz concluded that areal indices of density were less important than overcrowding in determining the health outcomes associated with high population density.[48] Finally, our data showing linear relationships between fuel usage and mortality (Figure 2) are unlikely to be driven by the influence of density on pathogen transmission where saturation effects would be expected.[49] Although we did not have data on the prevalence of tobacco smoking, we do not think that this is likely to have seriously confounded our results as firstly, smoking is associated with low socioeconomic status,[50] which was included in our analyses and secondly, standardisation of the data for lung cancer rates, as a proxy for smoking, did not explain the correlations we observed.

Conclusions

Although this is an ecological study based on geographical correlations, the results raise the possibility that domestic air pollution, experienced over 60 years ago by young children, affects human health now, by increasing mortality from a wide variety of diseases. The effect of early life exposure was much stronger than that of current pollution assessed by concentrations of microparticulates. Importantly, the findings raise the possibility that the health effects of air pollution have been underestimated as most studies do not have data on early life exposure, where vulnerability may be greatest, and even the effects of the lower levels of pollution experienced by current Western populations appears to have been under-appreciated. Indeed, a recent mortality study based on the Medicare population suggests that a significant disease burden is associated with pollutant levels that are below current statutory standards.[51]

For peer review only - http://bmjopen.bmj.com/site/about/guidelines.xhtml

The evidence that air pollution has been having a major detrimental impact on health for decades and continues to do so underlines the importance of routine environmental tracking and surveillance systems in detecting and avoiding the harmful health effects of environmental pollution. The data presented in this analysis also have implications for the long-term health of the populations of countries that still depend on large amounts of coal for their domestic markets. This includes newly industrialized countries such as India or China, where coal is a major energy source, and resource-poor countries, where indoor air pollution from cook stoves results in heavy exposure of women and young children to pollution.[7]

Acknowledgments: The authors are grateful to Laura Todd and her colleagues at the UK Office for National Statistics for their help in providing the mortality data. We also thank David Coggon for helpful comments on the manuscript.

For peer review only

For peer review only - http://bmjopen.bmj.com/site/about/guidelines.xhtml

REFERENCES

1. Landrigan PJ, Fuller R, Acosta NJR, et al. The Lancet Commission on pollution and health. *Lancet* 2017;http://dx.doi.org/10.1016/S0140-6736(17)32345-0 [published Online First: Oct 2017]
2. WHO. Effects of Air Pollution on Children's Health and Development. European Centre for Environment and Health. Bonn: WHO, 2005:1-184.
3. Amegah AK, Quansah R, Jaakkola JJ. Household air pollution from solid fuel use and risk of adverse pregnancy outcomes: a systematic review and meta-analysis of the empirical evidence. *PloS one* 2014;9(12):e113920.
4. Bobak M, Richards M, Wadsworth M. Relation between children's height and outdoor air pollution from coal-burning sources in the British 1946 birth cohort. *IntArchOccupEnvironHealth* 2004;77(6):383-86.
5. Barker DJP. The Wellcome Foundation Lecture, 1994. The fetal origins of adult disease. *Proc Roy Soc Lond* 1995;262:37-43.
6. Currie J, Zivin JG, Mullins J, et al. What Do We Know About Short- and Long-Term Effects of Early-Life Exposure to Pollution? *Annu Rev Resour Econ* 2014;6:217-47.
7. Mortimer K, Gordon SB, Jindal SK, et al. Household air pollution is a major avoidable risk factor for cardiorespiratory disease. *Chest* 2012;142(5):1308-15.
8. UNICEF. Clear the air for children. New York: UNICEF, 2016:1-97.
9. Brimblecombe P. The Big Smoke: Methuen 1987:1-204.
10. Bell ML, Davis DL, Fletcher T. A retrospective assessment of mortality from the London smog episode of 1952: the role of influenza and pollution. *EnvironHealth Perspect* 2004;112(1):6-8.
11. Ayres JG. Trends in air quality in the UK. *Allergy* 1997;52(38 Suppl):7-13.
12. Department for Business Energy & Industrial Strategy. Historical coal data: coal production, availability and consumption 1853 to 2015. . https://www.gov.uk/government/statistical-data-sets/historical-coal-data-coal-production-availability-and-consumption-1853-to-2015.
13. Douglas JWB, Waller RE. Air Pollution and respiratory infection in children. *Br J Prev Soc Med* 1966;20:1-8.
14. Daly C. Air pollution and causes of death. *Br J Prev Soc Med* 1959;13(1):14-27.
15. Gardner MJ, Crawford MD, Morris JN. Patterns of mortality in middle and early old age in the county boroughs of England and Wales. *Br J Prev Soc Med* 1969;23(3):133-40.
16. Ministry of Fuel and Power. Statistical Digest, 1954. HMSO, London1955.
17. Gregory IN, Southall HR. Putting the Past in Its Place: the Great Britain Historical GIS In: Carver S, ed. Innovations in GIS 5: Taylor & Francis 1998:210-21.
18. Southall H. Rebuilding the Great Britain Historical GIS, Part 2 A Geo-Spatial Ontology of Administrative Units. *Hist Method* 2012;45(3):119-34.
19. Donkin A, Goldblatt P, Lynch K. Inequalities in life expectancy by social class, 1972-1999. *Health Statistics Quarlerly* 2002;15:5-15.
20. Gowers AM, Miller BG, Stedman JR. Estimating local mortality burdens associated with particulate air pollution. In: England PH, ed. London: Public Health England, 2014:1-40.
21. Paterson DA, Dorovitch MI, Farquhar DL, et al. Prospective study of necropsy audit of geriatric inpatient deaths. *J Clin Pathol* 1992;45(7):575-8.
22. Elliott P, Shaddick G, Wakefield JC, et al. Long-term associations of outdoor air pollution with mortality in Great Britain. *Thorax* 2007;62(12):1088-94.
23. Bharadwaj P, Graff Zivin J, Mullins JT, et al. Early Life Exposure to the Great Smog of 1952 and the Development of Asthma. *American journal of respiratory and critical care medicine* 2016 doi: 10.1164/rccm.201603-0451OC
24. Hansell A, Ghosh RE, Blangiardo M, et al. Historic air pollution exposure and long-term mortality risks in England and Wales: prospective longitudinal cohort study. *Thorax* 2016;71(4):330-38.
25. Beach B, Walker Hanlon W. Coal smoke and mortality in an early industrial economy, 2016:1-75. http://www.econ.ucla.edu/whanlon/papers/beach_hanlon_draft.pdf
26. IARC. Indoor emissions from household combustion of coal. Lyon, France: WHO, 2012:515-38.

For peer review only - http://bmjopen.bmj.com/site/about/guidelines.xhtml

27. Hunt A, Abraham JL, Judson B, et al. Toxicologic and epidemiologic clues from the characterization of the 1952 London smog fine particulate matter in archival autopsy lung tissues. *Environmental health perspectives* 2003;111(9):1209-14.
28. Williams ML. Atmospheric dispersal of pollutants and the modelling of air pollution. In: RM H, ed. Pollution, causes, effects and control. 5th ed. London: Royal Society of Chemistry 2015:225-43.
29. Dockery DW, Pope CA, 3rd, Xu X, et al. An association between air pollution and mortality in six U.S. cities. *N Engl J Med* 1993;329(24):1753-9.
30. Laden F, Schwartz J, Speizer FE, et al. Reduction in fine particulate air pollution and mortality: Extended follow-up of the Harvard Six Cities study. *American journal of respiratory and critical care medicine* 2006;173(6):667-72.
31. Pope CA, 3rd, Burnett RT, Thun MJ, et al. Lung cancer, cardiopulmonary mortality, and long-term exposure to fine particulate air pollution. *JAMA* 2002;287(9):1132-41.
32. Godfrey KM, Costello PM, Lillycrop KA. The developmental environment, epigenetic biomarkers and long-term health. *Journal of developmental origins of health and disease* 2015;6(5):399-406.
33. Lin HH, Suk CW, Lo HL, et al. Indoor air pollution from solid fuel and tuberculosis: a systematic review and meta-analysis. *The international journal of tuberculosis and lung disease : the official journal of the International Union against Tuberculosis and Lung Disease* 2014;18(5):613-21.
34. Wolf K, Stafoggia M, Cesaroni G, et al. Long-term Exposure to Particulate Matter Constituents and the Incidence of Coronary Events in 11 European Cohorts. *Epidemiology* 2015;26(4):565-74.
35. Phillips DIW, Osmond C, Williams ML, et al. Air pollution in early life and adult mortality from chronic rheumatic heart disease. *Int J Epidemiol* 2017;46(4):1107-14.
36. Cai Y, Zhang B, Ke W, et al. Associations of Short-Term and Long-Term Exposure to Ambient Air Pollutants With Hypertension: A Systematic Review and Meta-Analysis. *Hypertension* 2016;68(1):62-70.
37. Trimble CL, Genkinger JM, Burke AE, et al. Active and passive cigarette smoking and the risk of cervical neoplasia. *Obstet Gynecol* 2005;105(1):174-81.
38. Norman P, Boyle P, Rees P. Selective migration, health and deprivation: a longitudinal analysis. *Soc Sci Med* 2005;60(12):2755-71.
39. Strachan DP, Leon DA, Dodgeon B. Mortality from cardiovascular disease among interregional migrants in England and Wales. *BMJ* 1995;310(6977):423-7.
40. Osmond C, Barker DJ, Slattery JM. Risk of death from cardiovascular disease and chronic bronchitis determined by place of birth in England and Wales. *JEpidemiolCommunity Health* 1990;44(2):139-41.
41. Fox AJ, Goldblatt PO. Socioeconomic differences in mortality. *Population Trends* 1982;27:8-13.
42. Boyle P. Population geography: migration and inequalities in mortality and morbidity. *Progress in Human Geography* 2004;28(6):767-76.
43. Pooley CG, Turnbull J. Migration trends in British rural areas from the 18th to the 20th centuries. *IntJPopulGeogr* 1996;2(3):215-37.
44. Dennett AR. Understanding Internal Migration in Britain at the Start of the 21st Century. PhD Thesis, University of Leeds, 2010.
45. Champion T. Migration and British Cities in the 1990s. *National Institute Economic Review* 1999;170:60-76.
46. King MD, Fountain C, Dakhlallah D, et al. Estimated autism risk and older reproductive age. *Am J Public Health* 2009;99(9):1673-9.
47. Office of the Deputy Prime Minister. The Impact of Overcrowding on Health and Education: A review of the Evidence and Literature. London: Office of the Deputy Prime Minister, 2004:1-38.
48. Evans GW, Kantrowitz E. Socioeconomic status and health: The potential role of environmental risk exposure. *Annu Rev Publ Health* 2002;23:303-31.
49. Hu H, Nigmatulina K, Eckhoff P. The scaling of contact rates with population density for the infectious disease models. *Math Biosci* 2013;244(2):125-34.
50. Kleinschmidt I, Hills M, Elliott P. Smoking behaviour can be predicted by neighbourhood deprivation measures. *J Epidemiol Community Health* 1995;49 Suppl 2:S72-7.
51. Di Q, Wang Y, Zanobetti A, et al. Air Pollution and Mortality in the Medicare Population. *N Engl J Med* 2017;376(26):2513-22.

For peer review only - http://bmjopen.bmj.com/site/about/guidelines.xhtml

For peer review only

For peer review only - http://bmjopen.bmj.com/site/about/guidelines.xhtml

Table 1: Fuel consumption in England and Wales 1951/1952.

	Total annual consumption (tons x 1000)	Annual consumption per acre in the 342 LGDs (tons)	
		Median	Quartiles
Total Domestic	36,783	1.00	0.29,4.38
(Smokeless)	5,247	0.17	0.05,0.63
Total industrial	75,597	1.17	0.23,5.58
(Smokeless)	24,294	0.47	0.12,2.07
Carbonisation & briquetting	55,122	0.63	0.10,5.36
Electricity generation	33,304	0.07	0.00,1.85
Total	200,806	4.21	0.82,21.38

For peer review only - http://bmjopen.bmj.com/site/about/guidelines.xhtml

Table 2: The effect of fuel consumed in 1951/52 and socioeconomic indicators derived from the 1951 census on current causes of mortality in the 342 LGDs in England and Wales.

	Relative risk of mortality per SD change in fuel usage or socioeconomic indicator (95% CI)			
	**All Cause**	**Respiratory** (ICD9: 460 to 519, ICD10: J00 to J99)	**Cardiovascular** (ICD9: 390 to 459 ICD10: I00 to I99)	**Cancer** (ICD9: 140-208, ICD10: C00 to C97)
**Univariate analyses**				
Fuel consumed 1951/2				
Domestic (ex. smokeless)	1.124(1.123 to 1.126)	1.238(1.234 to 1.242)	1.138(1.136 to 1.140)	1.073(1.071 to 1.075)
Industrial (ex. smokeless)	1.129(1.128 to 1.131)	1.244(1.239 to 1.248)	1.152(1.150 to 1.154)	1.080(1.079 to 1.082)
Carbonization	1.120(1.119 to 1.122)	1.225(1.221 to 1.230)	1.135(1.133 to 1.137)	1.073(1.071 to 1.074)
Electricity generation	1.096(1.095 to 1.097)	1.181(1.177 to 1.185)	1.110(1.107 to 1.112)	1.054(1.052 to 1.056)
1951 census				
Social class score[+]	1.117(1.115 to 1.118)	1.190(1.186 to 1.194)	1.144(1.142 to 1.146)	1.087(1.085 to 1.089)
Low educational level	1.093(1.092 to 1.094)	1.154(1.150 to 1.158)	1.125(1.123 to 1.127)	1.064(1.062 to 1.066)
Crowding	1.106(1.105 to 1.108)	1.179(1.175 to 1.183)	1.126(1.124 to 1.128)	1.075(1.073 to 1.077)
Density	1.105(1.104 to 1.106)	1.206(1.202 to 1.211)	1.113(1.111 to 1.115)	1.059(1.057 to 1.061)
Unemployment	1.074(1.073 to 1.076)	1.113(1.109 to 1.117)	1.077(1.075 to 1.079)	1.055(1.053 to 1.057)
**Multivariate analysis**				
Fuel consumed 1951/2				
Domestic (ex. smokeless)	1.061(1.058 to 1.064)	1.127(1.118 to 1.137)	1.051(1.046 to 1.056)	1.027(1.023 to 1.031)
Industrial (ex. smokeless)	1.008(1.006 to 1.011)	1.031(1.023 to 1.039)	1.020(1.016 to 1.025)	1.011(1.007 to 1.015)
Carbonization	1.029(1.027 to 1.030)	1.044(1.038 to 1.050)	1.029(1.026 to 1.033)	1.019(1.016 to 1.022)
Electricity generation	1.002(1.000 to 1.003)	0.999(0.994 to 1.004)	1.005(1.002 to 1.008)	0.995(0.992 to 0.997)
1951 census				
Social Class	1.062(1.060 to 1.064)	1.094 (1.087 to 1.101)	1.058(1.054 to 1.062)	1.054(1.051 to 1.058)
Education	1.021(1.019 to 1.022)	1.027(1.021 to 1.032)	1.045(1.042 to 1.048)	1.007(1.005 to 1.010)
Crowding	1.005(1.004 to 1.007)	1.001(0.996 to 1.006)	1.012(1.009 to 1.014)	1.007(1.005 to 1.010)
Unemployment	1.032(1.031 to 1.034)	1.037(1.032 to 1.042)	1.036(1.034 to 1.039)	1.027(1.025 to 1.030)

For peer review only - http://bmjopen.bmj.com/site/about/guidelines.xhtml

Contd.

**Combined effects analysis of domestic usage and density**				
Unadjusted				
Sum	1.112(1.111 to 1.113)	1.219(1.215 to 1.223)	1.122(1.120 to 1.124)	1.064(1.062 to 1.066)
Difference (domestic – density)	1.074(1.073 to 1.076)	1.117(1.113 to 1.121)	1.095(1.093 to 1.097)	1.052(1.050 to 1.054)
Adjusted*				
Sum	1.054(1.051 to 1.056)	1.116(1.107 to 1.126)	1.042(1.037 to 1.047)	1.022(1.017 to 1.026)
Difference (domestic – density)	1.031(1.029 to 1.032)	1.048(1.044 to 1.053)	1.036(1.034 to 1.038)	1.018(1.016 to 1.020)

*Adjusted for social class, education, crowding, unemployment, industrial usage, and fuel used in carbonization & electricity generation.
+See methods

For peer review only

For peer review only - http://bmjopen.bmj.com/site/about/guidelines.xhtml

Table 3: The effect of socioeconomic indicators derived from the 2001 census and current $PM_{2.5}$ concentrations on current causes of mortality in the 342 LGDs in England and Wales together with multivariate models evaluating the association between domestic coal usage and mortality following adjustment for the 1951 and 2001 socioeconomic indicators and current $PM_{2.5}$ exposure.

	Relative risk of mortality per SD change in socioeconomic indicator, particulate or fuel usage (95%CI)			
	**All Cause**	**Respiratory** (ICD9: 460 to 519, ICD10: J00 to J99)	**Cardiovascular** (ICD9: 390 to 459 ICD10: I00 to I99)	**Cancer** (ICD9: 140-208, ICD10: C00 to C97)
**Univariate analyses**				
2001 census				
Social class score+	1.139(1.137 to1.140)	1.221(1.217 to 1.226)	1.170(1.168 to 1.173)	1.098(1.096 to 1.100)
Low educational level	1.122(1.121 to 1.123)	1.195(1.191 to 1.199)	1.157(1.155 to 1.159)	1.087(1.085 to 1.089)
Crowding	1.101(1.099 to 1.102)	1.196(1.192 to 1.201)	1.117(1.115 to 1.119)	1.052(1.051 to 1.054)
Density	1.101(1.100 to 1.102)	1.196(1.192 to 1.200)	1.107(1.104 to 1.109)	1.059(1.057 to 1.061)
Unemployment	1.157(1.156 to 1.158)	1.270(1.266 to 1.275)	1.184(1.182 to 1.186)	1.099(1.097 to 1.101)
Current $PM_{2.5}$	1.027(1.026 to 1.028)	1.076(1.073 to 1.080)	1.028(1.027 to 1.030)	1.005(1.003 to 1.006)
**Multivariate analyses**				
Model 1 (effect of domestic usage allowing for 1951 socioeconomic indicators)	1.094(1.092 to 1.095)	1.195(1.190 to 1.200)	1.097 (1.095 to 1.100)	1.038 (1.033 to 1.043)
Model 2 (effect of domestic usage allowing for 2001 socioeconomic indicators and current $PM_{2.5}$)	1.081(1.078 to 1.084)	1.179(1.167 to 1.190)	1.088(1.082 to 1.093)	1.038(1.033 to 1.043)
Model 3 (effect of domestic usage allowing for 1951 and 2001 socioeconomic indicators and current $PM_{2.5}$)	1.084(1.080 to 1.087)	1.189(1.177 to 1.201)	1.088(1.083 to 1.094)	1.037 (1.032 to 1.042)

+See methods

For peer review only - http://bmjopen.bmj.com/site/about/guidelines.xhtml

Table 4: Association between domestic fuel usage and major causes of non-cancer mortality among men and women aged 35-74 in the 342 areas.

	Relative risk per SD increase in domestic usage			
	Unadjusted (95%CI)		Adjusted* (95% CI)	
Respiratory				
Chronic obstructive pulmonary disease	1.253	1.245 to 1.260	1.142	1.122 to 1.163
Asthma	1.199	1.176 to 1.223	1.093	1.032 to 1.158
Pneumonia	1.262	1.254 to 1.269	1.227	1.205 to 1.250
Tuberculosis	1.734	1.676 to 1.793	1.298	1.167 to 1.444
Cardiovascular				
Ischaemic heart disease	1.141	1.138 to 1.143	1.102	1.095 to 1.110
Rheumatic heart disease	1.209	1.184 to 1.234	1.240	1.166 to 1.318
Stroke	1.137	1.133 to1.141	1.114	1.101 to 1.127
Hypertension	1.204	1.196 to 1.212	1.112	1.089 to 1.135

*Adjusted for 1951 and 2001 socioeconomic indicators and current $PM_{2.5}$ exposure.

For peer review only - http://bmjopen.bmj.com/site/about/guidelines.xhtml

Table 5: Association between domestic fuel usage and cancer mortality in the 342 areas.

Site	Relative risk per SD increase in domestic usage			
	Unadjusted (95%CI)		Adjusted* (95% CI)	
Lip, oral cavity and pharynx	1.220	1.204 to 1.236	1.207	1.161 to 1.255
Oesophagus	1.038	1.030 to 1.046	1.088	1.064 to 1.112
Stomach	1.171	1.161 to 1.182	1.117	1.088 to 1.147
Colon	0.996	0.989 to 1.002	1.027	1.007 to 1.048
Rectosigmoid junction, rectum and anus	1.073	1.063 to 1.082	1.039	1.012 to 1.066
Liver, gall bladder & bile ducts	1.193	1.179 to 1.206	1.053	1.019 to 1.089
Pancreas	1.022	1.014 to 1.030	0.977	0.956 to 0.999
Larynx	1.347	1.318 to 1.376	1.264	1.185 to 1.348
Trachea, bronchus and lung	1.195	1.190 to 1.199	1.126	1.115 to 1.137
Malignant melanoma	0.885	0.873 to 0.898	0.889	0.854 to 0.924
Breast	0.990	0.984 to 0.995	0.958	0.943 to 0.974
cervix	1.155	1.135 to 1.175	1.131	1.074 to 1.190
Uterus	1.032	1.015 to 1.049	0.930	0.886 to 0.975
Ovary	0.968	0.959 to 0.976	0.986	0.960 to 1.012
Prostate	0.989	0.981 to 0.998	0.962	0.939 to 0.986
Kidney	1.000	0.989 to1.010	1.003	0.973 to 1.035
Bladder	1.056	1.044 to 1.068	1.024	0.991 to 1.057
Brain , eye & CNS	0.952	0.943 to 0.961	0.962	0.936 to 0.988
Hodgkin's disease	1.030	0.994 to 1.067	0.919	0.829 to 1.018
Non-Hodgkin's lymphoma	1.024	1.014 to 1.034	0.952	0.925 to 0.979
Multiple myeloma	0.999	0.986 to 1.013	0.941	0.905 to 0.978
Leukaemia	1.008	0.998 to 1.019	0.961	0.931 to 0.991

*Adjusted for 1951 and 2001 socioeconomic indicators and current $PM_{2.5}$ exposure.

For peer review only - http://bmjopen.bmj.com/site/about/guidelines.xhtml

Figure 1: Domestic fuel consumption in England and Wales, 1951-52.

Figure 2: Association between quartiles of domestic fuel consumption (ex smokeless) and both all-cause and the major causes of mortality in men and women aged 35-74 in the 342 LGDs. (●unadjusted, ○ adjusted for socioeconomic indicators in 1951 and 2001 and current $PM_{2.5.}$; errors are 95% CI)

Figure 3: Hazard ratio of all-cause and the major causes of mortality per SD change in domestic consumption (ex. smokeless) according to birth year. Adjusted for socioeconomic indicators in 1951 and 2001 and current $PM_{2.5.}$

For peer review only

Footnotes

Contributors: DIWP discovered the dataset on coal consumption in 1951/2 and together with CO conceived the study. HS and PA advised on the use of the geographical data and carried out the redistricting analysis. DIWP, CO and AJ were responsible for the statistical analysis. The manuscript was written by DIWP, CO, AJ, HS and SH. All authors participated in a critical revision of the text and approved the final manuscript. DIWP is responsible for the overall content as the corresponding author.

Funding: The work was supported by the Medical Research Council.

Competing interests: None declared.

Ethics approval: As the study was based on routinely collected mortality data no ethics approval was necessary.

Data sharing: Under the MRC's "Policy and Guidance on Sharing of Research Data from Population and Patient Studies" the dataset is available upon request to diwp@mrc.soton.ac.uk.

For peer review only

For peer review only

For peer review only - http://bmjopen.bmj.com/site/about/guidelines.xhtml

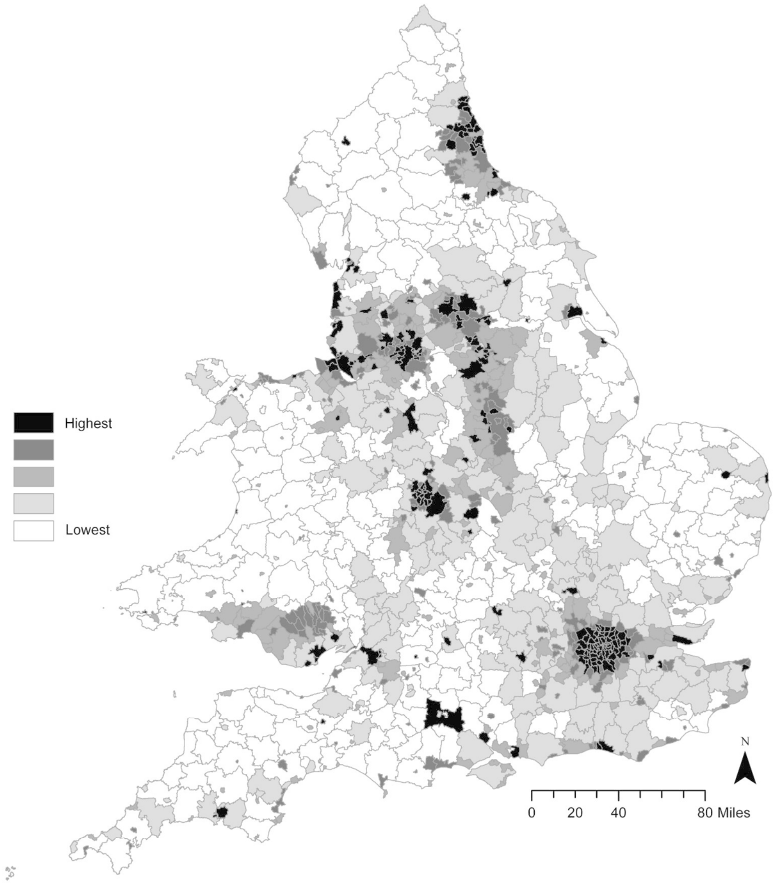

Figure 1: Domestic fuel consumption in England and Wales, 1951-52.

834x1178mm (51 x 51 DPI)

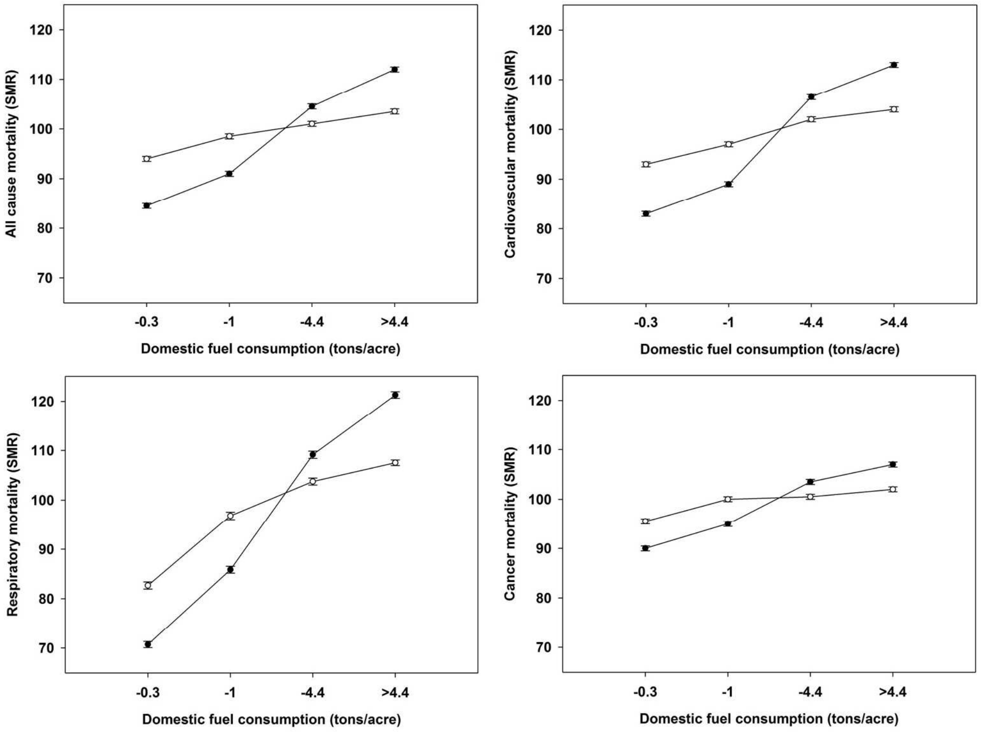

Figure 2: Association between quartiles of domestic fuel consumption (ex smokeless) and both all-cause and the major causes of mortality in men and women aged 35-74 in the 342 LGDs. (•unadjusted, ○ adjusted for socioeconomic indicators in 1951 and 2001 and current $PM_{2.5}$.; errors are 95% CI)

254x190mm (300 x 300 DPI)

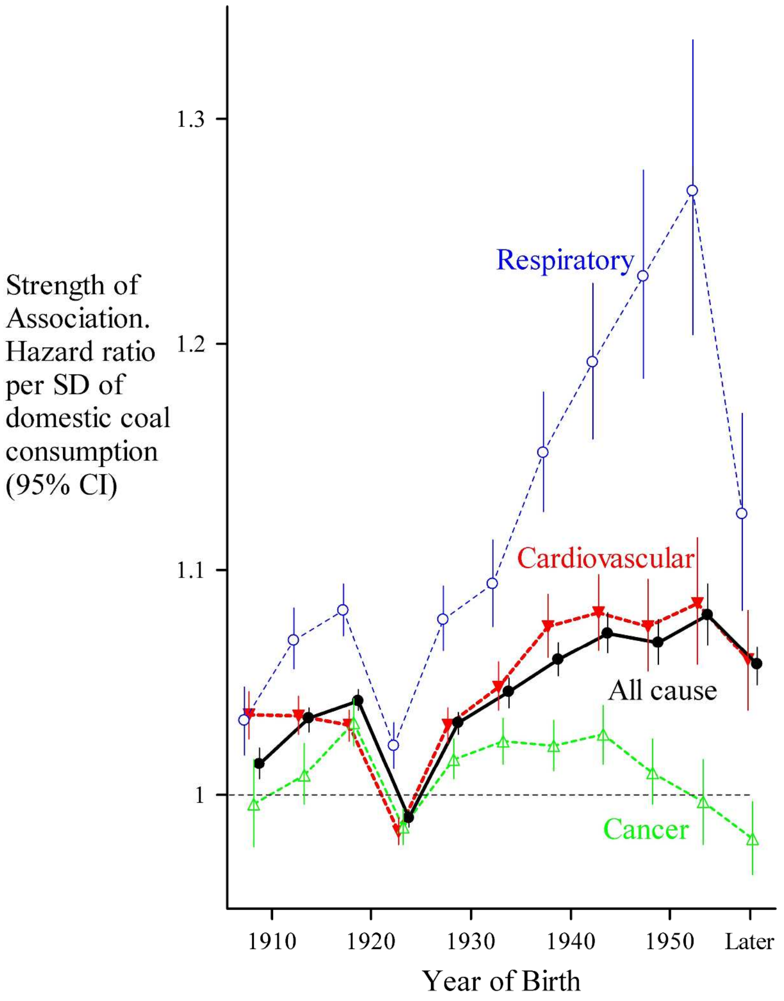

Figure 3: Hazard ratio of all-cause and the major causes of mortality per SD change in domestic consumption (ex. smokeless) according to birth year. Adjusted for socioeconomic indicators in 1951 and 2001 and current $PM_{2.5}$.

276x403mm (300 x 300 DPI)

Appendix

Cause of death	ICD 9 code	ICD 10 code	Number of deaths
**Cardiovascular disease**			
Ischaemic heart disease	410-414	I20-I25	710,979
Rheumatic heart disease	393-398	I05-I09	9,639
Cerebrovascular disease	430-438	I60-I69	264,853
Hypertension	401-405	I10-I15	84,208
**Respiratory disease**			
Chronic bronchitis and emphysema	490-492,496	J40-J44	105,690
Asthma	493	J45-J46	10,486
Pneumonia	480-486	J12-J18	107,848
Tuberculosis	010-018,137	A15-A19,B90	3,784
**Cancers**			
Lip, oral cavity and pharynx	140-149	C00-C14	22,992
Oesophagus	150	C15	65,854
Stomach	151	C16	49,634
Colon	153	C18	85,176
Rectosigmoid junction, rectum & anus	154	C19-C21	49,649
Liver, gall bladder and bile ducts	155,156	C22 – C24	31,240
Pancreas	157	C25	66,273
Larynx	161	C32	8,909
Trachea, bronchus & lung	162	C33,c34	335,217
Malignant melanoma	172	C43	20,045
Breast	174-175	C50	128,494
Cervix	180	C53	12,947
Uterus	179,182	C54-C55	14,315
Ovary	183	C56	47,895
Prostate	185	C61	54,521
Kidney	189	C64	33,232
Bladder	188	C67	32,353
Brain, Eye & CNS	191-192	C69-C72	43,811
Hodgkin's disease	201	C81	3,096
Non-Hodgkin's lymphoma	200,202	C82-C85	40,783
Multiple myeloma	203	C90	21,510
Leukaemia	204-208	C91-C95	33,526

For peer review only

STROBE 2007 (v4) checklist of items to be included in reports of observational studies in epidemiology*
Checklist for cohort, case-control, and cross-sectional studies (combined)

Section/Topic	Item #	Recommendation	Reported on page #
**Title and abstract**	1	(*a*) Indicate the study's design with a commonly used term in the title or the abstract	2
		(*b*) Provide in the abstract an informative and balanced summary of what was done and what was found	2
**Introduction**			
Background/rationale	2	Explain the scientific background and rationale for the investigation being reported	4
Objectives	3	State specific objectives, including any pre-specified hypotheses	4
**Methods**			
Study design	4	Present key elements of study design early in the paper	5-6
Setting	5	Describe the setting, locations, and relevant dates, including periods of recruitment, exposure, follow-up, and data collection	5-6
Participants	6	(*a*) *Cohort study*—Give the eligibility criteria, and the sources and methods of selection of participants. Describe methods of follow-up<br>*Case-control study*—Give the eligibility criteria, and the sources and methods of case ascertainment and control selection. Give the rationale for the choice of cases and controls<br>*Cross-sectional study*—Give the eligibility criteria, and the sources and methods of selection of participants	5-6
		(*b*) *Cohort study*—For matched studies, give matching criteria and number of exposed and unexposed<br>*Case-control study*—For matched studies, give matching criteria and the number of controls per case	N/a
Variables	7	Clearly define all outcomes, exposures, predictors, potential confounders, and effect modifiers. Give diagnostic criteria, if applicable	5-6, 20 (appendix)
Data sources/ measurement	8*	For each variable of interest, give sources of data and details of methods of assessment (measurement). Describe comparability of assessment methods if there is more than one group	*5-6*
Bias	9	Describe any efforts to address potential sources of bias	6
Study size	10	Explain how the study size was arrived at	N/A
Quantitative variables	11	Explain how quantitative variables were handled in the analyses. If applicable, describe which groupings were chosen and why	6
Statistical methods	12	(*a*) Describe all statistical methods, including those used to control for confounding	6
		(*b*) Describe any methods used to examine subgroups and interactions	N/A
		(*c*) Explain how missing data were addressed	N/A

For peer review only

		(*d*) *Cohort study*—If applicable, explain how loss to follow-up was addressed<br>*Case-control study*—If applicable, explain how matching of cases and controls was addressed<br>*Cross-sectional study*—If applicable, describe analytical methods taking account of sampling strategy	N/A
		(*e*) Describe any sensitivity analyses	11
**Results**			
Participants	13*	(a) Report numbers of individuals at each stage of study—eg numbers potentially eligible, examined for eligibility, confirmed eligible, included in the study, completing follow-up, and analysed	7
		(b) Give reasons for non-participation at each stage	N/A
		(c) Consider use of a flow diagram	N/A
Descriptive data	14*	(a) Give characteristics of study participants (eg demographic, clinical, social) and information on exposures and potential confounders	15 (Table 1)
		(b) Indicate number of participants with missing data for each variable of interest	N/A
		(c) *Cohort study*—Summarise follow-up time (eg, average and total amount)	5
Outcome data	15*	*Cohort study*—Report numbers of outcome events or summary measures over time	7
		Case-control study—Report numbers in each exposure category, or summary measures of exposure	N/A
		Cross-sectional study—Report numbers of outcome events or summary measures	N/A
Main results	16	(*a*) Give unadjusted estimates and, if applicable, confounder-adjusted estimates and their precision (eg, 95% confidence interval). Make clear which confounders were adjusted for and why they were included	16,17,18,19 (Tables 2-4)
		(*b*) Report category boundaries when continuous variables were categorized	N/A
		(*c*) If relevant, consider translating estimates of relative risk into absolute risk for a meaningful time period	N/A
Other analyses	17	Report other analyses done—eg analyses of subgroups and interactions, and sensitivity analyses	N/A
**Discussion**			
Key results	18	Summarise key results with reference to study objectives	9
Limitations	19	Discuss limitations of the study, taking into account sources of potential bias or imprecision. Discuss both direction and magnitude of any potential bias	11,12
Interpretation	20	Give a cautious overall interpretation of results considering objectives, limitations, multiplicity of analyses, results from similar studies, and other relevant evidence	12
Generalisability	21	Discuss the generalisability (external validity) of the study results	12
**Other information**			
Funding	22	Give the source of funding and the role of the funders for the present study and, if applicable, for the original study on which the present article is based	25

*Give information separately for cases and controls in case-control studies and, if applicable, for exposed and unexposed groups in cohort and cross-sectional studies.

For peer review only

Note: An Explanation and Elaboration article discusses each checklist item and gives methodological background and published examples of transparent reporting. The STROBE checklist is best used in conjunction with this article (freely available on the Web sites of PLoS Medicine at http://www.plosmedicine.org/, Annals of Internal Medicine at http://www.annals.org/, and Epidemiology at http://www.epidem.com/). Information on the STROBE Initiative is available at www.strobe-statement.org.

For peer review only

BMJ Open

Evaluating the long-term consequences of air pollution in early life: geographical correlations between coal consumption in 1951/2 and current mortality in England and Wales.

Journal:	*BMJ Open*
Manuscript ID	bmjopen-2017-018231.R3
Article Type:	Research
Date Submitted by the Author:	22-Mar-2018
Complete List of Authors:	Phillips, David; Medical Research Council<br>Osmond, Clive; Medical Research Council, Epidemiology Resource Centre<br>Southall, Humphrey; University of Portsmouth, Dept of Geography<br>Aucott, Paula; University of Portsmouth, Dept of Geography<br>Jones, Alexander; University of Oxford, Dept of Paediatrics<br>Holgate, Stephen; Southampton General Hospital, Medicine and Therapeutics
**Primary Subject Heading**:	Epidemiology
Secondary Subject Heading:	Occupational and environmental medicine, Public health
Keywords:	air pollution, Mortality, Developmental origins of health and diseasee

SCHOLARONE™ Manuscripts

For peer review only

Evaluating the long-term consequences of air pollution in early life: geographical correlations between coal consumption in 1951/2 and current mortality in England and Wales.

David IW Phillips, professor[1];Clive Osmond, professor of medical statistics[1]; Humphrey Southall, professor in historical geography[2]; Paula Aucott, senior research associate; Alexander Jones, paediatric cardiologist[3] ;Stephen T Holgate, professor of immunopharmacology.[4]

From

[1]The Medical Research Council's Lifecourse Epidemiology Unit, University of Southampton, Southampton, SO16 6YD. UK. ; [2]Department of Geography, University of Portsmouth, Portsmouth, PO1 2UP, UK. ; [3]Department of Paediatrics, University of Oxford, Oxford, OX3 9DU, UK. [4]Department of Medicine, University of Southampton, Southampton, SO16 6YD, UK.

Correspondence: DIW Phillips, MRC Lifecourse Epidemiology Unit, Southampton General Hospital, Southampton, UK.

Email: diwp@mrc.soton.ac.uk

Tel +44 (0) 23 80777624

FAX + 44 (0) 23 80704021

Short title: Long term effects of coal consumption

For peer review only - http://bmjopen.bmj.com/site/about/guidelines.xhtml

ABSTRACT

Objective

To evaluate associations between early life air pollution and subsequent mortality.

Design

Geographical study

Setting

Local Government Districts within England and Wales

Exposure

Routinely collected geographical data on the use of coal and related solid fuels in 1951-2 were used as an index of air pollution.

Main outcome measures

We evaluated the relationship between these data and both all-cause and disease-specific mortality among men and women aged 35-74 in Local Government Districts between 1993 and 2012.

Results

Domestic (household) coal consumption had the most powerful associations with mortality. There were strong correlations between domestic coal use and all-cause mortality (relative risk per SD increase in fuel use 1.124, 95% CI = 1.123 to 1.126), and respiratory (1.238, 1.234 to 1.242), cardiovascular (1.138, 1.136 to 1.140), and cancer mortality (1.073, 1.071 to 1.075). These effects persisted after adjustment for socioeconomic indicators in 1951, current socioeconomic indicators and current pollution levels.

Conclusion

Coal was the major cause of pollution in the UK until the Clean Air Act of 1956 led to a rapid decline in consumption. These data suggest that coal-based pollution, experienced over 60 years ago in early life, affects human health now by increasing mortality from a wide variety of diseases.

For peer review only - http://bmjopen.bmj.com/site/about/guidelines.xhtml

For peer review only

Strengths and limitations of this study

•The first to evaluate the very long-term (60 year) mortality resulting from air pollution experienced in early life.

•Use of national mortality data with virtually complete ascertainment and a large number of deaths (> 3.5 million).

•Coal consumption data provide an integral of air pollution over a 12 month period.

•The analysis also allows for current pollution levels

•The study depends on the use of geographical correlations together with census-derived measures of socio-economic status; individual-level data are not available.

For peer review only

For peer review only - http://bmjopen.bmj.com/site/about/guidelines.xhtml

INTRODUCTION

Although air pollution has detrimental effects on health at all ages[1] there is accumulating evidence that the consequences of early-life exposure may be more important than that occurring at other stages in life. Developing tissues and organs are particularly susceptible due to their high cell proliferation rates in prenatal life and infancy. Postnatally, infants and young children have increased vulnerability due to immature detoxification systems, high infection rates and patterns of behaviour that increase their pollutant exposure.[2] Pregnant mothers exposed to air pollution are more likely to give birth prematurely and have offspring with intrauterine growth retardation and poor growth in infancy.[3 4] Such outcomes are linked with long-term adverse health effects, including increased risks of respiratory and cardiovascular disease.[5] An increasing body of evidence from both the medical and economic literature provide support for the adverse effects of early life pollutant exposure on a variety of health and human capital outcomes in later life. [2,6] This has important public health and policy implications not only in the developed world but also for resource poor countries where open fires and the consequent exposure of mothers and young children to pollutants is widespread.[2 7 8]

In common with many other countries, the UK had high levels of air pollution in the past.[9] Most of this was due to coal use in industry and as a domestic fuel, which gave rise to the "great stinking fogs" that previously characterised London and other major cities.[9] The London smog of 1952 killed an estimated 12,000 people[10] and led to the first UK Clean Air Act being passed in 1956. Following these events, a national survey of smoke and SO_2 concentrations was set up during the 1960s to monitor the progress of pollution control following the Act. From around 1960, there was a progressive reduction in black smoke and SO_2 emissions, with nitrogen oxides, ozone and microparticulates from transport becoming the dominant forms of air pollution.[11]

By the middle of the 20th century, more than 200 million tons of coal were being used per year in England and Wales[12] and yet little is known about the long-term health effects of the combustion of such vast quantities of solid fuel. Studies published in the 1950s and 1960s suggested that there were strong cross-sectional correlations of the amounts of coal used in domestic fires with both childhood respiratory disease [13] and many major causes of adult mortality[14 15] but it is not known if these effects have persisted. The present study is based on a dataset compiled by the Ministry of Fuel and Power that recorded the quantities of different types of solid fuel burnt annually between May 1951 and May 1952 in 1,145 areas of England and Wales. The extent to which exposure to the resulting pollutants may have influenced present day health was assessed by relating these data to mortality rates in the same areas 50-60 years later.

For peer review only - http://bmjopen.bmj.com/site/about/guidelines.xhtml

METHODS

Shortages of coal following the Second World War led to the continuation of a system of rationing and distribution that had been initiated in 1939. This system was controlled by local fuel overseers (LFOs) who were appointed for each local government district (LGD) or group of districts. The Ministry of Fuel and Power published estimates of the quantities of different types of solid fuel burnt annually in each of the 1,145 LFO areas between May 1951 and May 1952 in England and Wales – data were only published for these years.[16] The tables list the consumption of solid fuels classified by the main consumer groups in each LFO area. Domestic (household) supplies included coal, the amounts used in each LFO area based on the number of registered premises, and other solid fuels including coke, briquettes, ovoids and similar patent fuels where consumption was based on merchant's disposals. The annual household coal allowance was higher in the North (2,540 kg) than the South (1,727 kg). Miners' concessionary coal was also included in the estimates. Industrial fuels were predominantly coal and coke and there were separate listings of the quantities of solid fuel used in electricity generation and for carbonization or briquetting plants. The amounts of smokeless fuel (anthracite, dry steam coal, coke, Phurnacite, Coalite and Rexco) used in domestic and industrial premises were recorded separately.

The Office for National Statistics (ONS) provided extracts from all death certificates in England and Wales for each of the current LGDs during 1993 to 2012. This is the period covered by the ninth and tenth revisions of the International Classification of Disease (ICD). The ICD codes used to define causes of death are given in Appendix 1. Mortality rates were calculated using population estimates provided by the ONS for each LGD. The main technical problem was to relate the historical 1,145 LFO areas to the current 342 LGDs, following successive local government reorganisations in 1974, the 1990s, and 2009, together with numerous individual boundary changes. Detailed digital boundaries for the LGDs of England and Wales used in the 1951 census have already been constructed as part of the Great Britain Historical Geographical Information System (GIS).[17 18] As the LFO areas either exactly corresponded to LGDs or were defined as aggregates of them, constructing digital boundary data for LFO areas was straightforward. However, modern LGDs are not necessarily simple aggregates of the LFO areas, so the first stage of the redistricting procedure was to re-allocate the fuel consumption data to the 12,504 Civil Parishes of 1951. Digital boundaries already existed for the parishes, and the re-allocation was based on what proportion each parish's total population, as listed by the 1951 census, was of the overall population of the containing LFO area. The fuel data were then re-allocated to the modern areas by digitally overlaying the two sets of boundaries: whatever proportion of a 1951 parish's area fell within a modern district, that proportion of the parish's fuel consumption was assigned to the district. Air pollution from each source was estimated by calculating the consumption of solid fuel per acre.

For peer review only - http://bmjopen.bmj.com/site/about/guidelines.xhtml

Historically, air pollution was worse in predominantly industrial towns with a high population density; these towns also tended to have large populations of factory workers who left school early and worked in low status occupations.[14] These are important potential confounders in a study of air pollution and disease, as poverty and overcrowding are known to be associated with many of the major causes of mortality. To control for these we used a series of variables extracted from the published reports of the 1951 census: male unemployment; indices of social class; overcrowding, assessed as the proportion of households with more than one person per room; density, the number of people per acre; and educational achievement, indicated by the proportion of employed men in each area who left school before the age of 15 years. Social class was based on the Registrar General's occupation-based system that categorises people into five ordinal groups (I) Professional occupations, (II) Managerial and Technical occupations, (III) Skilled occupations, (IV) Partly-skilled occupations and (V) Unskilled occupations which were combined into a weighted score.[19] The census data were redistricted in the same way as for the fuel. Parallel data were obtained from the 2001 census: unemployment rate, social grade (combined into a weighted score), proportion of households with >1 person/room, persons/hectare, and proportion with no qualifications in each of the 342 LGDs. We also allowed for the impact of current levels of air pollution, which were obtained from published data reporting the modelled annual average concentrations of fine particulate matter ($PM_{2.5}$) in each of the 342 LGDs.[20]

Statistical methods

We studied deaths in people aged between 35 and 74 years, as this was the approximate age range of the generation who were in their first decade of life in 1951-52, and excluded older age groups where the cause of death was less likely to have been ascertained accurately.[21] We calculated the number of deaths expected for each five-year age and sex group within the LGDs by multiplying the number of people in each group by the national age- and sex-specific death rates in 1993-2012. We expressed the number of observed deaths in each area as a percentage of the expected deaths for that group, i.e. as standardised mortality ratios (SMRs). We used Poisson regression to model SMRs, expressing the effect of fuels and other variables on mortality in terms of the hazard ratio (or relative risk) per SD change in the explanatory variable. This weights the analyses appropriately to allow for variation in population size across LGDs. A Fisher-Yates transform[22] was used to standardise pollution and socioeconomic variables. This yielded variables with zero means, unit standard deviations (SDs) and symmetrical distributions, allowing direct comparison of the strength of associations with these variables.

Patient and Public Involvement

No patients or public were involved in this study.

For peer review only - http://bmjopen.bmj.com/site/about/guidelines.xhtml

RESULTS

There were a total of 3,535,136 deaths in England and Wales during the 20-year period from 1993 to 2012 in the 35- to 74-yr age-group. Mortality rates (SMR) in the 342 LGDs ranged from 64.7 in East Dorset to 153.1 in Manchester. Table 1 shows the different uses of solid fuel during 1951/2 in England and Wales. Of the over 200 million tons distributed, 37.6% was used in industrial processes, 16.6% in electricity generation and 27.5% in carbonization or briquetting processes. Only 18.3% was used domestically. Smokeless fuel accounted for approximately 32.1% of industrial and 14.3% of domestic consumption. Figure 1 is a map of England and Wales showing the quintiles of domestic fuel consumed per acre. This was generally low in rural areas but was greatest in London and in the former industrial areas of the UK (South Wales, the Midlands, Lancashire, the West Riding of Yorkshire and Tyneside). The mean current $PM_{2.5}$ concentrations in the 342 areas was 9.48 (SD 1.67) $\mu g/m^3$ and ranged from 5.5 in Gwynedd (Wales) to 14.9 $\mu g/m^3$ in the City of Westminster, London.

Table 2 shows the relationship between the various sources of fuel burnt in 1951/2 (excluding smokeless fuels) and subsequent all-cause mortality or mortality due to respiratory, cardiovascular disease and cancer in the 342 areas. The influence of the 1951 census socioeconomic indicators (social class, education, crowding, density and unemployment) are also shown. In univariate analyses, all variables were significantly associated with both all-cause and cause-specific mortality rates although the effects of domestic and industrial consumption tended to be most strongly related to the mortality outcomes. Multivariate analyses were carried out to evaluate the relative effects of fuel usage and the major confounding variables derived from the 1951 census. This analysis shows that domestic consumption was strongly and significantly associated with both all-cause and cause-specific mortality rates, an effect which was markedly stronger than the other sources of fuel. Smokeless fuels represented less than 15% of the total fuels used (Table 1) and in separate regression analyses had no statistically significant independent effect on mortality rates. (Data not shown)

There was a very high correlation (r=0.97) between domestic consumption and population density, resulting in unacceptable collinearity in regression analyses that contained both variables. This occurred because both had the same denominator, the area of the LGD. To allow the influence of both of these factors to be modelled statistically, we created two further variables: the sum of and the difference between domestic use and density. These variables were uncorrelated (r=0.0). These were entered into the regression models in Table 2, which shows both the unadjusted coefficients and the coefficients adjusted for all other variables (Table 2 lower panel). The positive regression coefficients for the sum variable show that the joint effects of domestic use and density on all four causes of mortality were powerful. However, associations with the difference variable suggest that the influence of domestic use was stronger than that of density.

For peer review only - http://bmjopen.bmj.com/site/about/guidelines.xhtml

Table 3 shows the relationship between current socioeconomic indicators or microparticulate air pollution and all-cause or cause-specific mortality. The lower section of Table 3 shows that the effect of domestic coal usage is attenuated by successive adjustments for socioeconomic indicators from the 1951 census, for indicators from the 2001 census together with particulate exposure and for both of these sets of factors. However, in all cases the association with domestic coal consumption remained strong and statistically significant.

The effects of the potential confounders were tested singly and in combination. There was no evidence of non-linear relationships. Figure 2 shows the associations between quartiles of domestic fuel consumption and all-cause mortality or the major mortality categories. These associations were progressive and remained strong and statistically significant for each cause group after adjustment for social class, education, crowding, unemployment in 1951, and for current socioeconomic indicators and $PM_{2.5}$ concentrations. They were present in both genders and were similar during the ICD 9 and ICD 10 periods.

Figure 3 shows the strength of the geographical correlations between domestic consumption and current mortality according to successive birth cohorts. For all-cause and respiratory mortality, the strongest associations were observed among those born in 1952/3 suggesting that exposure around the time of birth had the greatest effect on mortality. For cardiovascular and cancer mortality, the peak associations were more blunted, suggesting that exposure over a wider age-range was associated with subsequent mortality.

Tables 4 & 5 show the relationship between domestic fuel consumption and specific causes of mortality. As before, the data are shown before and after adjustment for past and current socioeconomic indicators and current $PM_{2.5}$ levels. The respiratory conditions responsible for most deaths, chronic obstructive pulmonary disease and pneumonia, were strongly and consistently associated with domestic fuel consumption while associations with asthma mortality were somewhat weaker. However, the largest relative risks were with tuberculosis. While all four major causes of cardiovascular mortality were significantly associated with domestic consumption, the strongest relationship was with rheumatic heart disease.

The data on associations of domestic fuel use with mortality from specific cancers were striking (Table 5). There were strong relationships with epithelial cancers; lip, oral cavity and pharynx (adjusted hazard ratio, 1.207); laryngeal cancers (1.263) and lung (1.126). The upper gastrointestinal cancers; oesophagus (1.088), stomach (1.117) and liver (1.053), were more strongly associated with domestic pollution than the lower gastrointestinal cancers. In contrast, the major urinary, reproductive and haematological

For peer review only - http://bmjopen.bmj.com/site/about/guidelines.xhtml

cancers showed no increase in risk, with the exception of cervical cancer (1.131). Some cancers, for example melanoma and brain, were associated with a somewhat reduced relative risk.

DISCUSSION

Areas of the UK that had high domestic consumption of coal and related non-smokeless solid fuels in 1951/52 now have raised mortality from a wide variety of causes, including cardiovascular and respiratory diseases and certain cancers. The correlations are strong, statistically significant and independent of all available variables that might be considered major confounders, whether assessed in 1951 or currently, including social class, level of education, overcrowding, and unemployment.

Very few comparable data exist in the literature, particularly with such a long follow-up period. Our findings accord with an increasing body of evidence that early life exposure to air pollution has detrimental long-term health effects.[6 15 23] In particular, two recent studies demonstrated important findings. In the first, children exposed to the great London Smog of 1952 were found to have greater risk of asthma in adulthood, compared to unexposed children.[24] In the second, which was based on the ONS longitudinal study, air pollution concentrations at the site of residence, assessed every 10 years from 1971, were associated with increased total, respiratory and cardiovascular mortality.[25] Our conclusions also accord with historical studies of coal use and mortality in Britain, which estimated that each SD increase in coal use raised mortality by 5-15% in infants and 5% in adults.[26]

Our study necessarily depended on published crude estimates of coal consumption as an index of domestic air pollution. We contend that is very reasonable to assume that the quantity of coal burnt relates to the amount of pollution emitted although local factors such as climate and wind strength would also have a major influence. The consumption measures in our study were shown to correlate with subsequent measurements of pollution in the UK in the early 1960s,[13] when coal was still a major fuel and source of pollution. This approach is also supported by a number of studies carried out over the last 60 years, showing correlations with diverse health outcomes including reduced early growth,[3] chronic bronchitis and lung cancer.[13-15] While the consumption data come from a short time-period between 1951 and 1952, they are likely to reflect smoke emission over many years. UK coal production was fairly constant during the late 1940s, declined a little during the 1950s and only fell dramatically during the 1960s and subsequent decades.[12] In the post-war years, economic necessity drove the use of low-grade, bituminous domestic coal while better-quality "hard" coals were exported. A typical, inefficient domestic grate burning low-grade coal would have produced lots of smoke, rich in a wide variety of potentially toxic compounds, including heavy metals, sulphur and complex mixtures of aliphatic and aromatic hydrocarbons.[27] Confirmation of this can be found in the lung tissue from autopsies of people exposed to the 1952 London smog, which was shown to contain both ultrafine carbonaceous and metal

For peer review only - http://bmjopen.bmj.com/site/about/guidelines.xhtml

particulate matter.[28] Children would have been exposed both in the home and in their local environment although it is not clear from our data which of these would have been the most important source. However, our study suggests that it was domestic fuel consumption that had the greatest long-term adverse health effects, with industrial pollution or pollution from power stations or other sources causing relatively smaller effects (Table 2). The probable explanation for this is that most industrial and power station pollution was vented through tall chimneys, decreasing ground-level pollution in approximate proportion to the inverse square of chimney height.[29]

Current levels of pollution in the 342 local areas were based on ambient levels of fine particulate matter ($PM_{2.5}$) because large cohort studies have shown that this measure correlates best with long-term health effects of pollution.[30-32] The effects of $PM_{2.5}$ on current mortality (Table 3) in the present study are consistent with previously published estimates,[20] but importantly the mortality correlations with coal consumption persisted after adjustment for this measure of current pollution.

Our data do not allow determination of the age at which children were most vulnerable to the effects of pollution although the cohort-based analysis (Figure 3) suggests that exposures around the time of birth or infancy are likely to have been most important especially for all-cause and respiratory mortality. We can also only speculate on the mechanism by which air pollution arising from coal consumption, predominantly in a domestic setting, might increase the risk of mortality. There may have been direct mutagenic effects, especially in relation to the increased cancer risk. Another possibility is that the pollutants triggered mechanisms that selected alternative developmental pathways in the young, perhaps through epigenetic modification of gene expression.[33]

Elevated relative risks were observed for respiratory diseases, especially COPD, asthma and pneumonia, (Table 4) which is consistent with past evidence that black smoke exposure is a risk factor for these conditions.[14 15 23] The strongest association we found, however, was with tuberculosis, which is less supported by existing literature. A recent systematic review and meta-analysis concluded that the evidence for such an association was inconclusive but it was reliant on case-control and cross-sectional studies.[34] The findings in the present study raise the possibility that early exposure to air pollution could be a critical factor in determining susceptibility to tuberculosis although other factors such as population density cannot be completely excluded. Cardiovascular disease was also found to be strongly associated with domestic fuel consumption, which accords with earlier UK findings,[15 23] the Harvard Six Cities study,[31] and a multicentre study of European Cohorts.[35] The strength of the association was similar with ischaemic heart disease and stroke. The association with rheumatic heart disease was strong and confirms the findings of a previous study based on a subset of large Country Boroughs in England and Wales, and further supports the suggestion that an increase in susceptibility to infectious diseases is linked with exposure to air pollution in infancy or early childhood.[36] An additional finding was a strong

For peer review only - http://bmjopen.bmj.com/site/about/guidelines.xhtml

association with hypertension, which is supported by a meta-analysis that showed both short-term and long-term exposure to some air pollutants increases the risk of hypertension.[37] The data on cancers (Table 5) show wide variation in the level of associated risk. They show that the strongest associations are observed with epithelial cancers of the respiratory system (lip, oral cavity and pharynx; larynx; trachea and lung) and the upper gastrointestinal system (stomach and liver). The major reproductive, urinary and haematological cancers were not positively associated with domestic pollution with the exception of cervical cancer, for which there is evidence that cigarette smoking is an important co-factor.[38] Malignant melanoma had a lower relative risk in the areas with high consumption possibly because of reduced sunlight exposure. Some cancers showed small reductions in relative risk, for example brain cancer, and although the reasons are not clear, this may be due to residual confounding, which is evident because of the very large numbers of deaths and high sensitivity of this analysis.

There are a number of potential limitations to our study. Because neighbouring geographical areas tend to have similar levels of coal consumption, spatial auto-correlation may lead to lead to confidence intervals that are narrower than they should be. However, an analysis evaluating the influence of spatial autocorrelation (described in detail in Appendix 2) suggest that this is not a serious problem in the coal consumption data. Calculations incorporating the autocorrelation term into regression analyses increase the very narrow confidence intervals, for example in Table 2, by 7.1%.

An inevitable issue with evaluating the association of early exposures with causes of late-life mortality is the long latency between, in this case, the measurements of coal consumption and the outcomes. It could be argued that internal migration might have weakened the associations that we observed as selective migration has been proposed as an explanation for geographical variations in mortality. This is supported by some studies[39] but not others.[40 41] Migration is complex as, while migrants are generally healthier, those moving short distances tend to have higher mortality than those moving long distances.[42 43] Although we do not have migration data, most migration during the time period of this study occurred over short distances, with only a small minority migrating significant distances.[44] A more recent study of migration based on the 2001 census, while documenting the complex age and social class influences on migration, suggested that the declining industrial areas of South Wales, Yorkshire, Greater Manchester and Lancashire, and the North-East of England, which were the areas that had high coal consumption, had in-migration and out-migration levels in all age-groups that were far below the National average.[45] In addition, our analysis allows for the factors that tend to drive migration such as education and socioeconomic status.[46] Finally, it is difficult to see how selective migration could explain the specific links between coal consumption and some conditions and not others (Table 5).

Any undetected bias in our observational study would be most likely to be due to confounding from an unknown source. However, a sensitivity analyses suggested that such a variable would have to correlate

For peer review only - http://bmjopen.bmj.com/site/about/guidelines.xhtml

very strongly (r >0.5) with both domestic air pollution and respiratory disease, for example, to confound the relationships we have observed. There are no obvious candidates for such a confounder that were not addressed by our analysis, apart from air pollutants "Air Toxics" from sources other than the combustion of coal and other solid fuels, measurements of which are unavailable for the early 1950s. As many of these would have been geographically localised and specific to certain industries it is difficult to see how they could be confounders especially as our analyses incorporate adjustment for socioeconomic factors (eg occupation/urbanisation) that would reflect differential exposure to these substances. In addition we have examined the effects of the major confounding variables that were available in the 1951 and 2001 census and that are likely to be related to both residence in an area of high pollution and to the various outcomes that we have studied. The 1951 census contained several additional measures of household amenities, which were also examined in regression analyses. We found that these did not explain any further variance in our analyses beyond that due to the confounding variables that we used in the final analyses. Adjusting the risk estimates associated with domestic consumption for social class, density, unemployment and education reduced their magnitude but they still remained strongly significant (Table 3 and Figure 2). However, it could be argued that some of these variables are so closely related to the measures of pollution that the regression-based adjustment has resulted in conservative risk estimates. Unsurprisingly, population density was highly correlated with domestic fuel use because fuel was rationed on a per household basis. In our analyses based on a published technique,[47] we were able to show that pollution had a more marked effect than population density but the close relationship between these variables prevented us from using this in the adjusted regression models and, as a result, we cannot confidently exclude the influence of early population density. However, we would also argue that, in this context, density (population per unit area) may be a less important confounder than overcrowding (proportion of households with >1 person per room). There is considerable observational evidence linking overcrowding with physical health, especially in adults[48] and in a review of the environmental factors most associated with health outcomes, Evans and Kantrowitz concluded that areal indices of density were less important than overcrowding in determining the health outcomes associated with high population density.[49] Finally, our data showing linear relationships between fuel usage and mortality (Figure 2) are unlikely to be driven by the influence of density on pathogen transmission where saturation effects would be expected.[50] Although we did not have data on the prevalence of tobacco smoking, we do not think that this is likely to have seriously confounded our results. In the early 1950s smoking was a social norm (in 1951 78% of men used tobacco), smoking is associated with low socioeconomic status,[51] which was included in our analyses, and standardisation of the data for lung cancer rates as a proxy for smoking did not explain the correlations we observed.

For peer review only - http://bmjopen.bmj.com/site/about/guidelines.xhtml

Conclusions

Although this is an ecological study based on geographical correlations, the results raise the possibility that domestic air pollution, experienced over 60 years ago by young children, affects human health now, by increasing mortality from a wide variety of diseases. The effect of early life exposure was much stronger than that of current pollution assessed by concentrations of microparticulates. Importantly, the findings raise the possibility that the health effects of air pollution have been underestimated as most studies do not have data on early life exposure, where vulnerability may be greatest, and even the effects of the lower levels of pollution experienced by current Western populations appears to have been under-appreciated. Indeed, a recent mortality study based on the Medicare population suggests that a significant disease burden is associated with pollutant levels that are below current statutory standards.[52] The evidence that air pollution has been having a major detrimental impact on health for decades and continues to do so underlines the importance of routine environmental tracking and surveillance systems in detecting and avoiding the harmful health effects of environmental pollution. The data presented in this analysis also have implications for the long-term health of the populations of countries that still depend on large amounts of coal for their domestic markets. This includes newly industrialized countries such as India or China, where coal is a major energy source, and resource-poor countries, where indoor air pollution from cook stoves results in heavy exposure of women and young children to pollution.[7]

Acknowledgments: The authors are grateful to Laura Todd and her colleagues at the UK Office for National Statistics for their help in providing the mortality data. We also thank David Coggon for helpful comments on the manuscript.

For peer review only

For peer review only - http://bmjopen.bmj.com/site/about/guidelines.xhtml

REFERENCES

1. Landrigan PJ, Fuller R, Acosta NJR, et al. The Lancet Commission on pollution and health. *Lancet* 2017;http://dx.doi.org/10.1016/S0140-6736(17)32345-0 [published Online First: Oct 2017]
2. WHO. Effects of Air Pollution on Children's Health and Development. European Centre for Environment and Health. Bonn: WHO, 2005:1-184.
3. Amegah AK, Quansah R, Jaakkola JJ. Household air pollution from solid fuel use and risk of adverse pregnancy outcomes: a systematic review and meta-analysis of the empirical evidence. *PloS one* 2014;9(12):e113920. doi: 10.1371/journal.pone.0113920
4. Bobak M, Richards M, Wadsworth M. Relation between children's height and outdoor air pollution from coal-burning sources in the British 1946 birth cohort. *IntArchOccupEnvironHealth* 2004;77(6):383-86. doi: 10.1007/s00420-004-0522-5 [doi]
5. Barker DJP. The Wellcome Foundation Lecture, 1994. The fetal origins of adult disease. *Proc Roy Soc Lond* 1995;262:37-43.
6. Currie J, Zivin JG, Mullins J, et al. What Do We Know About Short- and Long-Term Effects of Early-Life Exposure to Pollution? *Annu Rev Resour Econ* 2014;6:217-47. doi: 10.1146/annurev-resource-100913-012610
7. Mortimer K, Gordon SB, Jindal SK, et al. Household air pollution is a major avoidable risk factor for cardiorespiratory disease. *Chest* 2012;142(5):1308-15. doi: 1389935 [pii];10.1378/chest.12-1596 [doi]
8. UNICEF. Clear the air for children. New York: UNICEF, 2016:1-97.
9. Brimblecombe P. The Big Smoke: Methuen 1987:1-204.
10. Bell ML, Davis DL, Fletcher T. A retrospective assessment of mortality from the London smog episode of 1952: the role of influenza and pollution. *EnvironHealth Perspect* 2004;112(1):6-8.
11. Ayres JG. Trends in air quality in the UK. *Allergy* 1997;52(38 Suppl):7-13.
12. Department for Business Energy & Industrial Strategy. Historical coal data: coal production, availability and consumption 1853 to 2015. www.gov.uk/government/statistical-data-sets/historical-coal-data-coal-production-availability-and-consumption-1853-to-2011
13. Douglas JWB, Waller RE. Air Pollution and respiratory infection in children. *Br J Prev Soc Med* 1966;20:1-8.
14. Daly C. Air pollution and causes of death. *Br J Prev Soc Med* 1959;13(1):14-27.
15. Gardner MJ, Crawford MD, Morris JN. Patterns of mortality in middle and early old age in the county boroughs of England and Wales. *Br J Prev Soc Med* 1969;23(3):133-40.
16. Ministry of Fuel and Power. Statistical Digest, 1954. London1955.
17. Gregory IN, Southall HR. Putting the Past in Its Place: the Great Britain Historical GIS In: Carver S, ed. Innovations in GIS 5: Taylor & Francis 1998:210-21.
18. Southall H. Rebuilding the Great Britain Historical GIS, Part 2 A Geo-Spatial Ontology of Administrative Units. *Hist Method* 2012;45(3):119-34. doi: 10.1080/01615440.2012.664101
19. Donkin A, Goldblatt P, Lynch K. Inequalities in life expectancy by social class, 1972-1999. *Health Statistics Quarlerly* 2002;15:5-15.
20. Gowers AM, Miller BG, Stedman JR. Estimating local mortality burdens associated with particulate air pollution. In: England PH, ed. London: Public Health England, 2014:1-40.
21. Paterson DA, Dorovitch MI, Farquhar DL, et al. Prospective study of necropsy audit of geriatric inpatient deaths. *J Clin Pathol* 1992;45(7):575-8.
22. Fisher RA, Yates F. Statistical tables for biological, agricultural, and medical research. Edinburgh: Oliver & Boyd.
23. Elliott P, Shaddick G, Wakefield JC, et al. Long-term associations of outdoor air pollution with mortality in Great Britain. *Thorax* 2007;62(12):1088-94. doi: 10.1136/thx.2006.076851
24. Bharadwaj P, Graff Zivin J, Mullins JT, et al. Early Life Exposure to the Great Smog of 1952 and the Development of Asthma. *American journal of respiratory and critical care medicine* 2016 doi: 10.1164/rccm.201603-0451OC
25. Hansell A, Ghosh RE, Blangiardo M, et al. Historic air pollution exposure and long-term mortality risks in England and Wales: prospective longitudinal cohort study. *Thorax* 2016;71(4):330-38. doi: 10.1136/thoraxjnl-2015-207111

For peer review only - http://bmjopen.bmj.com/site/about/guidelines.xhtml

26. Beach B, Walker Hanlon W. Coal smoke and mortality in an early industrial economy. *The Economic Journal* 2016;DOI: 10.1111/ecoj.12522:60. [published Online First: 18 Nov 2017]
27. IARC. Indoor emissions from household combustion of coal. Lyon, France: WHO, 2012:515-38.
28. Hunt A, Abraham JL, Judson B, et al. Toxicologic and epidemiologic clues from the characterization of the 1952 London smog fine particulate matter in archival autopsy lung tissues. *Environmental health perspectives* 2003;111(9):1209-14.
29. Williams ML. Atmospheric dispersal of pollutants and the modelling of air pollution. In: RM H, ed. Pollution, causes, effects and control. 5th ed. London: Royal Society of Chemistry 2015:225-43.
30. Dockery DW, Pope CA, 3rd, Xu X, et al. An association between air pollution and mortality in six U.S. cities. *N Engl J Med* 1993;329(24):1753-9. doi: 10.1056/NEJM199312093292401
31. Laden F, Schwartz J, Speizer FE, et al. Reduction in fine particulate air pollution and mortality: Extended follow-up of the Harvard Six Cities study. *American journal of respiratory and critical care medicine* 2006;173(6):667-72. doi: 10.1164/rccm.200503-443OC
32. Pope CA, 3rd, Burnett RT, Thun MJ, et al. Lung cancer, cardiopulmonary mortality, and long-term exposure to fine particulate air pollution. *JAMA* 2002;287(9):1132-41.
33. Godfrey KM, Costello PM, Lillycrop KA. The developmental environment, epigenetic biomarkers and long-term health. *Journal of developmental origins of health and disease* 2015;6(5):399-406. doi: 10.1017/S204017441500121X
34. Lin HH, Suk CW, Lo HL, et al. Indoor air pollution from solid fuel and tuberculosis: a systematic review and meta-analysis. *The international journal of tuberculosis and lung disease : the official journal of the International Union against Tuberculosis and Lung Disease* 2014;18(5):613-21. doi: 10.5588/ijtld.13.0765
35. Wolf K, Stafoggia M, Cesaroni G, et al. Long-term Exposure to Particulate Matter Constituents and the Incidence of Coronary Events in 11 European Cohorts. *Epidemiology* 2015;26(4):565-74. doi: 10.1097/EDE.0000000000000300
36. Phillips DIW, Osmond C, Williams ML, et al. Air pollution in early life and adult mortality from chronic rheumatic heart disease. *Int J Epidemiol* 2017;46(4):1107-14. doi: 10.1093/ije/dyw249
37. Cai Y, Zhang B, Ke W, et al. Associations of Short-Term and Long-Term Exposure to Ambient Air Pollutants With Hypertension: A Systematic Review and Meta-Analysis. *Hypertension* 2016;68(1):62-70. doi: 10.1161/HYPERTENSIONAHA.116.07218
38. Trimble CL, Genkinger JM, Burke AE, et al. Active and passive cigarette smoking and the risk of cervical neoplasia. *Obstet Gynecol* 2005;105(1):174-81. doi: 10.1097/01.AOG.0000148268.43584.03
39. Norman P, Boyle P, Rees P. Selective migration, health and deprivation: a longitudinal analysis. *Soc Sci Med* 2005;60(12):2755-71. doi: 10.1016/j.socscimed.2004.11.008
40. Strachan DP, Leon DA, Dodgeon B. Mortality from cardiovascular disease among interregional migrants in England and Wales. *BMJ* 1995;310(6977):423-7.
41. Osmond C, Barker DJ, Slattery JM. Risk of death from cardiovascular disease and chronic bronchitis determined by place of birth in England and Wales. *JEpidemiolCommunity Health* 1990;44(2):139-41.
42. Fox AJ, Goldblatt PO. Socioeconomic differences in mortality. *Population Trends* 1982;27:8-13.
43. Boyle P. Population geography: migration and inequalities in mortality and morbidity. *Progress in Human Geography* 2004;28(6):767-76.
44. Pooley CG, Turnbull J. Migration trends in British rural areas from the 18th to the 20th centuries. *IntJPopulGeogr* 1996;2(3):215-37. doi: 10.1002/(SICI)1099-1220(199609)2:3<215::AID-IJPG35>3.0.CO;2-N [doi]
45. Dennett AR. Understanding Internal Migration in Britain at the Start of the 21st Century. University of Leeds, 2010.
46. Champion T. Migration and British Cities in the 1990s. *National Institute Economic Review* 1999;170:60-76.
47. King MD, Fountain C, Dakhlallah D, et al. Estimated autism risk and older reproductive age. *Am J Public Health* 2009;99(9):1673-9. doi: 10.2105/AJPH.2008.149021
48. Minister' OotDP. The Impact of Overcrowding on Health and Education: A review of the Evidence and Literature. London: , 2004:1-38.
49. Evans GW, Kantrowitz E. Socioeconomic status and health: The potential role of environmental risk exposure. *Annu Rev Publ Health* 2002;23:303-31. doi: DOI 10.1146/annurev.publhealth.23.112001.112349

For peer review only - http://bmjopen.bmj.com/site/about/guidelines.xhtml

50. Hu H, Nigmatulina K, Eckhoff P. The scaling of contact rates with population density for the infectious disease models. *Math Biosci* 2013;244(2):125-34. doi: 10.1016/j.mbs.2013.04.013
51. Kleinschmidt I, Hills M, Elliott P. Smoking behaviour can be predicted by neighbourhood deprivation measures. *J Epidemiol Community Health* 1995;49 Suppl 2:S72-7.
52. Di Q, Wang Y, Zanobetti A, et al. Air Pollution and Mortality in the Medicare Population. *N Engl J Med* 2017;376(26):2513-22. doi: 10.1056/NEJMoa1702747

For peer review only

For peer review only - http://bmjopen.bmj.com/site/about/guidelines.xhtml

Table 1: Fuel consumption in England and Wales 1951/1952.

	Total annual consumption (tons x 1000)	Annual consumption per acre in the 342 LGDs (tons)	
		Median	Quartiles
Total Domestic	36,783	1.00	0.29,4.38
(Smokeless)	5,247	0.17	0.05,0.63
Total industrial	75,597	1.17	0.23,5.58
(Smokeless)	24,294	0.47	0.12,2.07
Carbonisation & briquetting	55,122	0.63	0.10,5.36
Electricity generation	33,304	0.07	0.00,1.85
Total	200,806	4.21	0.82,21.38

For peer review only - http://bmjopen.bmj.com/site/about/guidelines.xhtml

Table 2: The effect of fuel consumed in 1951/52 and socioeconomic indicators derived from the 1951 census on current causes of mortality in the 342 LGDs in England and Wales.

	Relative risk of mortality per SD change in fuel usage or socioeconomic indicator (95% CI)			
	**All Cause**	**Respiratory** (ICD9: 460 to 519, ICD10: J00 to J99)	**Cardiovascular** (ICD9: 390 to 459 ICD10: I00 to I99)	**Cancer** (ICD9: 140-208, ICD10: C00 to C97)
**Univariate analyses**				
Fuel consumed 1951/2				
Domestic (ex. smokeless)	1.124(1.123 to 1.126)	1.238(1.234 to 1.242)	1.138(1.136 to 1.140)	1.073(1.071 to 1.075)
Industrial (ex. smokeless)	1.129(1.128 to 1.131)	1.244(1.239 to 1.248)	1.152(1.150 to 1.154)	1.080(1.079 to 1.082)
Carbonization	1.120(1.119 to 1.122)	1.225(1.221 to 1.230)	1.135(1.133 to 1.137)	1.073(1.071 to 1.074)
Electricity generation	1.096(1.095 to 1.097)	1.181(1.177 to 1.185)	1.110(1.107 to 1.112)	1.054(1.052 to 1.056)
1951 census				
Social class score[+]	1.117(1.115 to 1.118)	1.190(1.186 to 1.194)	1.144(1.142 to 1.146)	1.087(1.085 to 1.089)
Low educational level	1.093(1.092 to 1.094)	1.154(1.150 to 1.158)	1.125(1.123 to 1.127)	1.064(1.062 to 1.066)
Crowding	1.106(1.105 to 1.108)	1.179(1.175 to 1.183)	1.126(1.124 to 1.128)	1.075(1.073 to 1.077)
Density	1.105(1.104 to 1.106)	1.206(1.202 to 1.211)	1.113(1.111 to 1.115)	1.059(1.057 to 1.061)
Unemployment	1.074(1.073 to 1.076)	1.113(1.109 to 1.117)	1.077(1.075 to 1.079)	1.055(1.053 to 1.057)
**Multivariate analysis**				
Fuel consumed 1951/2				
Domestic (ex. smokeless)	1.061(1.058 to 1.064)	1.127(1.118 to 1.137)	1.051(1.046 to 1.056)	1.027(1.023 to 1.031)
Industrial (ex. smokeless)	1.008(1.006 to 1.011)	1.031(1.023 to 1.039)	1.020(1.016 to 1.025)	1.011(1.007 to 1.015)
Carbonization	1.029(1.027 to 1.030)	1.044(1.038 to 1.050)	1.029(1.026 to 1.033)	1.019(1.016 to 1.022)
Electricity generation	1.002(1.000 to 1.003)	0.999(0.994 to 1.004)	1.005(1.002 to 1.008)	0.995(0.992 to 0.997)
1951 census				
Social Class	1.062(1.060 to 1.064)	1.094 (1.087 to 1.101)	1.058(1.054 to 1.062)	1.054(1.051 to 1.058)
Education	1.021(1.019 to 1.022)	1.027(1.021 to 1.032)	1.045(1.042 to 1.048)	1.007(1.005 to 1.010)
Crowding	1.005(1.004 to 1.007)	1.001(0.996 to 1.006)	1.012(1.009 to 1.014)	1.007(1.005 to 1.010)
Unemployment	1.032(1.031 to 1.034)	1.037(1.032 to 1.042)	1.036(1.034 to 1.039)	1.027(1.025 to 1.030)

For peer review only - http://bmjopen.bmj.com/site/about/guidelines.xhtml

Contd.

**Combined effects analysis of domestic usage and density**				
Unadjusted				
Sum	1.112(1.111 to 1.113)	1.219(1.215 to 1.223)	1.122(1.120 to 1.124)	1.064(1.062 to 1.066)
Difference (domestic – density)	1.074(1.073 to 1.076)	1.117(1.113 to 1.121)	1.095(1.093 to 1.097)	1.052(1.050 to 1.054)
Adjusted*				
Sum	1.054(1.051 to 1.056)	1.116(1.107 to 1.126)	1.042(1.037 to 1.047)	1.022(1.017 to 1.026)
Difference (domestic – density)	1.031(1.029 to 1.032)	1.048(1.044 to 1.053)	1.036(1.034 to 1.038)	1.018(1.016 to 1.020)

*Adjusted for social class, education, crowding, unemployment, industrial usage, and fuel used in carbonization & electricity generation.
+See methods

For peer review only

For peer review only - http://bmjopen.bmj.com/site/about/guidelines.xhtml

Table 3: The effect of socioeconomic indicators derived from the 2001 census and current $PM_{2.5}$ concentrations on current causes of mortality in the 342 LGDs in England and Wales together with multivariate models evaluating the association between domestic coal usage and mortality following adjustment for the 1951 and 2001 socioeconomic indicators and current $PM_{2.5}$ exposure.

	Relative risk of mortality per SD change in socioeconomic indicator, particulate or fuel usage (95%CI)			
	**All Cause**	**Respiratory** (ICD9: 460 to 519, ICD10: J00 to J99)	**Cardiovascular** (ICD9: 390 to 459 ICD10: I00 to I99)	**Cancer** (ICD9: 140-208, ICD10: C00 to C97)
**Univariate analyses**				
2001 census				
Social class score[+]	1.139(1.137 to1.140)	1.221(1.217 to 1.226)	1.170(1.168 to 1.173)	1.098(1.096 to 1.100)
Low educational level	1.122(1.121 to 1.123)	1.195(1.191 to 1.199)	1.157(1.155 to 1.159)	1.087(1.085 to 1.089)
Crowding	1.101(1.099 to 1.102)	1.196(1.192 to 1.201)	1.117(1.115 to 1.119)	1.052(1.051 to 1.054)
Density	1.101(1.100 to 1.102)	1.196(1.192 to 1.200)	1.107(1.104 to 1.109)	1.059(1.057 to 1.061)
Unemployment	1.157(1.156 to 1.158)	1.270(1.266 to 1.275)	1.184(1.182 to 1.186)	1.099(1.097 to 1.101)
Current $PM_{2.5}$	1.027(1.026 to 1.028)	1.076(1.073 to 1.080)	1.028(1.027 to 1.030)	1.005(1.003 to 1.006)
**Multivariate analyses**				
Model 1 (effect of domestic usage allowing for 1951 socioeconomic indicators)	1.094(1.092 to 1.095)	1.195(1.190 to 1.200)	1.097 (1.095 to 1.100)	1.038 (1.033 to 1.043)
Model 2 (effect of domestic usage allowing for 2001 socioeconomic indicators and current $PM_{2.5}$)	1.081(1.078 to 1.084)	1.179(1.167 to 1.190)	1.088(1.082 to 1.093)	1.038(1.033 to 1.043)
Model 3 (effect of domestic usage allowing for 1951 and 2001 socioeconomic indicators and current $PM_{2.5}$)	1.084(1.080 to 1.087)	1.189(1.177 to 1.201)	1.088(1.083 to 1.094)	1.037 (1.032 to 1.042)

[+]See methods

For peer review only - http://bmjopen.bmj.com/site/about/guidelines.xhtml

Table 4: Association between domestic fuel usage and major causes of non-cancer mortality among men and women aged 35-74 in the 342 areas.

	Relative risk per SD increase in domestic usage			
	Unadjusted (95%CI)		Adjusted* (95% CI)	
Respiratory				
Chronic obstructive pulmonary disease	1.253	1.245 to 1.260	1.142	1.122 to 1.163
Asthma	1.199	1.176 to 1.223	1.093	1.032 to 1.158
Pneumonia	1.262	1.254 to 1.269	1.227	1.205 to 1.250
Tuberculosis	1.734	1.676 to 1.793	1.298	1.167 to 1.444
Cardiovascular				
Ischaemic heart disease	1.141	1.138 to 1.143	1.102	1.095 to 1.110
Rheumatic heart disease	1.209	1.184 to 1.234	1.240	1.166 to 1.318
Stroke	1.137	1.133 to1.141	1.114	1.101 to 1.127
Hypertension	1.204	1.196 to 1.212	1.112	1.089 to 1.135

*Adjusted for 1951 and 2001 socioeconomic indicators and current $PM_{2.5}$ exposure.

For peer review only - http://bmjopen.bmj.com/site/about/guidelines.xhtml

Table 5: Association between domestic fuel usage and cancer mortality in the 342 areas.

Site	Relative risk per SD increase in domestic usage			
	Unadjusted (95%CI)		Adjusted* (95% CI)	
Lip, oral cavity and pharynx	1.220	1.204 to 1.236	1.207	1.161 to 1.255
Oesophagus	1.038	1.030 to 1.046	1.088	1.064 to 1.112
Stomach	1.171	1.161 to 1.182	1.117	1.088 to 1.147
Colon	0.996	0.989 to 1.002	1.027	1.007 to 1.048
Rectosigmoid junction, rectum and anus	1.073	1.063 to 1.082	1.039	1.012 to 1.066
Liver, gall bladder & bile ducts	1.193	1.179 to 1.206	1.053	1.019 to 1.089
Pancreas	1.022	1.014 to 1.030	0.977	0.956 to 0.999
Larynx	1.347	1.318 to 1.376	1.264	1.185 to 1.348
Trachea, bronchus and lung	1.195	1.190 to 1.199	1.126	1.115 to 1.137
Malignant melanoma	0.885	0.873 to 0.898	0.889	0.854 to 0.924
Breast	0.990	0.984 to 0.995	0.958	0.943 to 0.974
cervix	1.155	1.135 to 1.175	1.131	1.074 to 1.190
Uterus	1.032	1.015 to 1.049	0.930	0.886 to 0.975
Ovary	0.968	0.959 to 0.976	0.986	0.960 to 1.012
Prostate	0.989	0.981 to 0.998	0.962	0.939 to 0.986
Kidney	1.000	0.989 to1.010	1.003	0.973 to 1.035
Bladder	1.056	1.044 to 1.068	1.024	0.991 to 1.057
Brain , eye & CNS	0.952	0.943 to 0.961	0.962	0.936 to 0.988
Hodgkin's disease	1.030	0.994 to 1.067	0.919	0.829 to 1.018
Non-Hodgkin's lymphoma	1.024	1.014 to 1.034	0.952	0.925 to 0.979
Multiple myeloma	0.999	0.986 to 1.013	0.941	0.905 to 0.978
Leukaemia	1.008	0.998 to 1.019	0.961	0.931 to 0.991

*Adjusted for 1951 and 2001 socioeconomic indicators and current $PM_{2.5}$ exposure.

For peer review only - http://bmjopen.bmj.com/site/about/guidelines.xhtml

For peer review only

Figure 1: Domestic fuel consumption in England and Wales, 1951-52.

Figure 2: Association between quartiles of domestic fuel consumption (ex smokeless) and both all-cause and the major causes of mortality in men and women aged 35-74 in the 342 LGDs. (●unadjusted, ○ adjusted for socioeconomic indicators in 1951 and 2001 and current $PM_{2.5.}$; errors are 95% CI)

Figure 3: Hazard ratio of all-cause and the major causes of mortality per SD change in domestic consumption (ex. smokeless) according to birth year. Adjusted for socioeconomic indicators in 1951 and 2001 and current $PM_{2.5.}$

For peer review only

Footnotes

Contributors: DIWP discovered the dataset on coal consumption in 1951/2 and together with CO conceived the study. HS and PA advised on the use of the geographical data and carried out the redistricting analysis. DIWP, CO and AJ were responsible for the statistical analysis. The manuscript was written by DIWP, CO, AJ, HS and SH. All authors participated in a critical revision of the text and approved the final manuscript. DIWP is responsible for the overall content as the corresponding author.

Funding: The work was supported by the Medical Research Council.

Competing interests: None declared.

Ethics approval: As the study was based on routinely collected mortality data no ethics approval was necessary.

Data sharing: Under the MRC's "Policy and Guidance on Sharing of Research Data from Population and Patient Studies" the dataset is available upon request to diwp@mrc.soton.ac.uk.

For peer review only

For peer review only

For peer review only - http://bmjopen.bmj.com/site/about/guidelines.xhtml

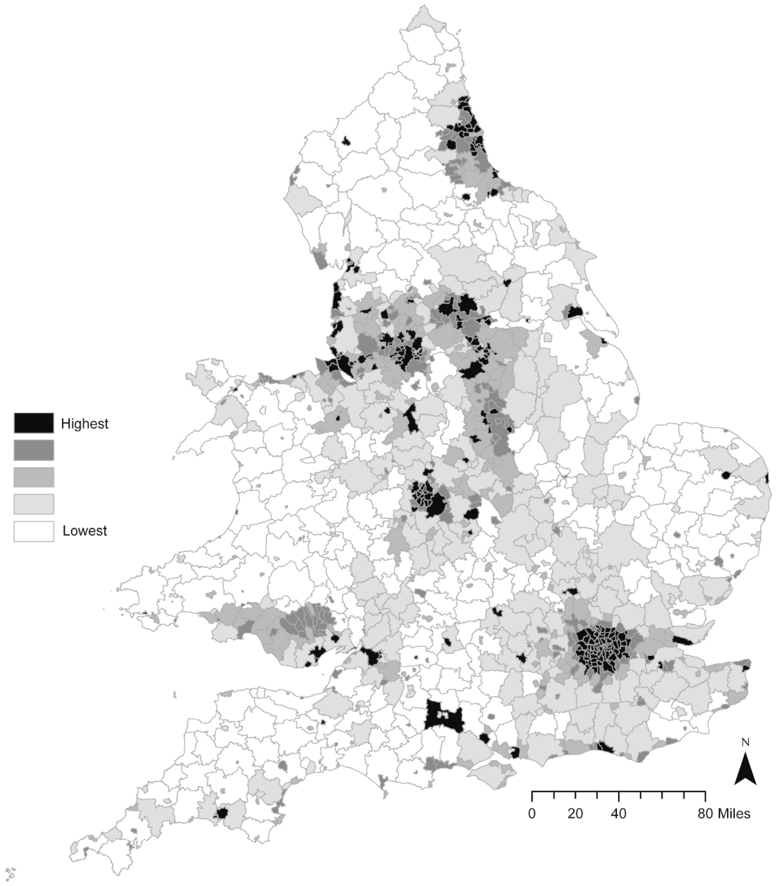

Domestic fuel consumption in England and Wales, 1951-52.

841x1188mm (151 x 151 DPI)

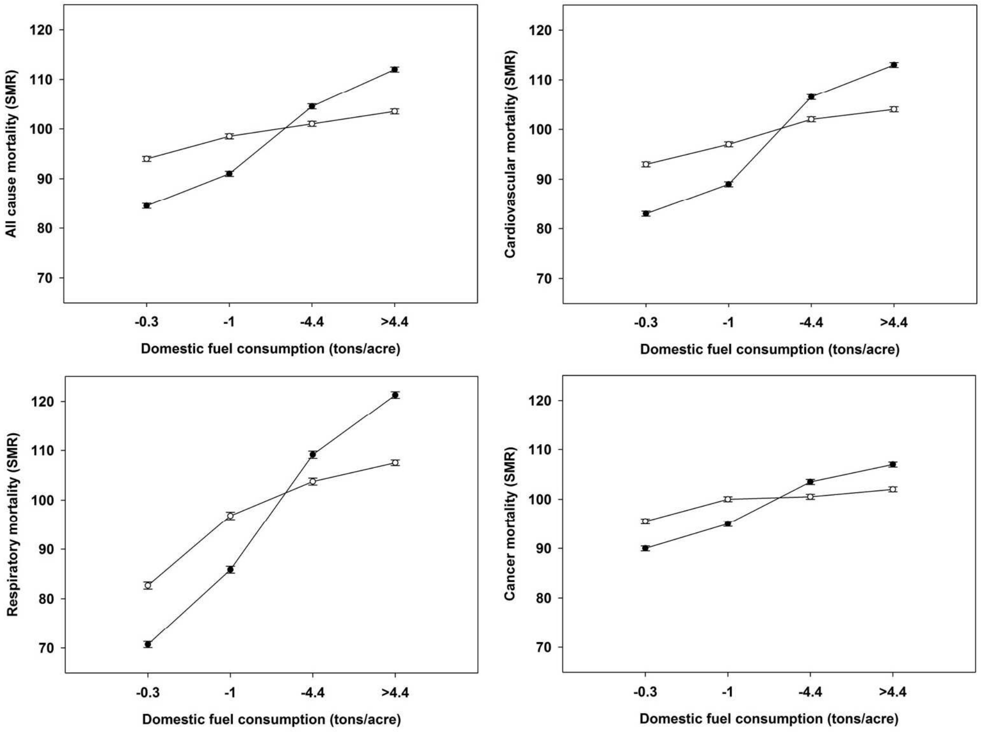

Figure 2: Association between quartiles of domestic fuel consumption (ex smokeless) and both all-cause and the major causes of mortality in men and women aged 35-74 in the 342 LGDs. (●unadjusted, ○ adjusted for socioeconomic indicators in 1951 and 2001 and current $PM_{2.5}$.; errors are 95% CI)

254x190mm (300 x 300 DPI)

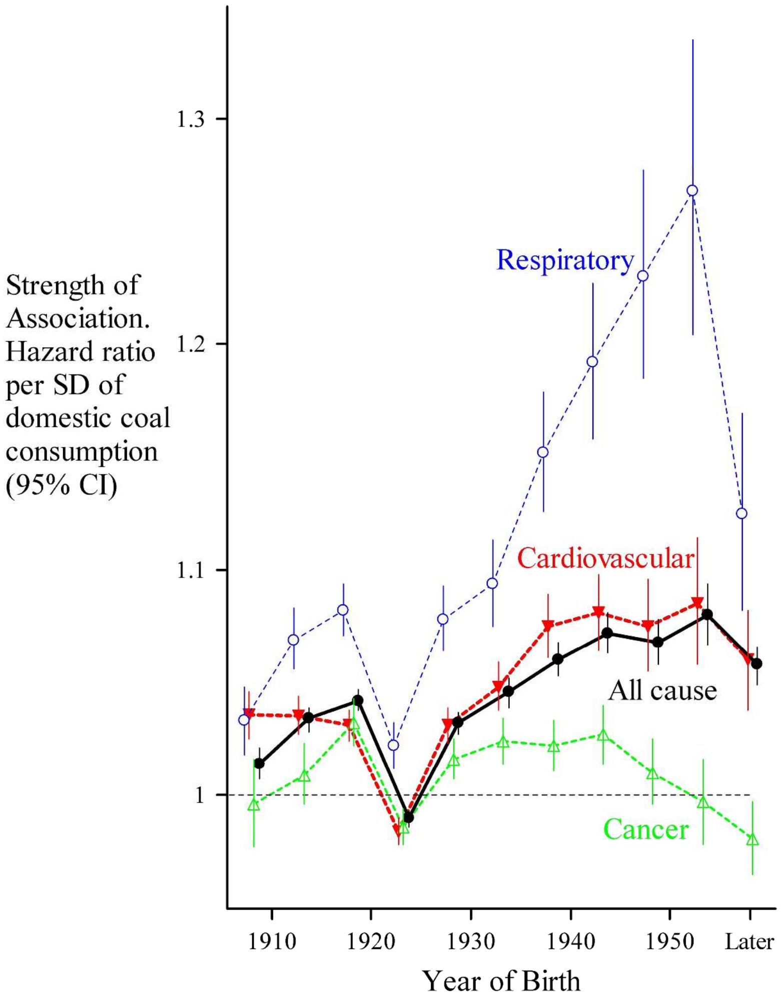

Figure 3: Hazard ratio of all-cause and the major causes of mortality per SD change in domestic consumption (ex. smokeless) according to birth year. Adjusted for socioeconomic indicators in 1951 and 2001 and current $PM_{2.5}$.

276x403mm (300 x 300 DPI)

Appendix

Cause of death	ICD 9 code	ICD 10 code	Number of deaths
**Cardiovascular disease**			
Ischaemic heart disease	410-414	I20-I25	710,979
Rheumatic heart disease	393-398	I05-I09	9,639
Cerebrovascular disease	430-438	I60-I69	264,853
Hypertension	401-405	I10-I15	84,208
**Respiratory disease**			
Chronic bronchitis and emphysema	490-492,496	J40-J44	105,690
Asthma	493	J45-J46	10,486
Pneumonia	480-486	J12-J18	107,848
Tuberculosis	010-018,137	A15-A19,B90	3,784
**Cancers**			
Lip, oral cavity and pharynx	140-149	C00-C14	22,992
Oesophagus	150	C15	65,854
Stomach	151	C16	49,634
Colon	153	C18	85,176
Rectosigmoid junction, rectum & anus	154	C19-C21	49,649
Liver, gall bladder and bile ducts	155,156	C22 – C24	31,240
Pancreas	157	C25	66,273
Larynx	161	C32	8,909
Trachea, bronchus & lung	162	C33,c34	335,217
Malignant melanoma	172	C43	20,045
Breast	174-175	C50	128,494
Cervix	180	C53	12,947
Uterus	179,182	C54-C55	14,315
Ovary	183	C56	47,895
Prostate	185	C61	54,521
Kidney	189	C64	33,232
Bladder	188	C67	32,353
Brain, Eye & CNS	191-192	C69-C72	43,811
Hodgkin's disease	201	C81	3,096
Non-Hodgkin's lymphoma	200,202	C82-C85	40,783
Multiple myeloma	203	C90	21,510
Leukaemia	204-208	C91-C95	33,526

For peer review only

Appendix 2

The Office of National Statistics provided latitude and longitude measurements for each of the 342 areas used in our analyses, and from these we derived inter-area distances in miles. We compared various simple models for the autocorrelation function, and found that the function which fitted the data best is given by

$$\rho(d) = \exp(-\lambda d^2)$$

Where d is the distance areas and λ is a parameter. The maximum likelihood estimate λ_{max} was -0.0772 with 95% profile likelihood based confidence limits of -0.0986 and -0.0647. This autocorrelation function is graphed in the Figure below. The autocorrelation is close to zero above distances of 10 miles. 107 of the 58,311 inter-area distances (0.2%) are below 5 miles and 586 (1.0%) are below 10 miles.

As described in the methods section, the coal consumption data (x) for each area ($x_1, x_2, \ldots\ldots x_{342}$) used in Table 2 were transformed using a Fisher-Yates normal transformation. Tabulating the mean values of the product $x_i x_j$ in bands derived according to the value of the inter-area distance d_{ij} confirms that the correlation falls rapidly in this way.

Calculations that incorporate the autocorrelation term λ_{max} into regression analyses suggest that the variance of regression coefficients will increase by about 14.8%, and hence that standard errors will increase by about 7.1%. Confidence interval widths will rise accordingly.

For peer review only

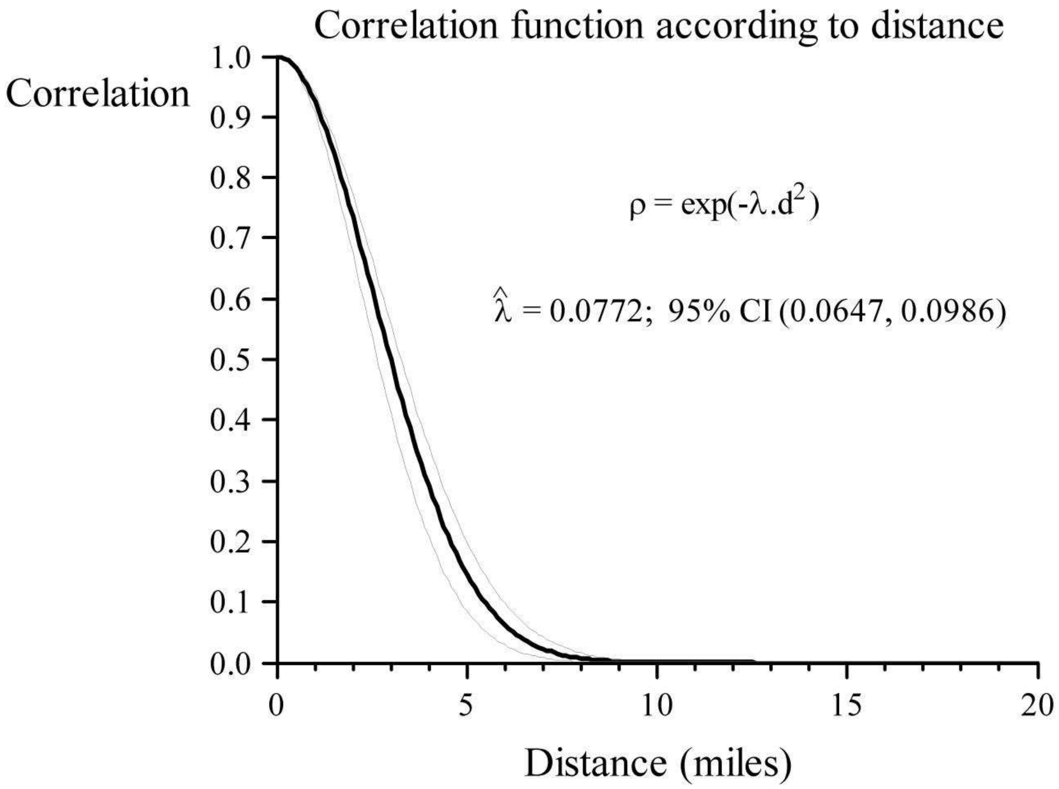
Correlation function according to distance
Correlation
1.0
0.9
0.8
0.7
0.6
0.5
0.4
0.3
0.2
0.1
0.0
ρ = exp(-λ.d²)
λ̂ = 0.0772; 95% CI (0.0647, 0.0986)
0
5
10
15
20
Distance (miles)

STROBE 2007 (v4) checklist of items to be included in reports of observational studies in epidemiology*
Checklist for cohort, case-control, and cross-sectional studies (combined)

Section/Topic	Item #	Recommendation	Reported on page #
**Title and abstract**	1	(*a*) Indicate the study's design with a commonly used term in the title or the abstract	2
		(*b*) Provide in the abstract an informative and balanced summary of what was done and what was found	2
**Introduction**			
Background/rationale	2	Explain the scientific background and rationale for the investigation being reported	4
Objectives	3	State specific objectives, including any pre-specified hypotheses	4
**Methods**			
Study design	4	Present key elements of study design early in the paper	5-6
Setting	5	Describe the setting, locations, and relevant dates, including periods of recruitment, exposure, follow-up, and data collection	5-6
Participants	6	(*a*) *Cohort study*—Give the eligibility criteria, and the sources and methods of selection of participants. Describe methods of follow-up<br>*Case-control study*—Give the eligibility criteria, and the sources and methods of case ascertainment and control selection. Give the rationale for the choice of cases and controls<br>*Cross-sectional study*—Give the eligibility criteria, and the sources and methods of selection of participants	5-6
		(*b*) *Cohort study*—For matched studies, give matching criteria and number of exposed and unexposed<br>*Case-control study*—For matched studies, give matching criteria and the number of controls per case	N/a
Variables	7	Clearly define all outcomes, exposures, predictors, potential confounders, and effect modifiers. Give diagnostic criteria, if applicable	5-6, 20 (appendix)
Data sources/ measurement	8*	For each variable of interest, give sources of data and details of methods of assessment (measurement). Describe comparability of assessment methods if there is more than one group	*5-6*
Bias	9	Describe any efforts to address potential sources of bias	6
Study size	10	Explain how the study size was arrived at	N/A
Quantitative variables	11	Explain how quantitative variables were handled in the analyses. If applicable, describe which groupings were chosen and why	6
Statistical methods	12	(*a*) Describe all statistical methods, including those used to control for confounding	6
		(*b*) Describe any methods used to examine subgroups and interactions	N/A
		(*c*) Explain how missing data were addressed	N/A

For peer review only

		(*d*) *Cohort study*—If applicable, explain how loss to follow-up was addressed<br>*Case-control study*—If applicable, explain how matching of cases and controls was addressed<br>*Cross-sectional study*—If applicable, describe analytical methods taking account of sampling strategy	N/A
		(*e*) Describe any sensitivity analyses	11
**Results**			
Participants	13*	(a) Report numbers of individuals at each stage of study—eg numbers potentially eligible, examined for eligibility, confirmed eligible, included in the study, completing follow-up, and analysed	7
		(b) Give reasons for non-participation at each stage	N/A
		(c) Consider use of a flow diagram	N/A
Descriptive data	14*	(a) Give characteristics of study participants (eg demographic, clinical, social) and information on exposures and potential confounders	15 (Table 1)
		(b) Indicate number of participants with missing data for each variable of interest	N/A
		(c) *Cohort study*—Summarise follow-up time (eg, average and total amount)	5
Outcome data	15*	*Cohort study*—Report numbers of outcome events or summary measures over time	7
		Case-control study—Report numbers in each exposure category, or summary measures of exposure	N/A
		Cross-sectional study—Report numbers of outcome events or summary measures	N/A
Main results	16	(*a*) Give unadjusted estimates and, if applicable, confounder-adjusted estimates and their precision (eg, 95% confidence interval). Make clear which confounders were adjusted for and why they were included	16,17,18,19 (Tables 2-4)
		(*b*) Report category boundaries when continuous variables were categorized	N/A
		(*c*) If relevant, consider translating estimates of relative risk into absolute risk for a meaningful time period	N/A
Other analyses	17	Report other analyses done—eg analyses of subgroups and interactions, and sensitivity analyses	N/A
**Discussion**			
Key results	18	Summarise key results with reference to study objectives	9
Limitations	19	Discuss limitations of the study, taking into account sources of potential bias or imprecision. Discuss both direction and magnitude of any potential bias	11,12
Interpretation	20	Give a cautious overall interpretation of results considering objectives, limitations, multiplicity of analyses, results from similar studies, and other relevant evidence	12
Generalisability	21	Discuss the generalisability (external validity) of the study results	12
**Other information**			
Funding	22	Give the source of funding and the role of the funders for the present study and, if applicable, for the original study on which the present article is based	25

*Give information separately for cases and controls in case-control studies and, if applicable, for exposed and unexposed groups in cohort and cross-sectional studies.

For peer review only

Note: An Explanation and Elaboration article discusses each checklist item and gives methodological background and published examples of transparent reporting. The STROBE checklist is best used in conjunction with this article (freely available on the Web sites of PLoS Medicine at http://www.plosmedicine.org/, Annals of Internal Medicine at http://www.annals.org/, and Epidemiology at http://www.epidem.com/). Information on the STROBE Initiative is available at www.strobe-statement.org.

For peer review only
